# Green‐Light Activation of Push–Pull Ruthenium(II) Complexes

**DOI:** 10.1002/chem.202000871

**Published:** 2020-04-30

**Authors:** Johannnes Moll, Cui Wang, Ayla Päpcke, Christoph Förster, Ute Resch‐Genger, Stefan Lochbrunner, Katja Heinze

**Affiliations:** ^1^ Department of Chemistry Johannes Gutenberg University of Mainz Duesbergweg 10–14 55128 Mainz Germany; ^2^ Division 1.2 Biophotonics Federal Institute for Materials Research and Testing (BAM) Richard Willstätter-Straße 11 12489 Berlin Germany; ^3^ Institut für Chemie und Biochemie Freie Universität Berlin Arnimallee 22 14195 Berlin Germany; ^4^ Institute for Physics and Department of Life, Light and Matter University of Rostock 18051 Rostock Germany

**Keywords:** luminescence, photocatalysis, photochemistry, photophysics, ruthenium

## Abstract

Synthesis, characterization, electrochemistry, and photophysics of homo‐ and heteroleptic ruthenium(II) complexes [Ru(cpmp)_2_]^2+^ (**2^2+^**) and [Ru(cpmp)(ddpd)]^2+^ (**3^2+^**) bearing the tridentate ligands 6,2’’‐carboxypyridyl‐2,2’‐methylamine‐pyridyl‐pyridine (cpmp) and *N*,*N*’‐dimethyl‐*N*,*N*’‐dipyridin‐2‐ylpyridine‐2,6‐diamine (ddpd) are reported. The complexes possess one (**3^2+^**) or two (**2^2+^**) electron‐deficient dipyridyl ketone fragments as electron‐accepting sites enabling intraligand charge transfer (ILCT), ligand‐to‐ligand charge transfer (LL'CT) and low‐energy metal‐to‐ligand charge transfer (MLCT) absorptions. The latter peak around 544 nm (green light). Complex **2^2+^** shows ^3^MLCT phosphorescence in the red to near‐infrared spectral region at room temperature in deaerated acetonitrile solution with an emission quantum yield of 1.3 % and a ^3^MLCT lifetime of 477 ns, whereas **3^2+^** is much less luminescent. This different behavior is ascribed to the energy gap law and the shape of the parasitic excited ^3^MC state potential energy surface. This study highlights the importance of the excited‐state energies and geometries for the actual excited‐state dynamics. Aromatic and aliphatic amines reductively quench the excited state of **2^2+^** paving the way to photocatalytic applications using low‐energy green light as exemplified with the green‐light‐sensitized thiol–ene click reaction.

## Graphical abstract

**Go green**! Push–pull substituted ruthenium(II) complexes strongly absorb green light and emit in the red to near‐infrared spectral region. Replacing X=NMe by X=CO in the ligand backbone boosts the luminescence quantum yield and ^3^MLCT lifetime (*Φ*=1.3 %; *τ*=477 ns). Amines reductively quench the long‐lived ^3^MLCT state enabling photoredox chemistry (light‐sensitized thiol–ene click reaction) with green light (see figure).

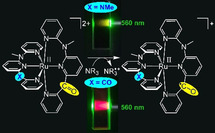

## Introduction

**Scheme 1:**
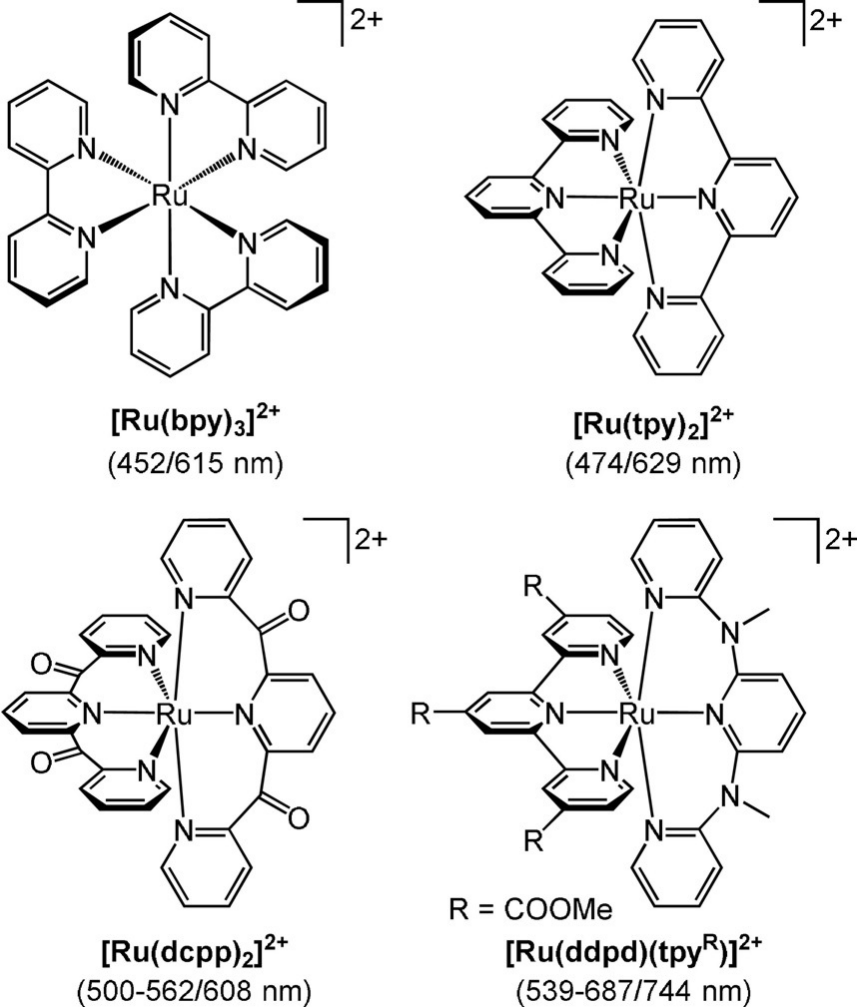
Benchmark ruthenium(II) complexes and their absorption/ emission band maxima.

Polypyridyl ruthenium(II) complexes (low spin d^6^ electron configuration)^1^, ^2^, ^3^, ^4^, ^5^, ^6^ often display phosphorescence from triplet metal‐to‐ligand charge transfer (^3^MLCT) states. Due to the comparably high ligand field splitting imposed by the metal ion and the ligands, non‐radiative relaxation via thermal population of metal‐centered states (^3^MC) can be retarded.^2^ The prototypical complex [Ru(bpy)_3_]^2+^ (bpy=2,2’‐bipyridine; Scheme 1) and its photophysical properties have been thoroughly investigated experimentally and theoretically.^2^, ^3^, ^4^, ^5^, ^6^, ^7^, ^8^, ^9^, ^10^, ^11^, ^12^, ^13^, ^14^ [Ru(bpy)_3_]^2+^ shows high phosphorescence quantum yields of 6.3 % (H_2_O),^15^ 9.5 % (CH_3_CN) at 298 K^15^, ^16^ and 38 % (methanol/ethanol glass) at 77 K,^5^, ^9^, ^17^ respectively. The favorable photophysical properties (such as high absorbance in the visible spectral region, high ^3^MLCT lifetimes of 620 ns (H_2_O)^5^, ^18^ and 850–1100 ns (CH_3_CN)^9^, ^18^ at 298 K, and high quantum yields) enable the application of [Ru(bpy)_3_]^2+^ and its analogues as chromophores in dye sensitized solar cells, in light emitting electrochemical cells and as photosensitizers.^19^, ^20^, ^21^, ^22^, ^23^, ^24^, ^25^, ^26^, ^27^, ^28^, ^29^ In fact, the majority of photosensitized redox reactions is still performed using [Ru(bpy)_3_]^2+^, which is further enabled by its favorable redox properties and its redox stability.

Despite the favorable quantum yields and lifetimes, blue light is required for efficient excitation. Further challenges of [Ru(bpy)_3_]^2+^ originate from the bidentate nature of the bpy ligands. The Δ/Λ chirality of the complex^30^, ^31^, ^32^, ^33^, ^34^, ^35^, ^36^, ^37^ can lead to diastereomeric di‐ or multinuclear complexes with sites of varying stereochemistry. Two differently substituted bpy ligands can form diastereomers even for mononuclear complexes [Ru(bpy)(bpy^R^)(bpy^R’^)]^2+^. Finally, bidentate ligands are comparably photolabile enabling photoisomerization of the complex or photosubstitution of the ligands.14a, 38, 39, 40 Replacing bpy by the tridentate ligand 2,2’:6’2’’‐terpyridine (tpy) yields achiral, comparably photostable [Ru(tpy)_2_]^2+^ complexes (Scheme 1).^41^ However, [Ru(tpy)_2_]^2+^ is essentially non‐phosphorescent at room temperature. This is due to the much lower ligand field splitting of this complex. The weaker ligand field splitting imposed by the tpy ligands significantly lowers the energy of ^3^MC states leading to efficient non‐radiative relaxation via these distorted ^3^MC states.^1^, ^2^, ^30^, ^37^, ^42^, ^43^, ^44^, ^45^

Eliminating thermal deactivation via the classical Jahn–Teller type and other distorted ^3^MC states is a key to higher phosphorescence quantum yields and longer excited‐state lifetimes.^14^ Complementary concepts to increase the energy gap between the emissive ^3^MLCT and the deactivating ^3^MC state have been devised, namely i) shifting the ^3^MC states to higher energy by an increase of the ligand field splitting with six‐membered ring chelate ligands allowing 180° *N*−Ru−N bond angles and hence optimal Ru−N orbital overlap and with strong σ‐donating/π‐accepting ligands and ii) lowering the energy of the ^3^MLCT state with electron‐deficient pyridine ligands. Examples successfully employing these concepts are [Ru(dcpp)_2_]^2+^ (dcpp=2,6‐bis(2‐carboxypyridyl)pyridine)^46^ and the heteroleptic complex [Ru(ddpd)(tpy^R^)]^2+^ (ddpd=*N*,*N*’‐dimethyl‐*N*,*N*’‐dipyridin‐2‐ylpyridine‐2,6‐diamine) (Scheme 1).^30^, ^37^ [Ru(dcpp)_2_]^2+^ with accepting dipyridyl ketone moieties and six‐membered chelate rings shows a very high phosphorescence quantum yield of 30 % in CH_3_CN at 298 K.^46^ [Ru(ddpd)(tpy^R^)]^2+^ with an electron‐donating ddpd ligand with six‐membered chelate rings and an electron‐deficient ester substituted tpy^R^ ligand has a phosphorescence quantum yield of 1.1 % in CH_3_CN.^30^, ^37^ On the other hand, cyclometalating tridentate ligands N^C^N or C^N^N in ruthenium(II) complexes with five‐membered chelate rings were unsuccessful in improving quantum yield and lifetime. This is due to an unfavorable distortion of their excited ^3^MLCT states and the presence of low‐energy ^3^LL'CT states composed of orthogonal tridentate L and L’ ligands resulting in symmetry‐forbidden and hence inefficient radiative transitions.^47^, ^48^, ^49^, ^50^, ^51^, ^52^

A further strategy to increase the ^3^MLCT lifetime beyond expanding the ^3^MC ‐^3^MLCT gap and increasing the barrier is to retard the ^3^MC to ground state (^1^GS) decay by increasing the energy of the ^3^MC/^1^GS crossing point.14b Although the ^3^MC state can be still thermally populated in this scenario, this would not contribute to the decay to the ground state provided that the crossing point is at high enough energy.^9^ Yet, such effects arising from the energies of surface crossing points are difficult to design as the shapes of potential energy surfaces have to be known and modified in addition to the minimum energies. Clearly, the rigidity of the ligands plays a significant role in the excited‐state distortions.

Herein, we introduce a novel tridentate mixed donor/acceptor ligand cpmp (cpmp=6,2’’‐carboxypyridyl‐2,2’‐methylamine‐pyridyl‐pyridine). This ligand enables six‐membered chelate rings combining the concepts of increasing the ^3^MC energy via a strong ligand field and of lowering the ^3^MLCT energy based on the electron‐deficient dipyridyl ketone fragment. In addition to the low‐energy MLCT states, the push–pull ligand cpmp can provide intra‐ligand transitions (^1, 3^ILCT), which might have a low energy as well. Furthermore, in contrast to complexes with merely five‐membered chelate rings of tridentate ligands (tpy type) and hence planar ligands with orthogonal orientation, the ligand cpmp can enable symmetry‐allowed transitions *between* the non‐orthogonal twisted tridentate ligands (^1, 3^LL'CT). This can in principle lead to emission from ^3^LL'CT states depending on their energy relative to the ^3^MLCT energy. In fact, the angle between planes of the central pyridines is only around 10–20° in [M(dcpp)_2_]^2+^, M(dcpp)(ddpd)]^2+^ and [M(ddpd)_2_]^2+^ complexes,^46^, ^53^, ^54^, ^55^, ^56^ instead of the approximate 90° angle in [M(tpy)_2_]^2+^‐type complexes.

We report the syntheses, structure, electrochemical as well as steady‐state and time‐resolved photophysical properties of the novel homoleptic [Ru(cpmp)_2_][PF_6_]_2_ (**2[PF_6_]_2_**) and heteroleptic ruthenium(II) complex [Ru(cpmp)(ddpd)][PF_6_]_2_ (**3[PF_6_]_2_**). We discuss the effects of ligand symmetry on the excited‐state types, their energies, their geometries and finally their excited‐state dynamics. The relevant excited states will be characterized by DFT and time‐dependent (TD‐) DFT calculations to gain a deeper understanding of the excited‐state dynamics. The new ruthenium(II) complexes **2[PF_6_]_2_** and **3[PF_6_]_2_** are applied in a light‐sensitized thiol–ene click reaction using low‐energy green light for excitation.

## Results and Discussion

### Synthesis and ground‐state structures

The push–pull ligand cpmp is prepared by lithiating 6‐bromo‐*N*‐methyl‐*N*‐(pyridin‐2‐yl)pyridyl‐2‐amine^57^ with *n*BuLi and quenching with 2‐cyanopyridine in moderate yields as yellow oil (Scheme 2; Supporting Information, Figures S1–S10).

**Scheme 2:**
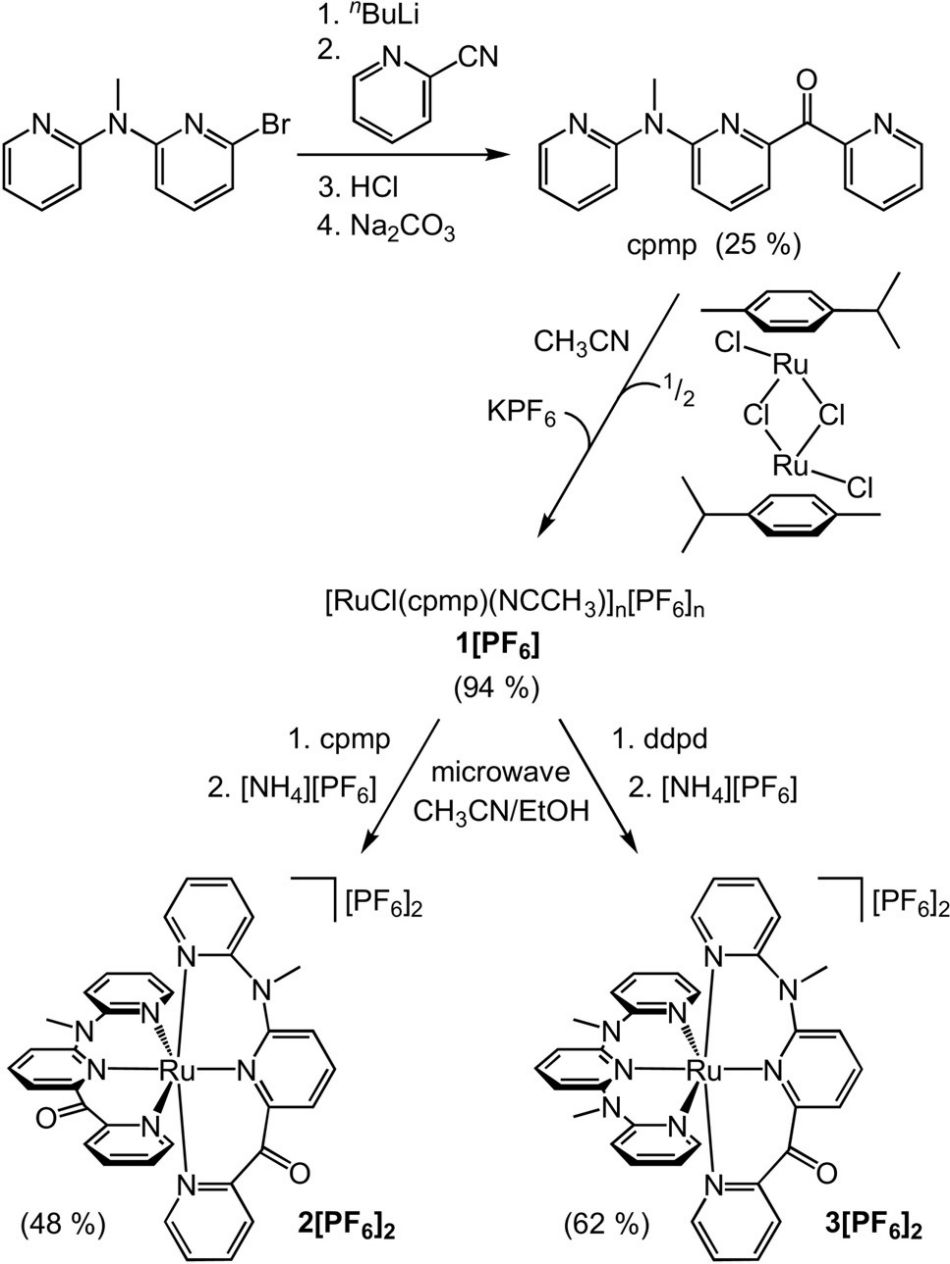
Synthesis of the ligand cpmp and its ruthenium(II) complexes **1[PF_6_]**, **2[PF_6_]_2_** and **3[PF_6_]_2_**.

Thermal ligand exchange with [(RuCl(*p*‐cymene)]_2_(*μ*‐Cl)_2_ 58 and counter ion exchange with KPF_6_ in acetonitrile gives the red‐purple cpmp complex [RuCl(cpmp)(NCCH_3_)]_*n*_[PF_6_]_*n*_ **1[PF_6_]** in very good yields (Supporting Information, Figures S11–S19). The coordinated CH_3_CN ligand in **1[PF_6_]** displays a broad band for the CN stretching vibration at 2275 cm^−1^ in the IR spectrum (KBr disk). ^1^H and ^13^C NMR resonances for the CH_3_ groups of the CH_3_CN ligands in the NMR spectra agree with a single CH_3_CN ligand coordinated to ruthenium. During the NMR experiments, the coordinated CH_3_CN ligand does not exchange with the solvent CD_3_CN (Supporting Information, Figures S11–S15). The chlorido ligand is observed in the electrospray ionization (ESI) mass spectrum and the [PF_6_]^−^ counter ion in the ^31^P NMR and IR spectra. The combined data are compatible with a dimeric arrangement (*n*=2) with bridging chlorido ligands.

Replacing the chlorido and acetonitrile ligands in **1[PF_6_]** by a further tridentate cpmp or ddpd ligand requires activation by microwave irradiation. Using cpmp and ddpd, the respective homo‐ and heteroleptic complexes **2^2+^** and **3^2+^** form. After counter ion exchange with [NH_4_][PF_6_], **2[PF_6_]_2_** and **3[PF_6_]_2_** are obtained as purple crystals in good to moderate yields (Supporting Information, Figures S20–S28, S29–S38). Obviously, **1[PF_6_]** is a convenient precursor for homo‐ and heteroleptic cpmp ruthenium(II) complexes. Further heteroleptic ruthenium(II) complexes with cpmp should be accessible via this general route as well employing other tridentate ligands such as tpy, H_2_‐tpda,^55^, ^59^ R_2_‐tpda,^55^ ddpd‐NH_2_ 30 or dcpp.^46^, ^54^, ^60^

The compositions of **2[PF_6_]_2_** and **3[PF_6_]_2_** are confirmed by (high‐resolution) ESI mass spectrometry (Supporting Information, Figures S26, S36) and elemental analyses. Multinuclear and correlation NMR spectroscopic data suggest a single isomer being present in solution as only a single set of ^1^H and ^13^C NMR resonances is observed for both cations (Supporting Information, Figures S20–S24, S29–S34). Although the flexible ddpd ligand can enable a facial coordination,^53^ dcpp and cpmp ligands are quite rigid at the dipyridyl ketone units and consequently a meridional coordination of the tridentate ligands is very likely in both cases. This is also consistent with the NMR data.

**Figure 1:**
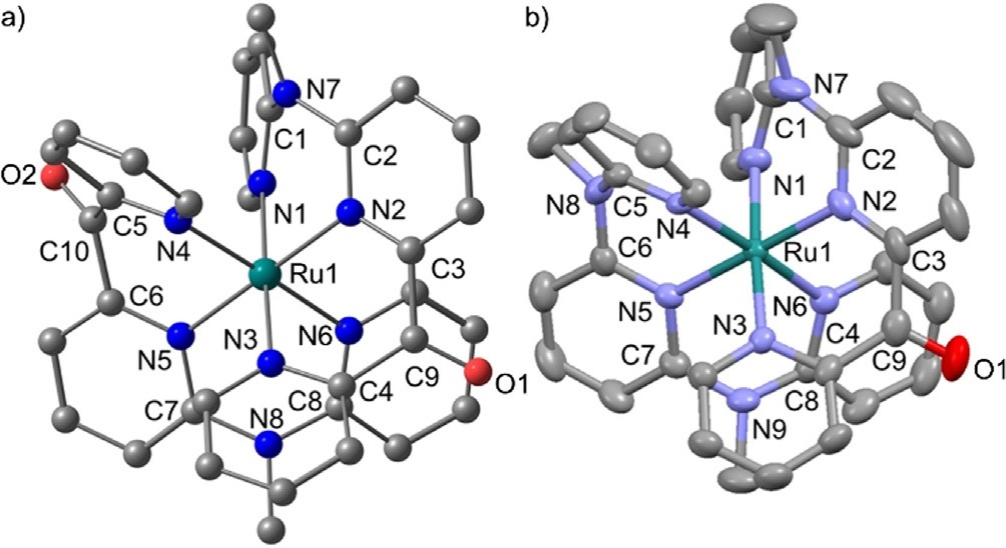
a) Molecular structure of **2^2+^** determined by DFT geometry optimization and b) **3A^2+^** determined by single crystal XRD, ellipsoids set at 50 % probability. Hydrogen atoms are omitted for clarity.

The geometric structure of **3^2+^** with meridional coordination was experimentally confirmed by X‐ray diffraction analysis of a single crystal of **3[PF_6_]_2_** (Figure 1). The unit cell of **3[PF_6_]_2_** contains two independent cations **3A^2+^** and **3B^2+^** (Table 1). Furthermore, the geometries of both dications **2^2+^** and **3^2+^** were calculated by DFT methods (Figure 1, Table 1; CPCM(acetonitrile)‐B3LYP‐D3BJ‐ZORA/old‐TZVP). In all cases, a nearly perfect [RuN_6_] octahedral coordination geometry is found with Ru−N bond lengths of 2.05 to 2.08 Å for the terminal pyridine rings of each ligand and slightly shorter Ru−N bonds for the central pyridine N atoms of each ligand (Table 1) as typically observed for [M(ddpd)_2_]^2+^, [M(dcpp)_2_]^2+^ or [M(dcpp)(ddpd)]^2+^ complexes.^46^, ^53^, ^54^, ^55^, ^56^ The *N*‐Ru−N bond angles are very close to 90° for the dipyridyl ketone moieties and slightly smaller for the more flexible dipyridyl methyl amine moieties of the cpmp and ddpd ligands. The shape parameter *S*(OC‐6)^61^ quantifying the deviation from an ideal octahedron is close to zero, corroborating the high local [RuN_6_] symmetry in all cases (Table 1) as expected for a metal ion with low‐spin d^6^ electron configuration. The steric strain, as measured from the pyramidalization of the bridging nitrogen atoms, inversely correlates to the degree of planarization PL/%=100×[∑(X‐*N*‐Y)–3×109.5°]/ [3×120.0°–3×109.5°]. Expectedly, the carbonyl carbon atom is coordinated in an essentially planar fashion while the bridging nitrogen atom is more pyramidalized (Table 1).

**Table 1:** Selected bond lengths [Å], angles [°], planarization PL [%] and continuous shape parameter *S*(OC‐6)^61^ of **2^3+^** and **3^2+^** from XRD analysis (as PF_6_^−^ salt) and by DFT calculations.

	**2^2+^** (DFT)	**3^2+^** (DFT)	**3A^2+^** (XRD)	**3B^2+^** (XRD)
Ru1−N1	2.080	2.081	2.085(3)	2.069(4)
Ru1−N2	2.048	2.047	2.023(3)	2.040(4)
Ru1−N3	2.072	2.071	2.045(3)	2.064(3)
Ru1−N4	2.081	2.077	2.061(3)	2.077(3)
Ru1−N5	2.049	2.054	2.044(3)	2.036(3)
Ru1−N6	2.072	2.076	2.062(3)	2.063(3)
N1‐Ru1‐N2	87.73	87.69	87.06(13)	87.54(15)
N2‐Ru1‐N3	90.23	90.26	90.16(13)	89.75(14)
N4‐Ru1‐N5	90.22	88.42	87.94(13)	87.62(14)
N5‐Ru1‐N6	87.74	88.39	87.91(14)	88.66(13)
PL(N7)	87.4	87.3	88.9	86.7
PL(N8)	87.3	86.8	87.0	84.1
PL(N9)		86.8	88.9	87.6
PL(C9)	97.8	97.7	97.8	97.5
PL(C10)	97.8			
*S*(OC‐6)	0.04	0.09	0.06	0.08
Ru1**⋅⋅⋅**F1			4.176	4.270
Ru1**⋅⋅⋅**F2			4.322	–
Ru1**⋅⋅⋅**N1${}_{\left(\mathrm{CH}{}_{3}\mathrm{CN}\right)}$			5.267	5.137
Ru1**⋅⋅⋅**O1_(neighboring molecule)_			4.655	4.822

In the solid state, the second coordination sphere of **3A^2+^** and **3B^2+^** is composed of hexafluorophosphate ions, co‐crystallized acetonitrile and the carbonyl group of a neighboring complex with closest contacts of Ru**⋅⋅⋅**[F(PF_5_)]^−^, Ru**⋅⋅⋅**N(CCH_3_) and Ru**⋅⋅⋅**O ranging from 4.18 to 5.27 Å (**3A^2+^**) and 4.27 to 5.14 Å (**3B^2+^**) (Table 1).

In the IR spectra of **2[PF_6_]_2_** and **3[PF_6_]_2_** (Supporting Information, Figures S27, S37), the characteristic PF stretching vibrations indicate the successful counter ion exchange. This is also consistent with the ^31^P NMR spectra of **1[PF_6_]**, **2[PF_6_]_2_** and **3[PF_6_]_2_** which show the typical resonance of [PF_6_]^−^ (Supporting, Information, Figures S16, S25, S35).

In the solid state, the IR absorption band of the C=O stretching vibration of the cpmp ligand is found at 1679 cm^−1^ and that of **1^+^** at 1672 cm^−1^ (Supporting Information, Figures S8, S18). On the other hand, the dicationic complexes **2^2+^** and **3^2+^** show the CO band at lower energy (Supporting Information, Figures S27, S37; 1664 and 1666 cm^−1^, respectively), suggesting π back‐donation of ruthenium(II) into the π*(CO) orbitals. As the homoleptic complex **2^2+^** merely displays a single IR band for the two CO groups, these two oscillators are decoupled.

### Electrochemistry

**Figure 2:**
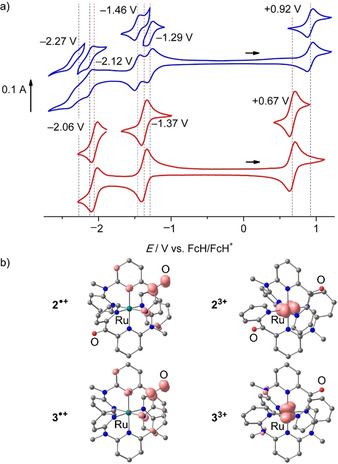
a) Cyclic voltammograms of **2[PF_6_]_2_** (blue) and **3[PF_6_]_2_** (red), 1 mm in acetonitrile, 0.1 m [*n*Bu_4_N][PF_6_] and b) DFT optimized geometries and spin densities (isosurface at 0.01 a. u.) of the radical cations **2** ^.**+**^ and **3** ^.**+**^ and the ruthenium(III) complexes **2^3+^** and **3^3+^**.

Both complexes **2^2^** ^+^ and **3^2+^** are reversibly oxidized in their cyclic and square wave voltammograms at +0.92 V and +0.67 V vs. ferrocene, respectively (Figure 2 a; Supporting Information, Figures S39, S40). The oxidation processes are metal‐centered as confirmed by the DFT calculations on the trications **2^3+^** and **3^3+^** (Figure 2 b). The oxidation potential of **2^2+^**/**2^3+^** is higher than that of **3^2+^**/**3^3+^** by 0.25 V due to the electron withdrawing effect of two CO groups in **2^2+^** instead of merely a single one in **3^2+^**. The even higher oxidation potential of the [Ru(dcpp)_2_]^2+^/[Ru(dcpp)_2_]^3+^ couple (+1.10 V vs. ferrocene) with four CO groups is consistent with this trend.^46^ The Ru^II^/Ru^III^ oxidations of **2^2+^** and **3^2+^** are perfectly reversible on the time scale of IR spectroelectrochemistry in acetonitrile (Supporting Information, Figures S41, S42). Relative to the parent ruthenium(II) complexes, the bands of the CO stretching vibrations of the ruthenium(III) complexes **2^3+^** and **3^3+^** shift to higher energy by 19 and 21 cm^−1^, respectively, implying weaker π back‐bonding in the ruthenium(III) complexes.

The bis(cpmp) complex **2^2+^** exhibits two reversible reduction waves at −1.29 V and −1.46 V (Figure 2 a). These processes are assigned to the reduction of the individual cpmp ligands (Figure 2 b). The reduction wave of the single cpmp ligand in **3^2+^** appears at −1.37 V (Figure 2 a). The first two reductions of [Ru(dcpp)_2_]^2+^ are very similar to those of **2^2+^** (Table 2).^46^ The reduction waves of **2^2+^** and **3^2+^** below −2 V are assigned to a second reduction of the CO groups in the cpmp ligands. Reduction of cpmp itself to cpmp**^.^** ^−^ at −1.99 V is only quasireversible (Supporting Information, Figure S10) likely due to subsequent pinacol coupling reactions.^62^, ^63^ Obviously, the cationic nature of **2^.+^** and **3^.+^** and steric hindrance prevent an analogous coupling reaction, at least on the time scale of the electrochemical experiments. Chemical reduction of **2^2+^**/**3^2+^** to **2^.+^**/**3^.+^** by one equivalent decamethylcobaltocene decreases the intensity of the CO stretching modes in the IR spectra partially (**2^2+^**/**2^.+^**) and nearly completely (**3^2+^**/**3^.+^**), respectively, reflecting the number of CO groups in the complexes (Figure 2 b; Supporting Information, Figures S43, S44). The band of the CO stretching vibration of the reduced cpmp in the radical cations **2^.+^** and **3^.+^** disappears due to a large shift to lower energy into the fingerprint region (which is obscured by solvent absorptions). This is consistent with the additional electron being localized on a CO group of a cpmp ligand. DFT calculations on **2^.+^** and **3^.+^** support this view as the spin density is essentially localized on the CO group (Figure 2 b). Furthermore, the calculated C−O bond length increases from 1.217 to 1.274 Å and from 1.218 to 1.277 Å on the reduced ligand for **2^2+^**/**2^.+^** and **3^2+^**/**3^.+^**, respectively (Figure 2 b).

**Table 2:** Electrochemical properties of cpmp, [Ru(bpy)_3_][PF_6_]_2_, [Ru(dcpp)_2_][PF_6_]_2_, **2[PF_6_]_2_** and **3[PF_6_]_2_**. *E*_1/2_ in V vs. ferrocene/ferrocenium.

	Reductions				Oxidation
cpmp				−1.99^[a]^	+0.97^[a]^
[Ru(bpy)_3_]^2+[64]^		−2.20	−1.93	−1.74	+0.84
[Ru(dcpp)_2_]^2+[46]^	−2.09	−1.84	−1.51	−1.34	+1.10
**2^2+^**	−2.27	−2.12	−1.46	−1.29	+0.92
**3^2+^**	−2.06		−1.37		+0.67

[a] Irreversible, *E*_p_ given.

**Figure 3:**
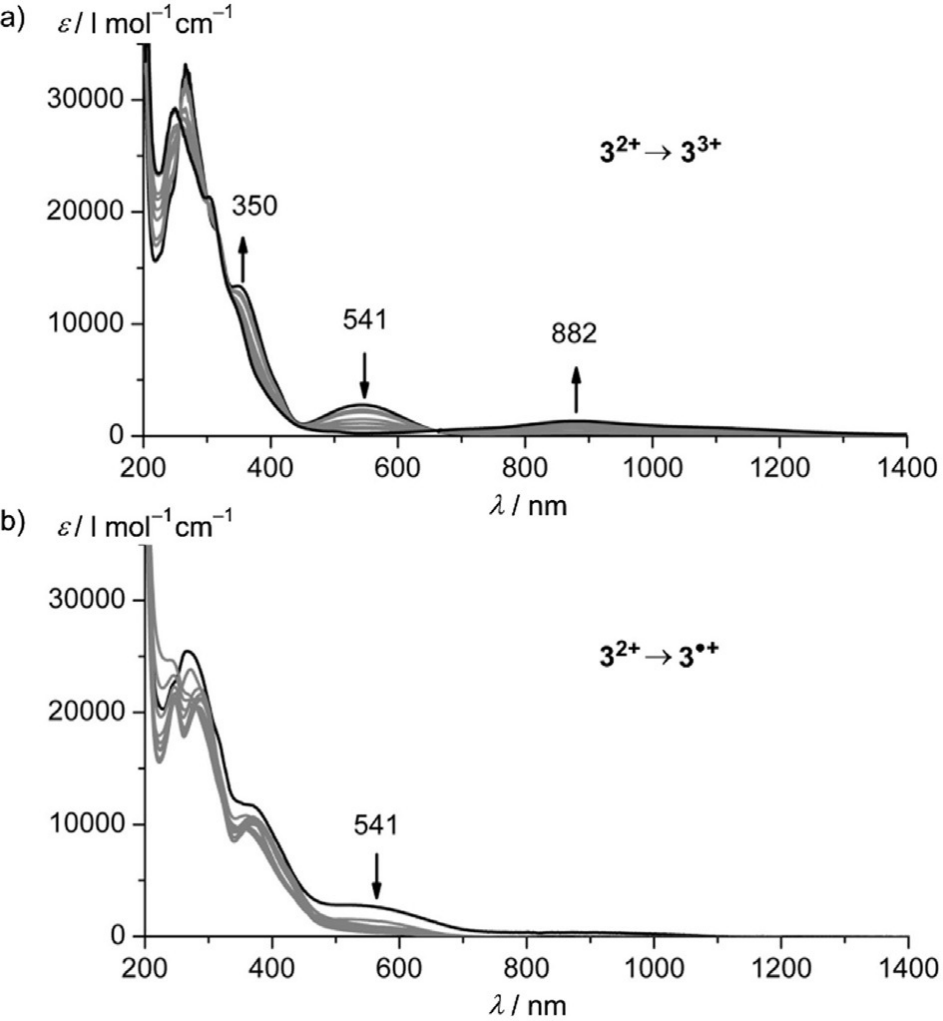
UV/Vis/NIR absorption spectra of **3[PF_6_]_2_** in acetonitrile with 0.1 m [*n*Bu_4_N][PF_6_] at 298 K collected during a) electrochemical oxidation and b) electrochemical reduction.

Oxidative UV/Vis/NIR spectroelectrochemical experiments of **3^2+^** in CH_3_CN containing 0.1 m [*n*Bu_4_N][PF_6_] confirm the reversibility of the oxidation (Figure 3 a). The MLCT band of **3^2+^** bleaches, while a ligand‐to‐metal charge transfer (LMCT) band at 882 nm concomitantly appears resulting in an isosbestic point at 661 nm. On the other hand, spectroelectrochemical reduction of **3^2+^** is not fully reversible on the time scale of spectroelectrochemistry (Figure 3 b). Apart from the MLCT bleach, no prominent spectral changes in the visible spectral region evolve during reduction of **3^2+^** (Figure 3 b). Spectroelectrochemical oxidation of **2^2+^** to **2^3+^** leads to the bleaching of the MLCT band at 549 nm and an increase of the LMCT band of **2^3+^** at 1039 nm, while reduction of **2^2+^** is not fully reversible as well on this time scale (Supporting Information, Figure S45).

### Photophysical properties

The cpmp ligand possesses several ππ* absorption bands and bands with ILCT character, namely CT from the electron‐rich pyridyl methyl amine to the electron‐poor dipyridyl ketone moiety according to TD‐DFT calculations (distal and proximal ILCT; Supporting Information, Figure S9). Irradiating into the (proximal) ILCT band at 354 nm leads to fluorescence at 455 nm (Supporting Information, Figure S9).

**Figure 4:**
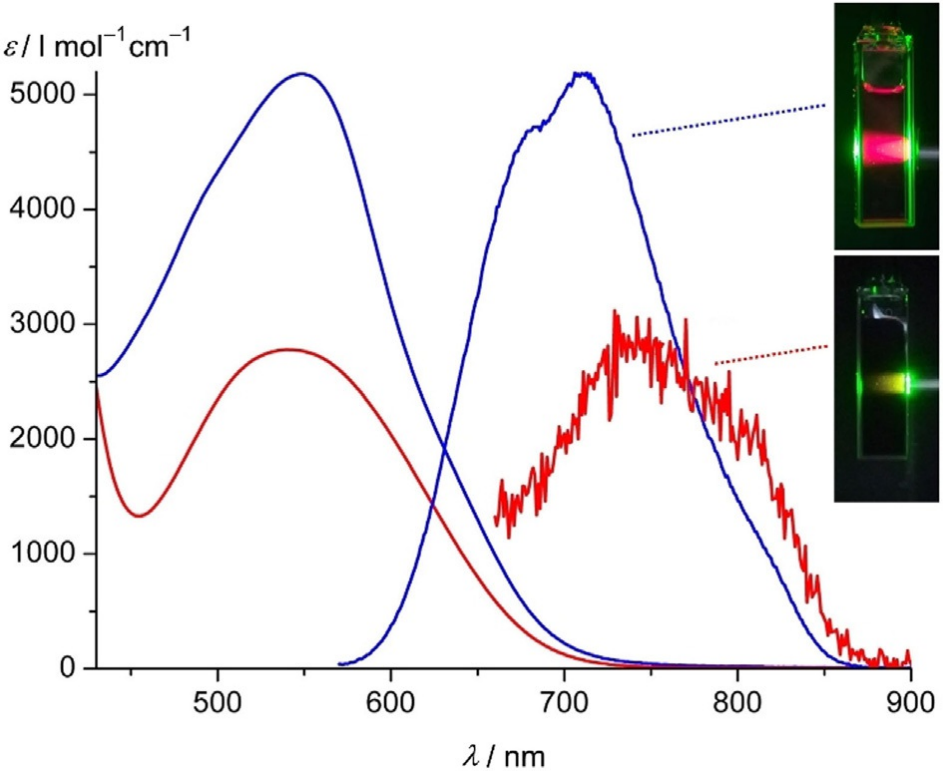
UV/Vis/NIR absorption and emission spectra (scaled to the respective MLCT absorption band) of **2[PF_6_]_2_** (blue) and **3[PF_6_]_2_** (red) in acetonitrile at 298 K (*λ*_exc_=544 nm). The photos display cuvettes of acetonitrile solutions of **2[PF_6_]_2_** and **3[PF_6_]_2_** under irradiation with green light (560 nm).

UV/Vis/NIR absorption spectra of **2[PF_6_]_2_** and **3[PF_6_]_2_** recorded in acetonitrile at room temperature are depicted in Figure 4 (Supporting Information, Figures S28, S38 for complete spectra). According to TD‐DFT calculations and derived charge transfer numbers for the transitions (Supporting Information, Figures S28, S38), absorption bands at 342 nm (**2^2+^**) and 350 nm (**3^2+^**) are mainly composed of MLCT transitions and weaker ILCT transitions at the lower energy side (≈390–400 nm). Weak LL'CT transitions are calculated at 402 nm for **2^2+^** and at lower energy (497 nm) for **3^2+^**. According to TD‐DFT calculations, the characteristic absorption bands of **2^2+^** and **3^2+^** at 549 and 541 nm are composed of six and three transitions with MLCT character, respectively (Table 3, Supporting Information, Figures S28, S38). The intensity of the MLCT band of **2^2+^** is significantly higher due to the larger number of acceptor sites, namely the dipyridyl ketones of the two cpmp ligands and consequently more MLCT transitions (Figure 4). Although the MLCT band of **3^2+^** overlaps with the low‐energy LL'CT transition calculated at 497 nm, all ILCT and LL'CT bands are higher in energy than the low‐energy MLCT transitions. Hence, the MLCT states should dominate the excited‐state dynamics of the complexes when excited with low energy light.

**Table 3:** Absorption and emission wavelengths, *E*_00_ calculated from low temperature emission spectra and excited‐state lifetimes of **2[PF_6_]_2_**, **3[PF_6_]_2_** and benchmark complexes in deaerated acetonitrile at 298 K and in butyronitrile at 77 K (if not stated otherwise), respectively.

	*λ*_abs_ [nm] (298 K)	*ϵ* [m ^−1^ cm^−1^] (298 K)	*λ*_em_ [nm] (298 K)	*Φ* [%] (298 K)	*λ*_em_ [nm] (77 K)	*E*_00_ [eV]	*τ* [ns] (298 K)
**2^2+^**	549	5200	709	1.3	693	1.79	477
**3^2+^**	541	2800	755	0.04	740	1.68	56
[Ru(bpy)_3_]^2+^	452^[a, 9]^	13 000^9^	615^[a, 9]^	9.5^15^, ^16^	582^[b, 9]^	2.15^[c, 66]^	1100^[a]^
[Ru(tpy)_2_]^2+^	474^41^	10 400^41^	629^41^	<5×10^‐4^ 41	598^41^	2.09^[c, 66]^	0.25
[Ru(ddpd)(tpy^R^)]^2+^R=COOMe)	539, 603, 687^30^	6360, 3100 (sh), 800 (sh)^30^	744^30^	1.1^[d, 30]^	728^30^	1.70^30^	841
[Ru(dcpp)_2_]^2+^	500, 522, 562^46^	5428, 6425, 2604^46^	608^46^	30^46^	613^46^	2.02^46^	3300

[a] 293 K. [b] in MeOH/EtOH 4:1. [c] in EtOH/MeOH 4:1. [d] 295 K.

Upon excitation at 544 nm, both complexes are phosphorescent in acetonitrile at room temperature with broad emission bands peaking at 709 nm and 755 nm, respectively (Figure 4). The excitation spectrum of **2^2+^** matches the absorption spectrum (Supporting Information, Figure S28a), confirming that the emission originates from **2^2+^**. The luminescence quantum yield of *Φ*=1.3 % measured with an integrating sphere in deaerated acetonitrile drops to 0.88 % under aerated conditions suggesting quenching of the excited triplet state by triplet oxygen (68 % quenching). Concomitantly, the excited‐state lifetime decreases from *τ*=477 ns to *τ*=237 ns under ambient conditions (acetonitrile, *λ*_exc_=544 nm, Supporting Information, Figure S46a) confirming the triplet character of the excited state.

The phosphorescence quantum yield of **3^2+^** of *Φ*=0.04 %, measured relative to that of **2^2+^** in acetonitrile at 298 K, is 33 times lower. The excitation spectrum of **3^2+^** only fits to the low‐energy ^1^MLCT band, while the higher energy transitions around 267 and 350 nm do not significantly evolve to the emissive ^3^MLCT state (Supporting Information, Figure S38a). Obviously, the ^3^MLCT state of **3^2+^** is only populated efficiently via the low‐energy ^1^MLCT states but not from the high‐energy ^1^MLCT/^1^LL'CT states suggesting an alternate excited‐state decay behavior from these higher energy states as compared to **2^2+^**. This alternate decay path is possibly enabled by the higher flexibility of the ddpd ligand. The ^3^MLCT excited‐state lifetime of **3^2+^** was determined using the lifetime spectrometer (*τ*=45 ns, Supporting Information, Figure S46b) and confirmed by streak camera measurements (*τ*=56 ns) at room temperature in CH_3_CN (Supporting Information, Figure S47). In both experiments, a second component with higher lifetime (lifetime spectrometer: 294 ns; by streak camera: 468 ns) was present. We ascribe the second component to some trace impurity, possibly traces of **2^2+^** below the NMR detection limit.

**Figure 5:**
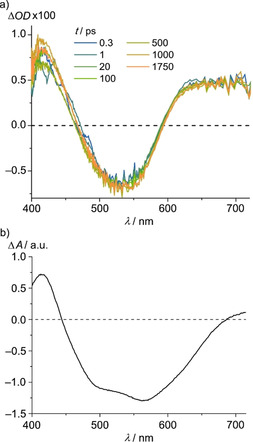
a) fs‐Transient absorption spectra of **2[PF_6_]_2_** in acetonitrile at different delay times after excitation at 540 nm at 293 K and b) estimated difference spectrum^67^ derived from absorption spectra of **2^2+^**, **2^3+^** and **2** ^.**+**^, prepared electrochemically in acetonitrile with 0.1 m [*n*Bu_4_N][PF_6_] at 298 K by “scaled [1/2 (**2^3+^**+**2^.+^**) −**2^2+^**]”.

Transient absorption spectroscopy was used to explore the initial dynamics of **2^2+^** and **3^2+^** after the excitation pulse (540 nm; Figure 5 a, Supporting information, Figures S48–S50). For **2^2+^**, a ground‐state bleach at 535 nm is observed together with excited‐state absorptions around 400 nm and above 600 nm. This fits to MLCT states with the MLCT band of the ground‐state bleached and the LMCT band of the excited state appearing as suggested by the estimated difference spectrum derived from the absorption spectra of **2^2+^**, **2^.+^** and **2^3+^** (Figure 5 b),^67^ prepared by electrochemical oxidation and reduction (Supporting Information, Figure S45). Intersystem crossing from the ^1^MLCT to the ^3^MLCT state occurs within the time resolution of the instrument (100 fs). The populated ^3^MLCT state features a lifetime in the nanosecond range. This fits to the TCSPC and streak camera measurements (Supporting Information, Figures S46a, S47a). The heteroleptic complex **3^2+^** displays an analogous behavior, yet superimposed by the spectral characteristics of trace impurities of **2^2+^** (Supporting Information, Figure S48).

In frozen butyronitrile solution at 77 K, the emission bands of **2^2+^** and **3^2+^** at 693 and 740 nm, respectively, are sharper with less vibrational broadening (Supporting Information, Figure S51; Table 3). From these data, the triplet excited‐state energies are calculated as 1.79 and 1.68 eV, respectively. The lower ^3^MLCT energy of the latter might also contribute to the low quantum yield of **3^2+^** due to its higher non‐radiative decay rate constants according to the energy gap law.^68^, ^69^

Compared to [Ru(tpy)_2_]^2+^,^36^, ^41^ the emission quantum yields of **2^2+^** and **3^2+^** are significantly higher in spite of the lower ^3^MLCT energy and hence potentially increased non‐radiative relaxation (based on the energy gap law), demonstrating the usefulness of the “large ligand field” concept. The emission quantum yield of the push–pull complex **2^2+^** is similar to that of the heteroleptic donor–acceptor complex [Ru(ddpd)(tpy^R^)]^2+^ bearing only a single tridentate ligand with large bite angles (Scheme 1). In the complex series **3^2+^**, **2^2+^**, [Ru(dcpp)_2_]^2+^ with exclusively six‐membered chelate rings, but an increasing number of dipyridyl ketone units, quantum yields increase by factors of 35 and 23, and the lifetimes by 8.5 and 6.9, respectively. The enhancement in quantum yield in this series parallels the increasing ^3^MLCT energies (Table 3) in accordance with the energy gap law.^68^, ^69^ This again demonstrates that merely lowering the ^3^MLCT energies to increase the ^3^MC ‐^3^MLCT gap is counterproductive. However, the ^3^MLCT energies of **2^2+^** and **3^2+^** differ only by 0.11 eV and hence further aspects must be additionally relevant for the excited‐state dynamics of **2^2+^** and **3^2+^**.

To gain a deeper insight in the excited‐state landscape, geometry optimizations based on DFT calculations on the singlet ground states (^1^GS), the ^3^MLCT and ^3^MC states of **2^2+^** and **3^2+^** were performed on the CPCM(acetonitrile)‐B3LYP‐D3BJ‐ZORA/old‐TZVP level of theory. The calculated ^3^MLCT energies are slightly lower than the experimental ones. Yet, the calculations correctly reproduce the order of the ^3^MLCT energies with that of **2^2+^** being higher by 0.16 eV than that of **3^2+^**. On the other hand, the calculated ^3^MC state minima of **2^2+^** and **3^2+^** are very similar in energy. This leads to a larger ^3^MC ‐^3^MLCT energy difference for **3^2+^** (0.77 eV) than for **2^2+^** (0.57 eV), which would imply a facilitated non‐radiative decay via the ^3^MC state in **2^2+^**, which is not observed.

**Figure 6:**
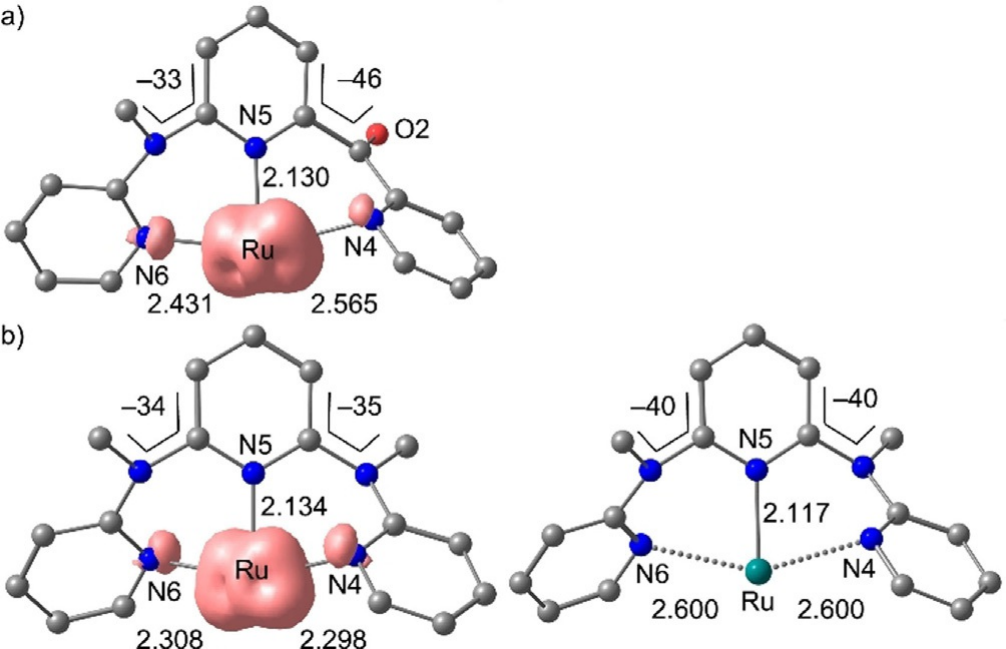
DFT‐optimized geometries of the ^3^MC states of a) **2^2+^** and b) **3^2+^** showing the relevant distances and angles of the tridentate ligand involved in the Jahn–Teller distortion (spin density plotted with an isosurface value of 0.01 a.u.). The essentially unaffected ancillary cpmp ligand is not shown for clarity. The two displayed geometries of **3^2+^** are nearly isoenergetic.

**Figure 7:**
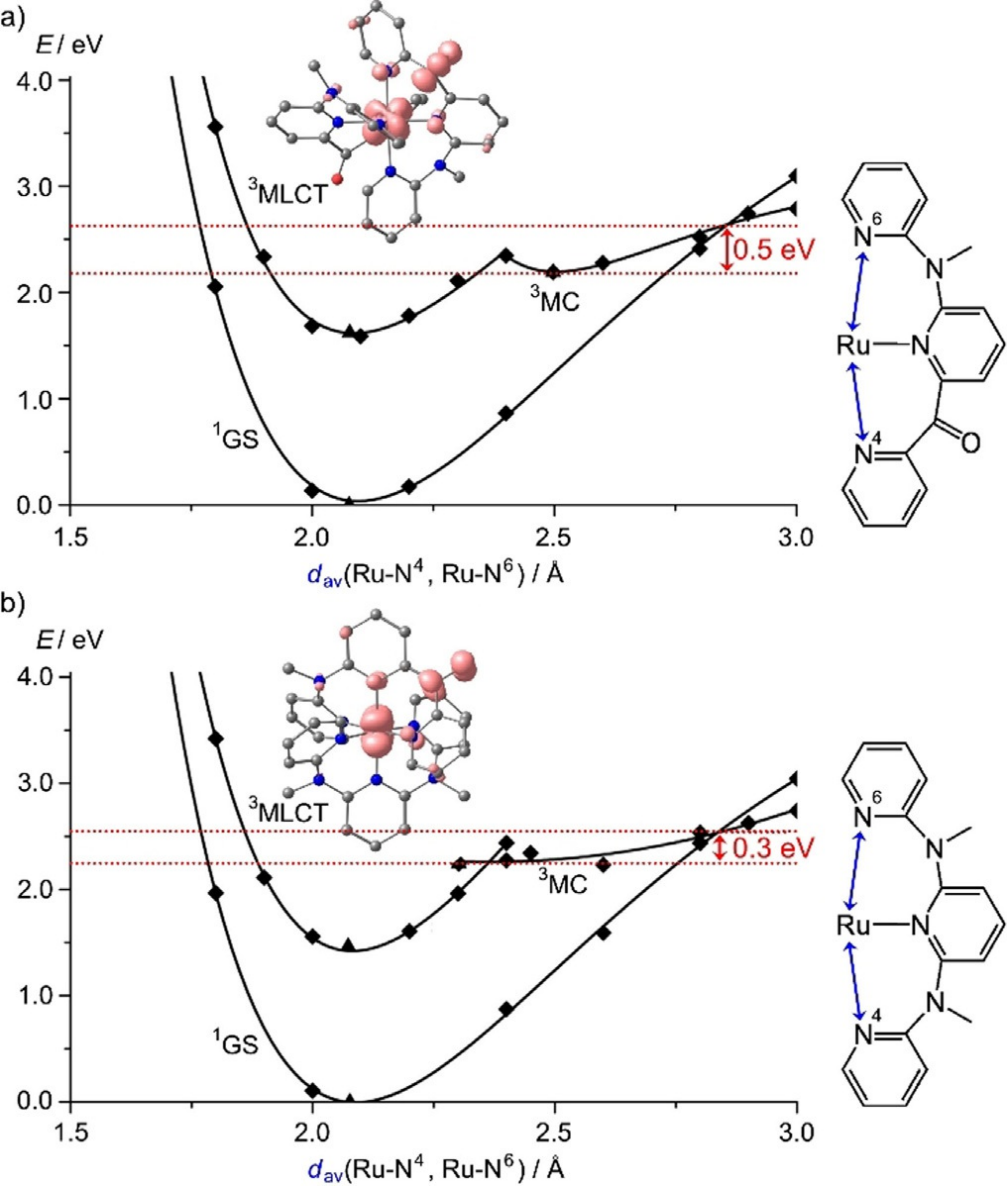
Calculated potential energy diagrams of a) **2^2+^** and b) **3^2+^** with averaged Ru−N bond lengths along the respective Jahn–Teller axis as simplified reaction coordinate. DFT optimized geometries and spin densities of the ^3^MLCT states of **2^2+^** and b) **3^2+^** (isosurface value at 0.01 a. u.).

The difference between the ^3^MC states of **2^2+^** and **3^2+^** is the location of the Jahn–Teller distortion. In **2^2+^**, a cpmp ligand is necessarily involved with large changes in Ru‐N4 and Ru‐N6 distances and a high torsional distortion involving the CO‐py unit (Figure 6 a). On the other hand, the ddpd ligand in **3^2+^** enables a Jahn–Teller distortion, which is rather symmetrically distributed over the Ru‐N4 and Ru‐N6 bonds (Figure 6 b). The ancillary tridentate ligands are barely distorted relative to the ground‐state geometry. Obviously, the π‐accepting CO‐py unit experiences a stronger distortion than the more electron‐rich NMe‐py units. This might be ascribed to the loss of π‐backbonding in the ^3^MC state due to the electron depletion in the metal π orbitals. For a more detailed view, the geometries of ^1^GS, ^3^MLCT and ^3^MC states were optimized along the Jahn–Teller type axis of the distorted ^3^MC states (projected onto the two averaged elongated Ru−N bond lengths, Figure 7). The potential energy well of the ^3^MC state along the Jahn–Teller mode is steeper for **2^2+^** than for **3^2+^**. The optimized ^3^MC geometries of **3^2+^** with Ru‐N distances of ≈2.3 and 2.6 Å along the Jahn–Teller axis are essentially isoenergetic (Figures 6 b and 7 b). Consequently, the crossing point of the ^3^MC state with the singlet ground state at around 2.6–2.7 Å is higher in energy by ca. 0.2 eV for **2^2+^** than for **3^2+^** with respect to the energy minimum of the respective ^3^MC states. Stronger Ru−N bonds and a more rigid ligand backbone may account for the steeper ^3^MC potential well of **2^2^** ^+^ as compared to **3^2^** ^+^. Consequently, the type of the bridging units between the coordinating pyridines modifies the excited‐state potential steepness, which in turn affects the excited‐state dynamics. Obviously, CO linkers are better than NMe linkers in this respect. This also fits to the excellent performance of [Ru(dcpp)_2_]^2+^, which exclusively possesses CO bridging moieties in the six‐membered chelate rings.^46^

### Reductive quenching of the ^3^MLCT state

The intense absorption band at 549 nm with a molar extinction coefficient of 5200 m ^−1^ cm^−1^, the 477 ns ^3^MLCT state lifetime, the sufficient redox stability and the ^3^MLCT energy of **2^2+^** (1.79 eV) should enable excited‐state quenching by suitable redox partners. The excited‐state redox potential of **2^2+^** *E*_1/2_(*2^2+^/**2^.+^**)=+0.50 V vs. ferrocene is somewhat higher than that of *[Ru(bpy)_3_]^2+^/[Ru(bpy)_3_]^+^ (*E*_1/2_=+0.41 V vs. ferrocene). Furthermore, green light (560 nm) can be used to excite **2^2+^**, while the molar extinction coefficient of [Ru(bpy)_3_]^2+^ at 560 nm (*ϵ*=250 m ^−1^ cm^−1^) is ca. 21 times smaller. Hence, using [Ru(bpy)_3_]^2+^ as photosensitizer typically requires the use of blue light matching its MLCT maximum at 452 nm. Consequently, we were interested in the reductive quenching of the ^3^MLCT state of **2^2+^** using low‐energy green light.

Gratifyingly, aliphatic and aromatic amines such as *N*,*N*‐diisopropylethylamine (DIPEA), *p*‐toluidine and *N*,*N*,*N*’,*N*’‐tetramethyl‐1,4‐phenylendiamine (TMPA) quench the ^3^MLCT emission of **2^2+^**. Stern–Volmer analyses yield the Stern–Volmer constants *K*_SV_=2.2 (DMF, *λ*_exc_=555 nm), 93 (CH_3_CN, *λ*_exc_=546 nm) and 2819 m ^−1^ (DMF, *λ*_exc_=555 nm), respectively (Supporting Information, Figures S52–S54). For the aromatic amines, the higher *K*_SV_ of TMPA correlates to its lower TMPA/TMPA**^.^** ^+^ redox potential (−0.27 V^70^ vs. ferrocene) than that of *p*‐toluidine (+0.34 V vs. ferrocene^71^). With the excited‐state lifetime of 477 ns of **2^2+^**, the quenching rate constants of the amines amount to *k*_q_=0.005×10^9^, 0.19×10^9^ and 5.91×10^9^ m ^−1^ s^−1^ for DIPEA, *p*‐toluidine and TMPA, respectively.

**Scheme 3:**
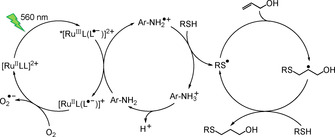
Photocatalytic radical thiol–ene click reaction utilizing the (green) light‐induced reductive quenching pathway of [RuLL]^2+^=**2^2+^**, **3^2+^** and [Ru(bpy)_3_]^2+^ mediated by *p*‐toluidine (Ar=*p*‐C_6_H_4_‐CH_3_) according to ref. 71 (R=CH_2_‐CHNHBoc‐COOCH_3_).

Encouraged by these initial quenching results and the recorded reduction potential of **2^2+^** (*E*_1/2_(**2^2+^**/**2^.+^**)=−1.29 V), which could be sufficient for re‐oxidation by oxygen, a visible light induced radical thiol–ene click reaction^71^, ^72^, ^73^ was attempted using **2[PF_6_]_2_**, **3[PF_6_]_2_** and [Ru(bpy)_3_][PF_6_]_2_ (2.2–2.5 mol‐%) as sensitizer, allyl alcohol (2.5 equiv) as olefin component, *N*‐Boc cysteine methyl ester (1.0 equiv) as thiol component and *p*‐toluidine (0.46 equiv) as redox mediator as developed by Yoon et al. (Scheme 3).^71^ The thiol quenches the emission of **2[PF_6_]_2_** only very moderately (*K*_SV_=0.60 m ^−1^, CH_3_CN, *λ*_exc_=546 nm; *k*_q_=0.0013×10^9^ m ^−1^ s^−1^; (Supporting Information, Figure S55). Consequently, the observed photoreactivity occurs via quenching of the excited ruthenium(II) complex by the amine as suggested by Yoon for [Ru(bpy)_3_]^2+^.^71^ In contrast to the reported conditions, which relied on blue light (450 nm), we employed green light (560±5 nm by bandpass filters; Supporting Information, Figure S56). After 3 h of irradiation, 56, 18 and 20 % of the thiol–ene product were observed for **2[PF_6_]_2_**, **3[PF_6_]_2_** and [Ru(bpy)_3_][PF_6_]_2_, respectively (Supporting Information, Figure S57). The smaller molar extinction coefficient at 560 nm (Figure 4), a lower excited‐state energy (Supporting Information, Figure S51) and a significantly smaller ^3^MLCT lifetime account for the poorer performance of **3[PF_6_]_2_** (vide supra). The lower yields and slow conversion obtained with [Ru(bpy)_3_][PF_6_]_2_ are ascribed to its significantly lower molar extinction coefficient at 560 nm. Hence, with respect to employing green instead of blue light, **2[PF_6_]_2_** outperforms the standard sensitizer [Ru(bpy)_3_][PF_6_]_2_ in this exemplary photosensitized reaction.

## Conclusions

With the aim to lower MLCT and increase ^3^MC energies and possibly to induce further luminescent states of ^3^ILCT or ^3^LL'CT character in ruthenium(II) complexes, we prepared the donor–acceptor ligand cpmp and its homo‐ and heteroleptic ruthenium(II) complexes **2^2+^** and **3^2+^** (with ddpd as ancillary tridentate ligand). The cpmp and ddpd ligands form six‐membered chelate rings which increases the ligand field splitting and hence the ^3^MC energies. The electron accepting py_2_CO and electron donating py_2_NMe units within the same cpmp ligand lead to ILCT/LLCT transitions in the complexes. However, these are found at too high energy to enable emission from these states and to play a significant role in the excited‐state decay when excited at lower energy. The ^3^MLCT states are emissive at room temperature in solution. The homoleptic complex **2^2+^** emits at 709 nm with a luminescence quantum yield of 1.3 % and a 477 ns excited‐state lifetime, while the heteroleptic complex **3^2+^** featuring an additional ddpd ligand with only py_2_NMe units is less emissive (755 nm, 0.037 %, 56 ns). This difference is ascribed to the energy gap law and furthermore to a higher flexibility of the coordinated ddpd ligand in **3^2+^** facilitating non‐radiative decay to the ground state. These results highlight the importance of the excited‐state geometries in addition to the excited‐state energies for the actual excited‐state dynamics. The complexes are active in photoinduced electron transfer reactions with *p*‐toluidine as redox mediator using green light as excitation source. Due to its more favorable optical properties, **2^2+^** is a more potent photosensitizer than **3^2+^**.

## Experimental Section

### General procedures

CH_3_CN and diethyl ether were distilled under argon atmosphere from CaH_2_ and sodium, respectively. All other solvents and reagents were used as received from commercial suppliers (Acros, Alfa Aesar, Fischer and Sigma–Aldrich). Microwave heating was performed in a Discover Benchmate Plus (CEM Synthesis) single‐mode microwave cavity, producing continuous irradiation at 2.455 GHz with 150 W. Reaction mixtures were stirred with a magnetic stir bar during irradiation. Temperature and irradiation power were monitored during the course of the reaction. NMR spectra were recorded on a Bruker Avance DRX 400 spectrometer at 400.31 MHz (^1^H), 100.05 MHz (^13^C{^1^H}), and 162.05 MHz (^31^P{^1^H}). All resonances are reported in ppm versus the solvent signal as an internal standard [CD_3_CN (^1^H, *δ*=1.94; ^13^C, *δ*=1.24 ppm)]^74^ or versus external H_3_PO_4_ (85 %) (^31^P: *δ*=0 ppm); (s)=singlet, (d)=doublet, (t)=triplet, (sept)=septet, (m)=multiplet. Atom numbering is shown in the Supporting Information at the respective NMR spectra. IR spectra were recorded with a Bruker ALPHA II FT‐IR spectrometer with a platinum Di‐ATR module (cpmp) and BioRad Excalibur FTS 3100 spectrometer using KBr disks (**1[PF_6_]**, **2[PF_6_]_2_**, **3[PF_6_]_2_**). Electrochemical experiments were carried out on a BioLogic SP‐50 voltammetric analyzer using platinum wires as counter and working electrodes and 0.01 m Ag/AgNO_3_ as the reference electrode. The measurements were carried out at a scan rate of 100 mV s^−1^ for cyclic voltammetry experiments and at 50 mV s^−1^ for square‐wave voltammetry experiments using 0.1 m [*n*Bu_4_N][PF_6_] as the supporting electrolyte in CH_3_CN. Potentials are referenced to the ferrocene/ferrocenium couple. UV/Vis/NIR spectroelectrochemical experiments were performed using a BioLogic SP‐50 voltammetric analyzer and a Specac omni‐cell liquid transmission cell with CaF_2_ windows equipped with a Pt‐gauze working electrode, a Pt‐gauze counter electrode and a Ag wire as pseudo reference electrode, melt‐sealed in a polyethylene spacer (approximate path length 1 mm) in CH_3_CN (0.3 mm and 0.9 mm for oxidation and reduction, respectively) containing 0.1 m [*n*Bu_4_N][PF_6_].^75^ IR spectroelectrochemical experiments were performed using a BioLogic SP‐200 voltammetric analyzer and using the same cell, electrodes and electrolyte as above (12 mm). UV/Vis/NIR spectra were recorded on a Varian Cary 5000 spectrometer using 1.00 cm cells. Emission spectra were recorded on a Varian Cary Eclipse spectrometer. For low temperature photoluminescence measurements, a solution of the complex in butyronitrile freshly filtered over alumina was filled into a quartz cuvette in an argon filled glovebox and the cuvette was sealed and transferred to an Oxford cryostate (Oxford instruments Optistat^DN^). Measurements were conducted at 295 K and 77 K. Fluorescence decays were recorded with an Edinburgh Instruments lifetime spectrometer (FLS 920) equipped with a microchannel plate photomultiplier tube (MCP‐PMT; Hamamatsu R3809U‐50) using a 330 nm ps laser diode (EPLED, Edinburgh Instruments) as excitation light source. All measurements were performed under magic‐angle conditions (polarizers in the excitation and emission channel set to 0° and 54.7°, respectively). Transient absorption spectra were recorded with a time resolution of ca. 100 fs by a pump‐probe setup based on a Ti:sapphire laser system (CPA 2001, Clark MXR, Inc.) operating at a center wavelength of 775 nm and at a repetition rate of 1 kHz. Applying a noncollinear optical parametric amplifier (NOPA), excitation pulses with a center wavelength of 540 nm were generated whose dispersion was controlled by a prism compressor. As probe, a white light continuum was used which was generated in a CaF_2_ crystal. The polarizations of pump and probe were set to magic angle and the beams were focused into the sample to overlapping spots with diameters of 330 μm for the pump and 90 μm for the probe. After the sample, the probe was dispersed by a prism and transmission changes were spectrally resolved recorded by a CCD array detector. The sample compounds were dissolved in CH_3_CN and the solutions were filled into a 1 mm thick fused silica cuvette. Time‐resolved luminescence was measured by a streak camera system (C10627, Hamamatsu Photonics) using again excitation pulses at 540 nm generated by a NOPA. For the measurements with the streak camera, the samples dissolved in CH_3_CN were filled into a 1 cm thick fused silica cuvette. All samples were deaerated with argon prior the spectroscopic measurements. The luminescence quantum yield of **2[PF_6_]_2_** was determined with an Ulbricht integrating sphere (Quantaurus‐QY C11347‐11, Hamamatsu).^76^, ^77^ The luminescence quantum yield of **3[PF_6_]_2_** was determined by comparing the areas under the emission spectra on an energy scale cm^−1^ recorded for optically matched solutions of the sample and the reference {*Φ*(**2[PF_6_]_2_**)=0.0088 in aerated CH_3_CN}. ESI mass spectra were recorded on an Agilent 6545 QTOF‐MS spectrometer (cpmp) and a Micromass Q‐TOF‐Ultima spectrometer (**1[PF_6_]**, **2[PF_6_]_2_**, **3[PF_6_]_2_**). Elemental analyses were performed by the microanalytical laboratory of the department of chemistry of the University of Mainz. The catalysis experiments were carried out using an Asahi Spectra Max‐303 Xenon Light Source (300 W), together with 560 nm±5 nm filters (Supporting Information, Figure S56).

### Crystal structure determination

Intensity data were collected with a Bruker AXS Smart1000 CCD diffractometer with an APEX II detector and an Oxford cooling system and corrected for absorption and other effects using Mo_Kα_ radiation (*λ*=0.71073 Å). The diffraction frames were integrated using the Bruker SMART software package,^78^ and most were corrected for absorption with MULABS79a of the PLATON software package.79b The structures were solved by direct methods and refined by the full‐matrix method based on *F* ^2^ using the SHELX software package^80^ using the ShelXle graphical interface.^81^ Non‐hydrogen atoms were refined anisotropically, while the positions of all hydrogen atoms were generated with appropriate geometric constraints and allowed to ride on their respective parent carbon atoms with fixed isotropic thermal parameters. CCDC 1852838 (**3[PF_6_]_2_**) contains the supplementary crystallographic data for this paper. These data are provided free of charge by The Cambridge Crystallographic Data Centre.

### Density functional theoretical calculations

DFT calculations were carried out using the ORCA program package (version 4.0.1).^82^, ^83^ All calculations were performed using the B3LYP functional^84^, ^85^, ^86^ and employ the RIJCOSX approximation.^87^, ^88^ Relativistic effects were calculated at the zeroth order regular approximation (ZORA) level.^89^ The ZORA keyword automatically invokes relativistically adjusted basis sets. To account for solvent effects, a conductor‐like screening model (CPCM) modeling acetonitrile was used in all calculations.^90^, ^91^ Geometry optimizations were performed using Ahlrichs polarized valence triple‐zeta basis set (old‐TZVP equivalent to def2‐TZVP from ORCA 3.0) together with a segmented all electron relativistically contracted (SARC) auxiliary basis set.^89^, ^92^ Atom‐pairwise dispersion correction was performed with the Becke–Johnson damping scheme (D3BJ).^93^, ^94^ The presence of energy minima was checked by numerical frequency calculations. Explicit counterions and/or solvent molecules were not taken into account. The ^3^MC structures were found by constraining certain Ru−N bonds to longer distances and re‐optimizing the thus obtained structure without geometry constraints. All optimized geometries were ascertained as minima by numerical frequency analysis. For the potential diagrams, the two Ru−N bonds that are elongated in the ^3^MC states were constrained to the respective averaged elongated values. The assignment of the state characters has been done dividing the molecule into three fragments (metal center and two ligands) and calculating charge transfer numbers, as implemented in the TheoDore software package.^95^, ^96^

### Synthesis of cpmp (6,2’’‐carboxypyridyl‐2,2’‐methylamine‐pyridyl‐pyridine)

6‐Bromo‐*N*‐methyl‐*N*‐(pyridin‐2‐yl)pyridyl‐2‐amine^57^ (3.06 g, 16 mmol, 1.0 equiv.) was dissolved in dry diethyl ether (100 mL) under argon. At −78 °C, *n*‐butyl lithium (2.5 m in hexane, 7.0 mL, 18 mmol, 1.1 equiv.) was added dropwise. After stirring for 1 h at −78 °C, 2‐cyanopyridine (1.67 g, 16 mmol, 1.0 equiv.) dissolved in dry diethyl ether (20 mL) was added dropwise, keeping the temperature below −70 °C. After 30 minutes stirring below −70 °C, the solution was allowed to warm to room temperature. The dark brown solution was poured over ice (500 g) to yield an orange solution, which was acidified with hydrochloric acid (1 m, 150 mL). The separated organic layer was extracted with hydrochloric acid (1 m, 3×150 mL). The combined aqueous phases were neutralized with saturated aqueous sodium carbonate solution. The aqueous solution was extracted with methylene chloride (3×150 mL). The combined organic phases were dried over magnesium sulfate and the solvent was removed under reduced pressure. The crude product was purified by column chromatography (silica gel, petroleum ether/ethyl acetate 1:1) to yield a yellow oil (25 %, 1.29 g, 4.44 mmol). *R*_f_ (SiO_2_, petroleum ether/ethyl acetate 1:1)=0.30. Elem. anal. calcd for C_17_H_14_N_4_O *x* 0.1 H_2_O: C, 69.90; H, 4.90; N, 19.18. Found: C, 69.91; H, 5.17; N, 19.08. ^1^H NMR (CD_3_CN): *δ*=8.70 (dt, *J=*4.8, 1.4 Hz, 1 H, H^16^), 8.30–8.24 (m, 2 H, H^1^), 7.94–7.89 (m, 2 H, H^14^, H^15^), 7.79 (dd, *J=*8.5, 7.4 Hz, 1 H, H^9^), 7.58–7.50 (m, 3 H, H^3^, H^10^, H^17^), 7.39 (d, *J=*8.5 Hz, 1 H, H^8^), 7.32 (d, *J=*8.4 Hz, 1 H, H^4^), 6.91 (ddd, *J=*7.4, 5.1, 0.9 Hz, 1 H, H^2^), 3.48 (s, 3 H, H^6^). ^13^C{^1^H} NMR (CD_3_CN): *δ*=192.8 (C^12^), 156.2 (C^5^), 155.7 (C^7^), 154.3 (C^13^), 151.7 (C^11^), 148.1 (C^16^), 146.9 (C^1^), 137.1 (C^9^), 136.4 (C^10^), 135.8 (C^14^), 125.3 (C^17^), 123.5 (C^15^), 116.8 (C^3^), 116.2 (C^8^), 115.6 (C^2^), 114.5 (C^4^), 34.5 (C^6^). MS (ESI^+^): *m*/*z=*291.1 (100 %, [*M*+H]^+^). MS(HR‐ESI^+^): calcd for C_17_H_15_N_4_O^+^: *m*/*z=*291.1240. Found: *m*/*z=*291.1234. IR (ATR): $\tilde{\nu }$ =3052 (vw, CH), 3003 (vw, CH), 2958 (vw, CH), 2910 (vw, CH), 2816 (vw, CH), 1679 (m, C=O), 1578 (s), 1565 (m), 1429 (vs.), 1348 (s), 1320 (m), 1267 (m), 1241 (w), 1185 (vw), 1141 (w), 1115 (w), 1081 (w), 1046 (vw), 973 (m), 891 (w), 826 (w), 776 (m), 737 (s), 698 (m), 655 (w), 614 (m), 559 (vw), 432 (vw), 403 (w) cm^−1^. CV (CH_3_CN, vs. FcH/FcH^+^): *E*_1/2_=−1.99 (irrev.), +0.97 (irrev.) V. UV/Vis (CH_3_CN): *λ*_max_ (*ϵ*)=231 (13000), 267 (9790), 305 (12 800), 354 nm (sh, 1970 m ^−1^ cm^−1^). Emission (CH_3_CN, 295 K, *λ*_exc_=354 nm): *λ*_em_=455 nm.

### Synthesis of 1[PF_6_] ([RuCl(cpmp)(NCCH_3_)]_*n*_[PF_6_]_*n*_; *n*=2)

Di‐*μ*‐chlorido‐bis[chlorido(*η* ^6^‐1‐isopropyl‐4‐methylbenzene)ruthenium(II)]^58^ (1.06 g, 1.72 mmol, 1.0 equiv.), cpmp (1.00 g, 3.45 mmol, 2.0 equiv.) and KPF_6_ (1.06 g, 5.74 mmol, 3.3 equiv.) were dissolved in acetonitrile (150 mL). The solution was heated to reflux for 6 h to yield a red‐purple solution. After filtration over alumina, the solvent was removed under reduced pressure. The solid was dissolved in acetonitrile (5 mL) and precipitated by addition of diethyl ether (100 mL). The red‐purple solid was washed with diethyl ether (250 mL). The red‐purple micro crystals were dried under reduced pressure. Yield 1.99 g (3.25 mmol, 94 %). ^1^H NMR (CD_3_CN) *δ*=8.89 (d, *J=*5.3 Hz, 1 H, H^17^), 8.85 (dd, *J=*5.9, 1.9 Hz, 1 H, H^1^), 8.19 (dd, *J=*7.0, 1.0 Hz, 1 H, H^14^), 8.11 (dd, *J=*7.8, 1.3 Hz, 1 H, H^15^), 8.00 (dd, *J=*7.4, 7.4 Hz, 1 H, H^9^), 7.94 (ddd, *J=*8.8, 7.4, 1.8 Hz, 1 H, H^3^), 7.87 (dd, *J=*7.3 Hz, 1 H, H^10^), 7.62 (ddd, *J=*7.4, 5.7, 1.7 Hz, 1 H, H^16^), 7.51 (dd, *J=*8.3, 1.3 Hz, 1 H, H^8^), 7.38 (dd, *J=*8.3, 1.0 Hz, 1 H, H^2^), 7.21 (ddd, *J=*7.2, 5.8, 1.2 Hz, 1 H, H^4^), 3.63 (s, 3 H, H^6^), 2.59 (s, 3 H, H^19^). ^13^C{^1^H} NMR (CD_3_CN): *δ*=182.4 (C^12^), 157.6 (C^7^), 156.7 (C^5^), 156.6 (C^13^), 155.6 (C^11^), 155.1 (C^17^), 153.9 (C^1^), 137.8 (C^3^), 137.1 (C^9^), 136.5 (C^15^), 126.1 (C^14^), 126.0 (C^16^), 125.6 (C^18^), 122.7 (C^10^), 118.7 (C^4^), 116.8 (C^8^), 113.0 (C^2^), 38.9 (C^6^), 2.9 (C^19^). ^31^P{^1^H} NMR (CD_3_CN): *δ*=−144.6 (sept, ^1^ *J*_PF_=700 Hz). MS (ESI^+^): *m*/*z=*426.9 (63 %, [RuCl(cpmp)]^+^), 467.9 (100 %, [RuCl(cpmp)(NCCH_3_)]^+^), 485.9 (34 %, [RuCl(cpmp)(H_2_O)(NCCH_3_)]^+^), 508.96 (24 %, [RuCl(cpmp)(NCCH_3_)_2_]^+^). MS (HR‐ESI^+^): calcd for C_21_H_20_ClRuN_6_O^+^: *m*/*z=*509.0425. Found: *m*/*z=*509.0421. IR (KBr): $\tilde{\nu }$ =3091 (vw, CH), 2966 (vw, CH), 2933 (vw, CH), 2275 (vw, C≡N), 1672 (s, C=O), 1590 (s), 1483 (s), 1455 (s), 1437 (s) 1353 (s), 1314 (w), 1290 (vw), 1266 (w), 1247 (m), 1172 (vw), 1151 (w), 1091 (vw), 1063 (vw), 1030 (w), 972 (w), 843 (vs., PF), 757 (s), 717 (w), 704 (w), 675 (vw), 582 (w), 559 (vs., PF_2_, def), 519 (vw) cm^−1^. UV/Vis (CH_3_CN): *λ*_max_ (*ϵ*)=276 (19 100), 341 (10 500), 494 nm (3150 m ^−1^ cm^−1^). [RuCl(cpmp)(NCCH_3_)][PF_6_] was used directly without further purification.

### Synthesis of 2[PF_6_]_2_ ([Ru(cpmp)_2_][PF_6_]_2_)

[RuCl(cpmp)(NCCH_3_)][PF_6_] (**1[PF_6_]**) (100 mg, 0.16 mmol, 1.0 equiv.) and cpmp (77 mg, 0.27 mmol, 1.6 equiv.) were dissolved in a mixture of acetonitrile (1 mL) and ethanol (6 mL). The solution was heated to reflux in a laboratory microwave oven for 20 h (100 W, 90 °C). The solvents were removed under reduced pressure. The solid was dissolved in acetonitrile (1 mL) and precipitated by addition of diethyl ether (100 mL). The precipitate was washed with diethyl ether (100 mL) to remove excess ligand. The dark red product was dissolved in acetonitrile (1 mL). Addition of an aqueous solution of [NH_4_][PF_6_] (1 g, 6 mmol, 37 equiv. in 100 mL H_2_O) resulted in the precipitation of a purple solid, which was collected by filtration and purified by diffusion of diethyl ether into a solution of **2[PF_6_]_2_** in acetonitrile. The purple crystals were dried under reduced pressure. Yield: 76 mg (0.08 mmol, 48 %). ^1^H NMR (CD_3_CN): *δ*=8.21 (dd, ^3^ *J*=8.0, 8.0 Hz, 2 H, H^9^), 8.11–8.01 (m, 4 H, H^16^, H^17^), 7.92 (d, ^3^ *J*=7.8 Hz, 2 H, H^10^), 7.88 (ddd, ^3^ *J*=7.9, 7.9 Hz, ^4^ *J*=1.8 Hz, 2 H, H^3^), 7.61 (d, ^3^ *J*=8.4 Hz, 2 H, H^8^), 7.57 (d, ^3^ *J*=5.7 Hz, 2 H, H^14^), 7.35 (ddd, ^3^ *J*=7.6 Hz, ^3^ *J*=5.7 Hz, ^4^ *J*=2.3 Hz, 2 H, H^15^), 7.21 (d, ^3^ *J*=8.6 Hz, 2 H, H^4^), 7.03 (dd, ^3^ *J*=5.9 Hz, ^4^ *J*=1.8 Hz, 2 H, H^1^), 6.80 (dd, ^3^ *J*=6.6 Hz, ^3^ *J*=6.6 Hz, 2 H, H^2^), 2.88 (s, 6 H, H^6^). ^13^C{^1^H} NMR (CD_3_CN): *δ*=183.3 (C^12^), 156.6 (C^5^), 155.8 (C^7^), 154.5 (C^14^), 153.7 (C^11^), 153.6 (C^13^), 152.2 (C^1^), 139.6 (C^9^), 139.0 (C^3^), 137.7 (C^16^), 127.7 (C^15^), 126.8 (C^17^), 123.8 (C^10^), 120.4 (C^2^), 119.2 (C^8^),113.8 (C^4^), 38.2 (C^6^). ^31^P{^1^H} NMR (CD_3_CN): *δ*=−145.5 (sept, ^1^ *J*_PF_=700 Hz). MS (ESI^+^) *m*/*z=*341.1 (64 %, [Ru(cpmp)_2_]^2+^), 827.1 (100 %, [Ru(cpmp)_2_][PF_6_]^+^). MS (HR‐ESI^+^): calcd for C_34_H_28_F_6_RuN_8_O_2_P^+^: *m*/*z=*827.1021. Found: *m*/*z=*827.1003. Elem. anal. calcd for C_34_H_28_F_12_RuN_8_O_2_P_2_: C, 42.03; H, 2.90; N, 11.53. Found: C, 41.88; H, 3.04; N, 11.86. IR (ATR): $\tilde{\nu }$ =3110 (vw, CH), 3092 (vw, CH), 1664 (w, CO), 1587 (w), 1486 (vw), 1455 (w), 1437 (w), 1354 (w), 1318 (vw), 1292 (vw), 1265 (vw), 1244 (vw), 1176 (vw), 1149 (w), 1141 (vw), 1125 (vw), 1105 (vw), 1089 (vw), 1064 (vw), 1021 (vw), 973 (vw), 871 (vw), 833 (vs., PF), 794 (w), 777 (m), 758 (m), 710 (w), 668 (vw), 651 (vw), 634 (vw), 612 (vw), 578 (vw), 555 (vs., PF_2_), 515 (vw) cm^−1^. IR (KBr): $\tilde{\nu }$ =3127 (vw, CH), 3103 (vw, CH), 3040 (vw, CH), 3015 (v), 2985 (vw), 2916 (vw), 2835 (vw), 2249 (vw), 1666 (vs., CO), 1591 (s), 1572 (w), 1480 (s), 1469 (m), 1411 (w), 1379 (w), 1352 (s), 1314 (m), 1291 (w), 1251 (m), 1194 (vw), 1174 (w), 1148 (w), 1137 (w), 1118 (vw), 1100 (vw), 1087 (vw), 1064 (vw), 1025 (w), 972 (w), 921 (vw), 879 (s), 846 (vs., PF), 793 (m), 757 (s), 714 (w), 675 (vw), 653 (vw), 633 (vw), 615 (vw), 577 (w), 558 (vs., PF_2_), 519 (vw), 469 (vw), 449 (vw), 430 (vw), 414 (vw), 398 (vw) cm^−1^. CV (CH_3_CN, vs. FcH/FcH^+^): *E*_1/2_=−2.27 (irrev.), −2.12 (irrev.), −1.46 (rev.).‐1.29 (rev.), +0.92 (rev.) V. UV/Vis (CH_3_CN): *λ*_max_ (*ϵ*)=269 (40 800), 342 (20 600), 549 nm (5200 m ^−1^ cm^−1^). Emission (CH_3_CN, 298 K): *λ*_em_=709 nm (*λ*_exc_=554 nm), *τ*=477 ns (deaerated), 237 ns (aerated) (*λ*_exc_=544 nm), *Φ* (*λ*_exc_=544 nm)=1.3 % (deaerated), 0.88 % (aerated). Emission (^*n*^PrCN, 77 K, *λ*_exc_=549 nm): *λ*_em_=693 nm.

### Synthesis of 3[PF_6_]_2_ ([Ru(cpmp)(ddpd)][PF_6_]_2_)

[RuCl(cpmp)(NCCH_3_)]PF_6_ (**1[PF_6_]**) (100 mg, 0.16 mmol, 1.0 equiv.) and ddpd^84^ (71 mg, 0.25 mmol, 1.5 equiv.) were dissolved in a mixture of acetonitrile (1 mL) and ethanol (6 mL). The solution was heated to reflux in a laboratory microwave oven for 8 h (100 W, 90 °C). The solvents were removed under reduced pressure. The dark red solid was dissolved in acetonitrile (1 mL) and precipitated by addition of diethyl ether (100 mL). The precipitate was washed with diethyl ether (100 mL) to remove excess ligand. The product was collected by filtration and dissolved in acetonitrile (1 mL). Addition of an aqueous solution of [NH_4_][PF_6_] (1 mg, 6 mmol, 37 equiv. in 100 mL of H_2_O) resulted in the precipitation of a purple solid, which was collected by filtration and purified by slow diffusion of diethyl ether into a solution of **3[PF_6_]_2_** in acetonitrile to yield crystals suitable for single‐crystal X‐ray diffraction. The purple crystals were dried under reduced pressure. Yield: 98 mg (0.10 mmol, 62 %). ^1^H NMR (CD_3_CN): *δ*=8.12 (dd, ^3^ *J*=8.0, 8.0 Hz, 1 H), 8.03 (dd, ^3^ *J*=8.1, 8.1 Hz, 1 H), 8.01–7.97 (m, 2 H), 7.87–7.79 (m, 4 H), 7.55 (dd, ^3^ *J*=8.4 Hz, ^4^ *J*=1.2 Hz, 1 H), 7.42 (d, ^3^ *J*=5.8 Hz, 1 H), 7.30–7.20 (m, 6 H), 7.18 (dd, ^3^ *J*=8.1, 8.1 Hz, 2 H), 6.92–6.84 (m, 2 H), 6.81 (ddd, ^3^ *J*=6.6, 5.9 Hz, ^4^ *J*=1.2 Hz, 1 H), 6.76 (ddd, ^3^ *J*=6.6, 5.9 Hz, ^4^ *J*=1.2 Hz,1 H), 3.02 (s, 3 H, CH_3_), 3.01 (s, 3 H, CH_3_), 2.81 (s, 3 H, CH_3_). ^13^C{^1^H} NMR (CD_3_CN): *δ*=185.0 (C^12^), 159.8, 158.9, 158.3, 157.9, 156.9, 156.3, 156.2, 155.9, 154.4, 154.1, 153.5, 152.7, 141.2, 140.6, 140.2, 140.0, 139.7, 138.7, 128.8, 128.4, 125.4, 121.9, 121.7, 121.4, 119.9, 115.7, 115.6, 115.2, 113.7, 113.4, 40.8, 40.7, 40.0. The nuclei of the two ligands feature too similar chemical shifts to allow a detailed assignment. However, the number of ^1^H and ^13^C NMR resonances, their intensity and multiplicity fit to the proposed structure. ^31^P{^1^H} NMR (CD_3_CN): *δ*=−144.7 (sept, ^1^ *J*_PF_=700 Hz). MS (ESI^+^): *m*/*z=*341.6 (48 %, [Ru(cpmp)(ddpd)]^2+^), 828.2 (100 %, [Ru(cpmp)(ddpd)][PF_6_]^+^). MS (HR‐ESI^+^): calcd for C_34_H_31_F_6_RuN_9_OP^+^: *m*/*z=*828.1337. Found: 828.1320. Elem. anal. calcd for C_34_H_31_F_12_RuN_9_OP_2_: C, 41.98; H, 3.21; N, 12.96. Found: C, 41.72; H, 3.34; N, 12.84. IR (ATR): $\tilde{\nu }$ =3096 (vw, CH), 2976 (vw, CH), 2905 (vw, CH), 2829 (vw, CH), 1666 (vw, CO), 1592 (vw), 1576 (vw), 1486 (w), 1450 (w), 1434 (w), 1351 (vw), 1335 (vw), 1314 (vw), 1246 (vw), 1236 (vw), 1196 (vw), 1170 (vw), 1136 (vw), 1096 (vw), 1064 (vw), 1024 (vw), 972 (vw), 947 (vw), 877 (vw), 829 (vs., PF), 774 (m), 746 (m), 713 (w), 673 (vw), 647 (vw), 633 (vw), 589 (vw), 581 (vw), 555 (s, PF_2_), 521 (vw), 467 (vw), 448 (vw), 437 (vw), 412 (vw) cm^−1^. IR (KBr): $\tilde{\nu }$ =3111 (vw, CH), 2970 (vw, CH), 2913 (vw, CH), 2833 (vw, CH), 1668 (w, CO), 1597 (w), 1578 (w), 1489 (m), 1451 (m), 1436 (m), 1352 (w), 1337 (w), 1316 (vw), 1249 (w), 1171 (vw), 1140 (w), 1097 (vw), 1082 (vw), 1065 (vw), 1024 (vw), 973 (vw), 950 (vw), 842 (vs., PF), 799 (w), 778 (w), 753 (w), 715 (vw), 673 (vw), 664 (vw), 649 (vw), 633 (vw), 611 (vw), 591 (vw), 581 (vw), 557 (s, PF_2_), 528 (vw), 514 (vw), 496 (vw), 494 (vw), 463 (vw), 449 (vw), 438 (vw), 420 (vw), 410 (vw) cm^−1^. CV (CH_3_CN, vs. FcH/FcH^+^): *E*_1/2_=−2.06 (rev.), −1.37 (rev.), +0.67 (rev.) V. UV/Vis (CH_3_CN): *λ*_max_ (*ϵ*)=267 (33 200), 350 (14 200), 541 nm (2800 m ^−1^ cm^−1^). Emission (CH_3_CN, 298 K): *λ*_em_=755 nm (*λ*_exc_=544 nm), *τ*=56 ns (*λ*_exc_=540 nm, streak camera), *Φ* (*λ*_exc_=545 nm, comparative method)=0.04 % (deaerated), 0.028 % (aerated). Emission (^*n*^PrCN, 77 K, *λ*_exc_=541 nm): *λ*_em_=740 nm.

### Reduction of 2[PF_6_]_2_ and 3[PF_6_]_2_ using decamethylcobaltocene

The respective ruthenium(II) complex **2[PF_6_]_2_** or **3[PF_6_]_2_** (0.80 mg) was dissolved in CH_3_CN (0.5 mL). CoCp*_2_ was added (0.27 mg) and the IR spectrum was recorded immediately.

### Catalysis experiments

To an oven‐dried NMR tube were added the olefin (0.157 mmol; dried over molecular sieves), the thiol (0.063 mmol), the sensitizer (**2[PF_6_]_2_**: 1.4 μmol, 2.2 mol‐%; **3[PF_6_]_2_**: 1.4 μmol, 2.2 mol‐%; [Ru(bpy)_3_][PF_6_]_2_: 1.6 μmol, 2.5 mol‐%), *p*‐toluidine (0.029 mmol) and CD_3_CN (0.5 mL). The tube was sealed with a Teflon cap and irradiated with the green light source. Yields were determined by ^1^H NMR spectroscopy using 1,4‐bis(trimethylsilyl)benzene as internal standard.

## Conflict of interest

The authors declare no conflict of interest.

## Supporting information

As a service to our authors and readers, this journal provides supporting information supplied by the authors. Such materials are peer reviewed and may be re‐organized for online delivery, but are not copy‐edited or typeset. Technical support issues arising from supporting information (other than missing files) should be addressed to the authors.

Supplementary
