# Supplementary material for: Green‐Light Activation of Push–Pull Ruthenium(II) Complexes
Source: Chemistry. 2020 Apr 30;26(30):6820–32. doi: 10.1002/chem.202000871 (PMC7318647; doi:10.1002/chem.202000871)
Supplement: Supplementary file 1 — Supplementary [file CHEM-26-6820-s001.pdf]

Chemistry–A European Journal

Supporting Information

Green-Light Activation of Push–Pull Ruthenium(II) Complexes

Johannnes Moll,[a] Cui Wang,[b, c] Ayla Päpcke,[d] Christoph Förster,[a] Ute Resch-Genger,[b] Stefan Lochbrunner,[d] and Katja Heinze*[a]

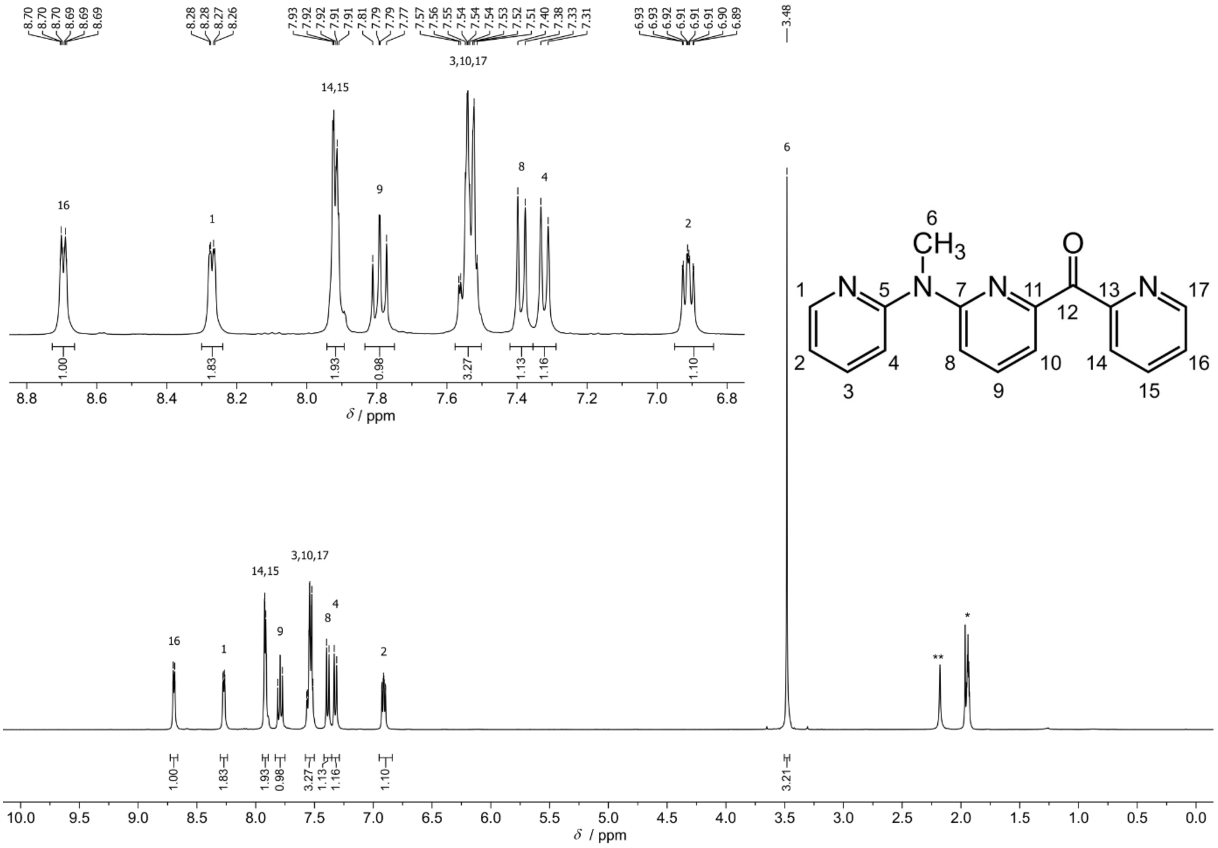

Figure S1. ^{1}H NMR spectrum of cpmp in CD_3CN at 293 K. * denotes solvent resonance; ** denotes water resonance.

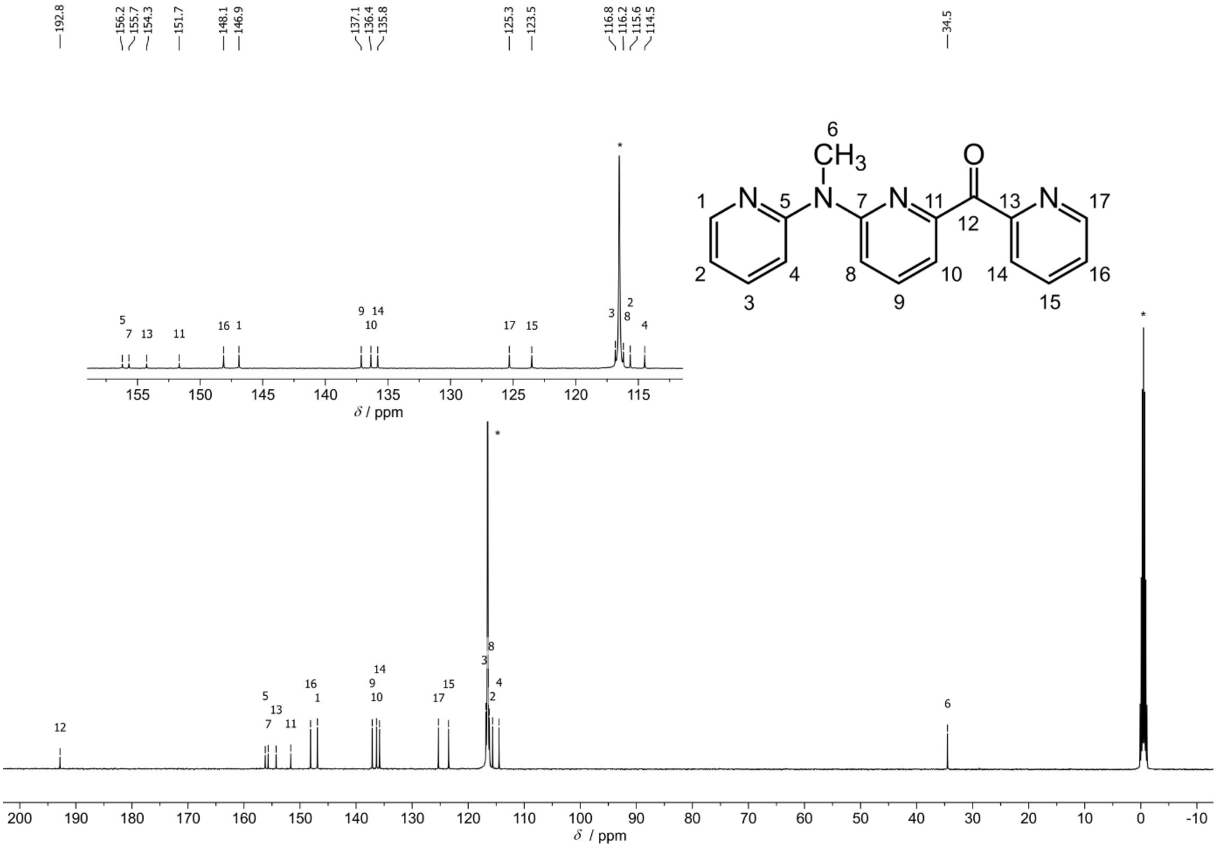

Figure S2. $^{13}C\{^{1}H\}$ NMR spectrum of cpmp in CD_3CN at 293 K. * denote solvent resonances.

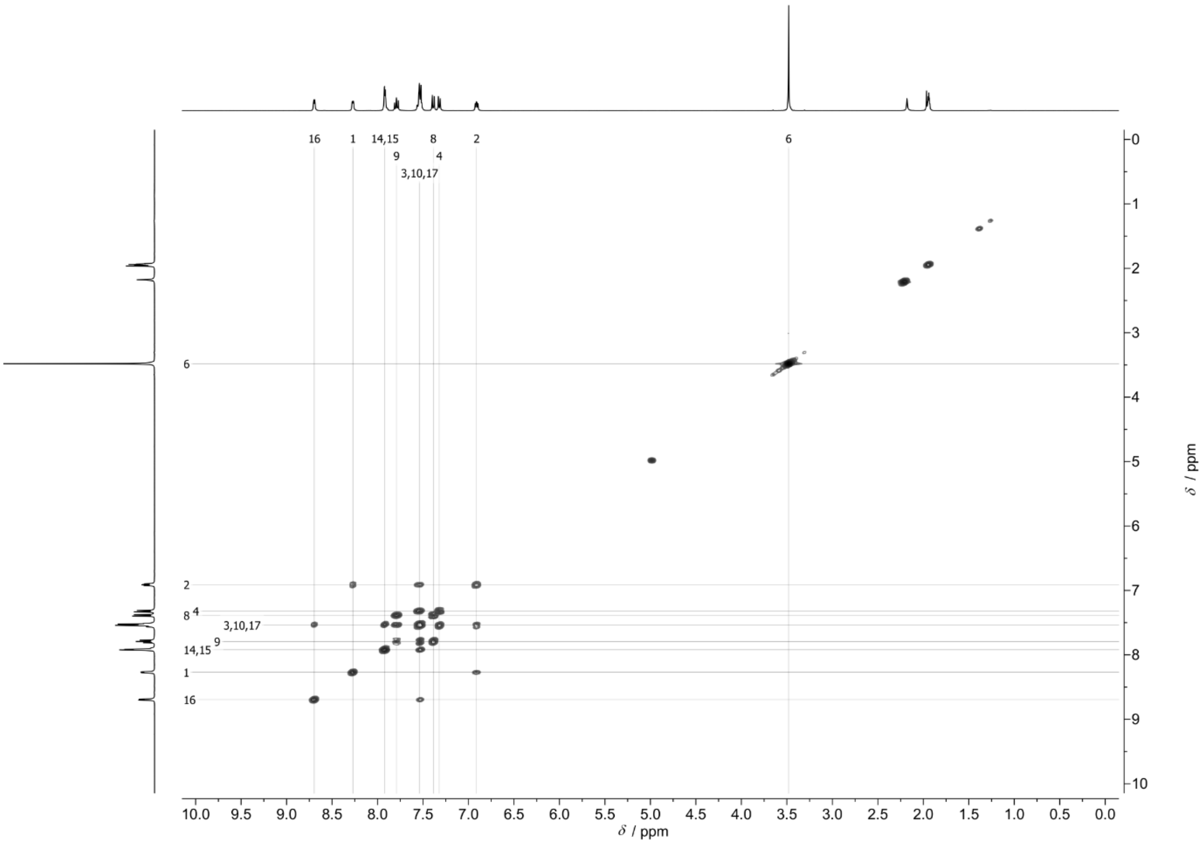

Figure S3. ^{1}H-^{1}H COSY of cpmp in CD_3CN at 293 K.

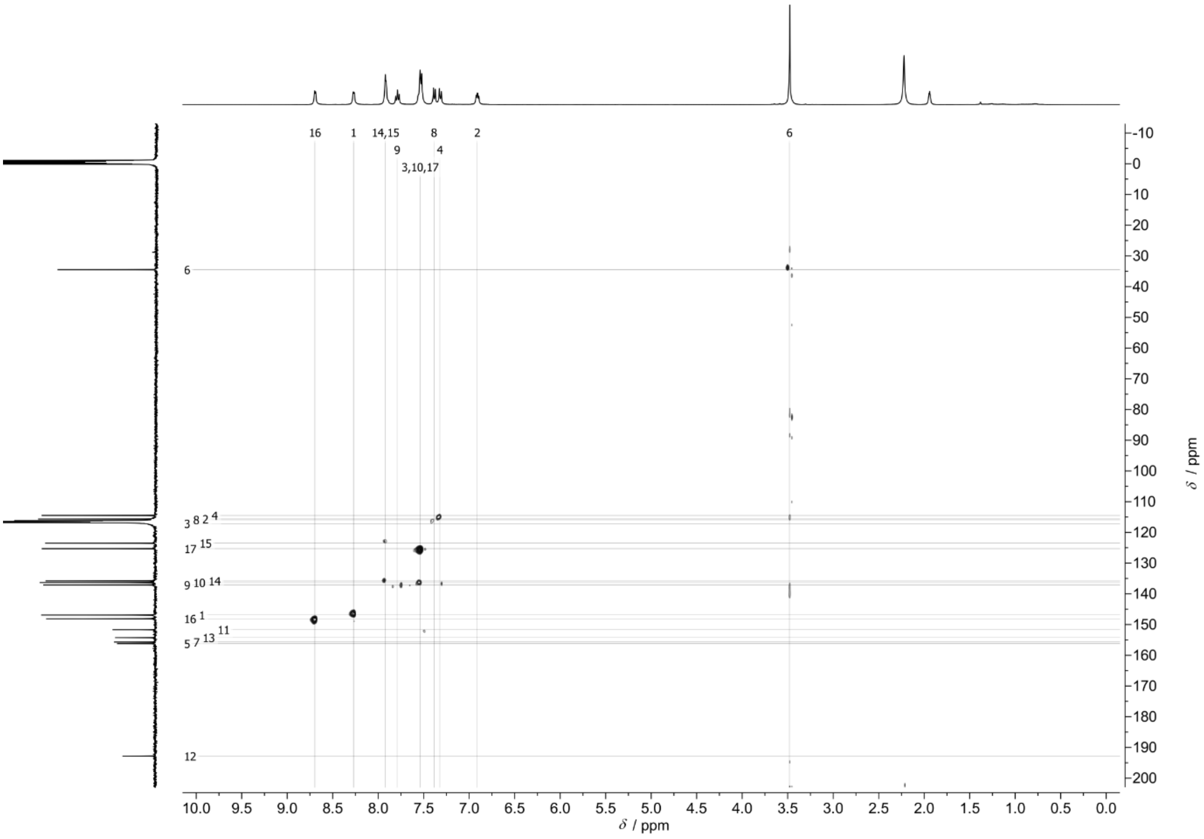

Figure S4. ^{1}H-^{13}C HSQC of cpmp in CD_3CN at 293 K.

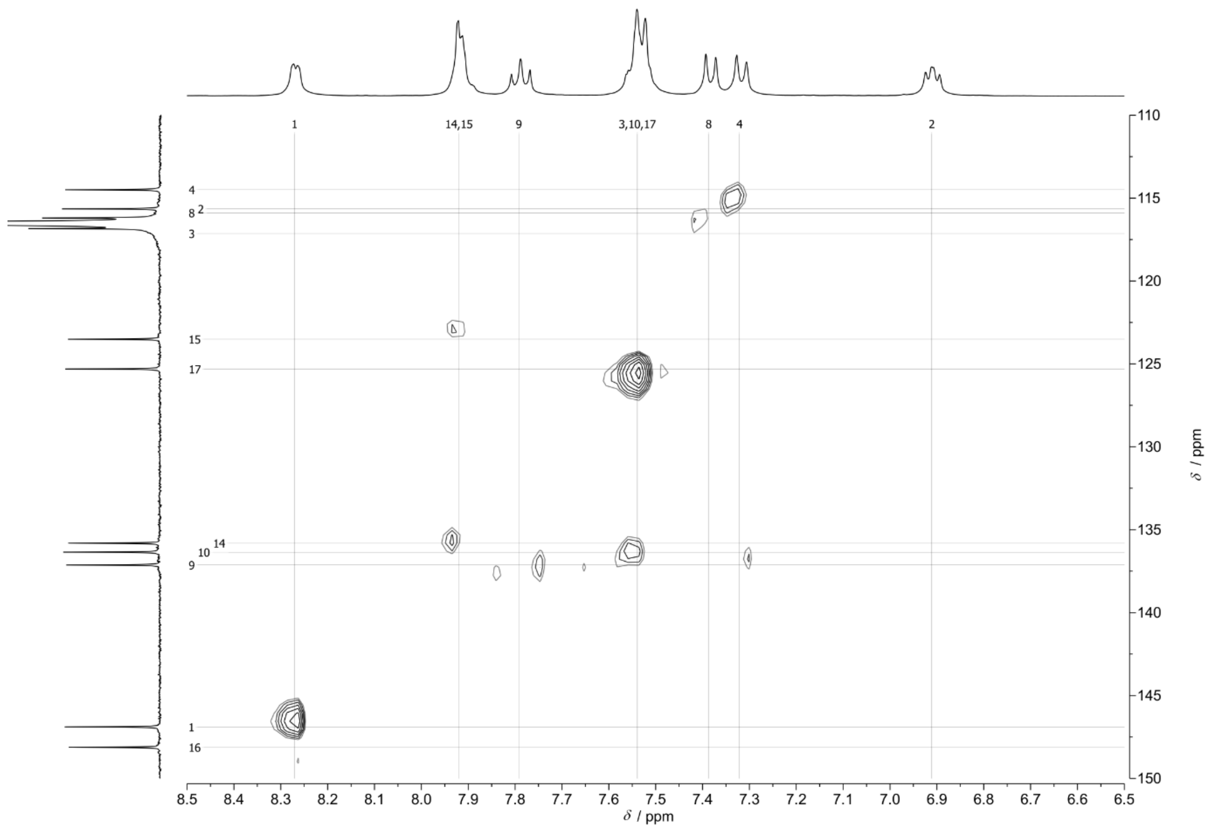

Figure S5. ^{1}H-^{13}C HSQC of cpmp in CD_3CN at 293 K (zoom into aromatic region).

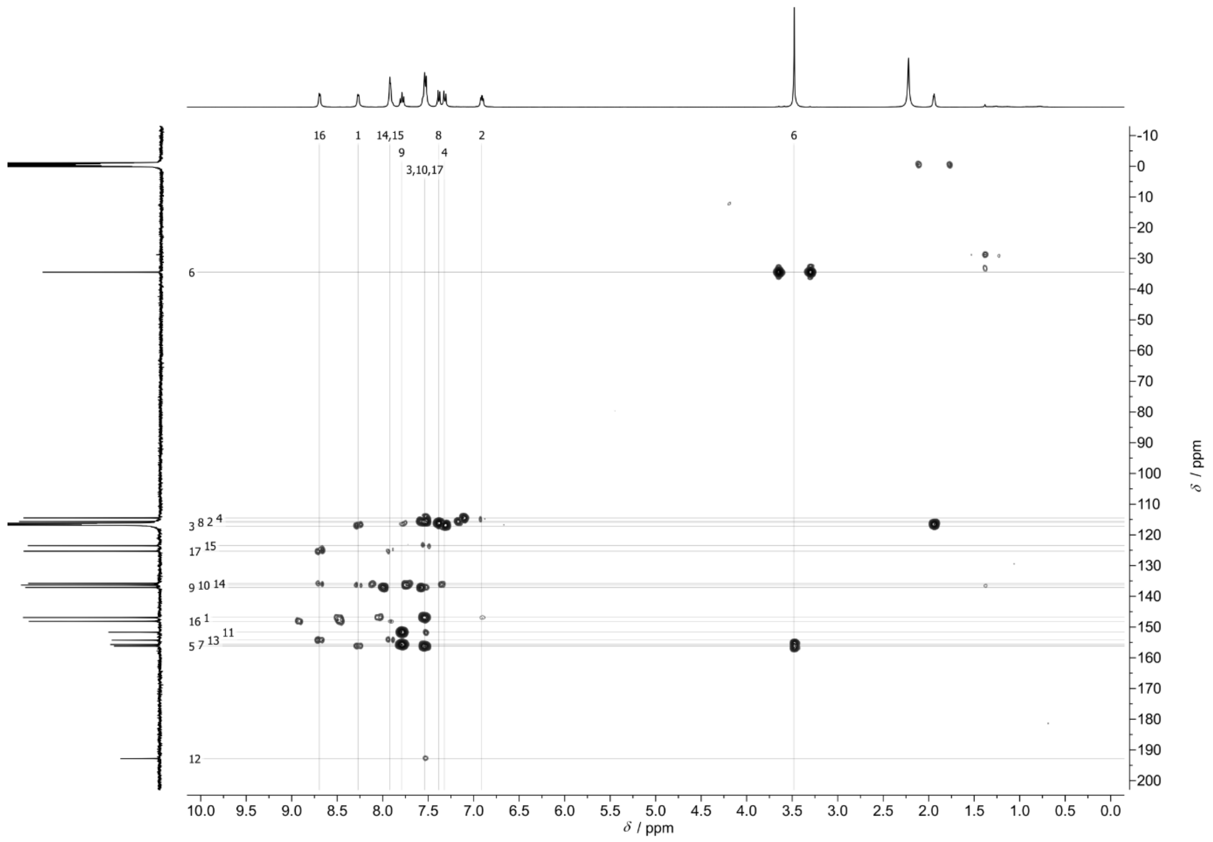

Figure S6. ^{1}H-^{13}C HMBC of cpmp in CD_3CN at 293 K.

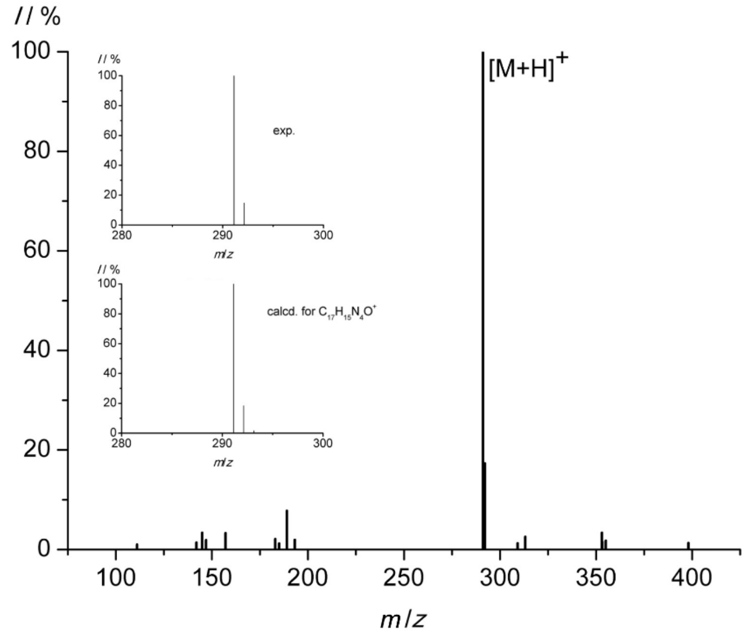

Figure S7. ESI^+ mass spectrum of cpmp in acetonitrile.

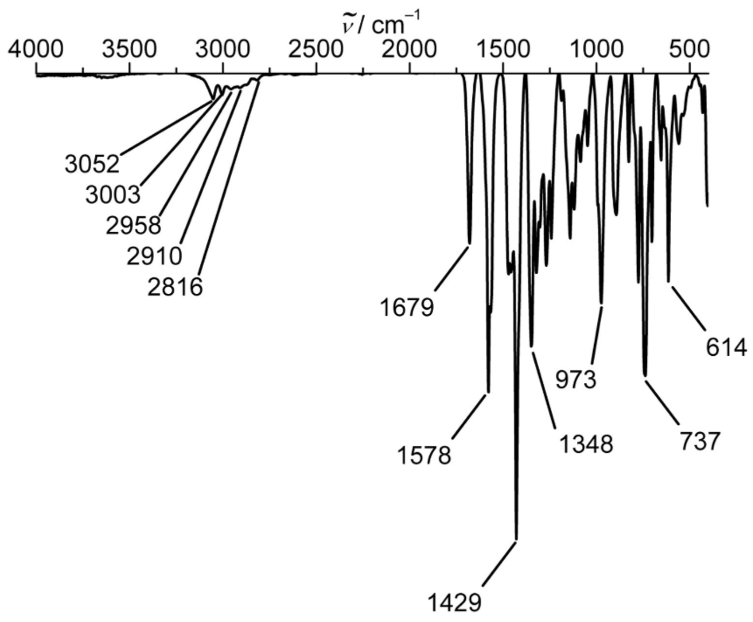

Figure S8. ATR-IR spectrum of cpmp.

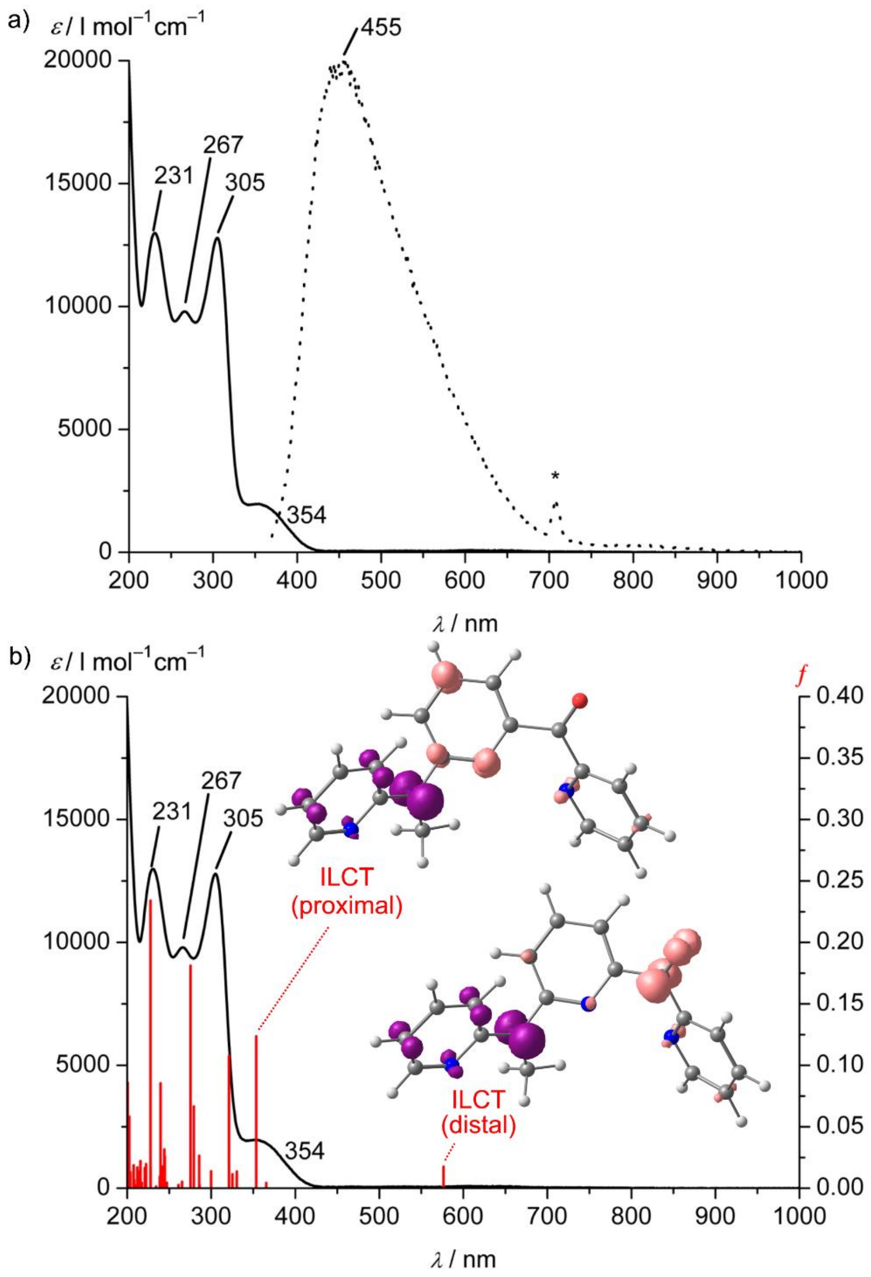

Figure S9. a) UV/Vis/NIR absorption and emission spectrum of cpmp in acetonitrile at 298 K (λ_{exc} = 354 nm, * = $2\lambda_{exc}$) and b) UV/Vis/NIR absorption spectrum (black line) and TD-DFT calculated oscillator strengths (red stick spectrum) of cpmp. Difference densities of the most intense low-energy ILCT transitions (TD-DFT, contour value of 0.01; purple = electron depletion; orange = electron gain). Possibly, the distal ILCT is very weak in solution due to the flexibility of cpmp, which is not reflected in the TD-DFT calculation of the static molecule.

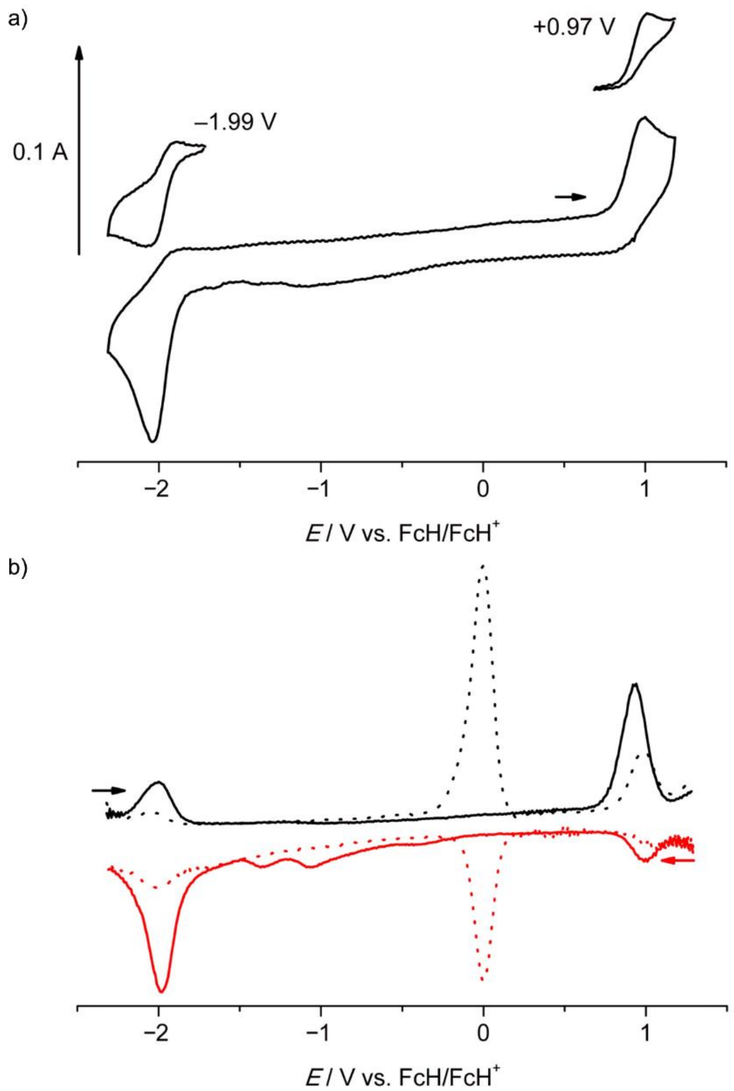

Figure S10. a) Cyclic voltammogram and b) square wave voltammogram of cpmp without (solid) and with (dotted) added ferrocene, 1 mM in acetonitrile, 0.1 M [^{n}Bu$_4$N][PF$_6$].

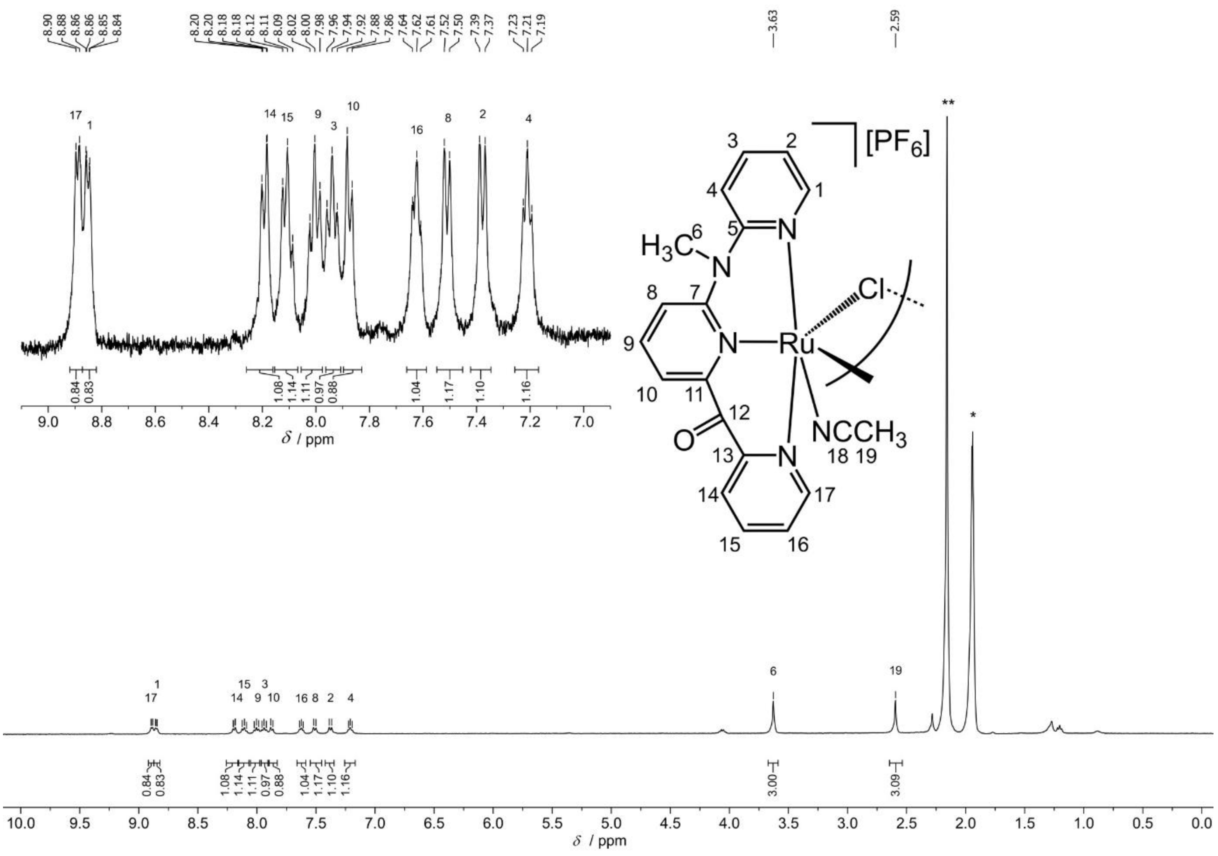

Figure S11. ^{1}H NMR spectrum of **1[PF₆]** in CD₃CN at 293 K. * denotes solvent resonance; ** denotes water resonance.

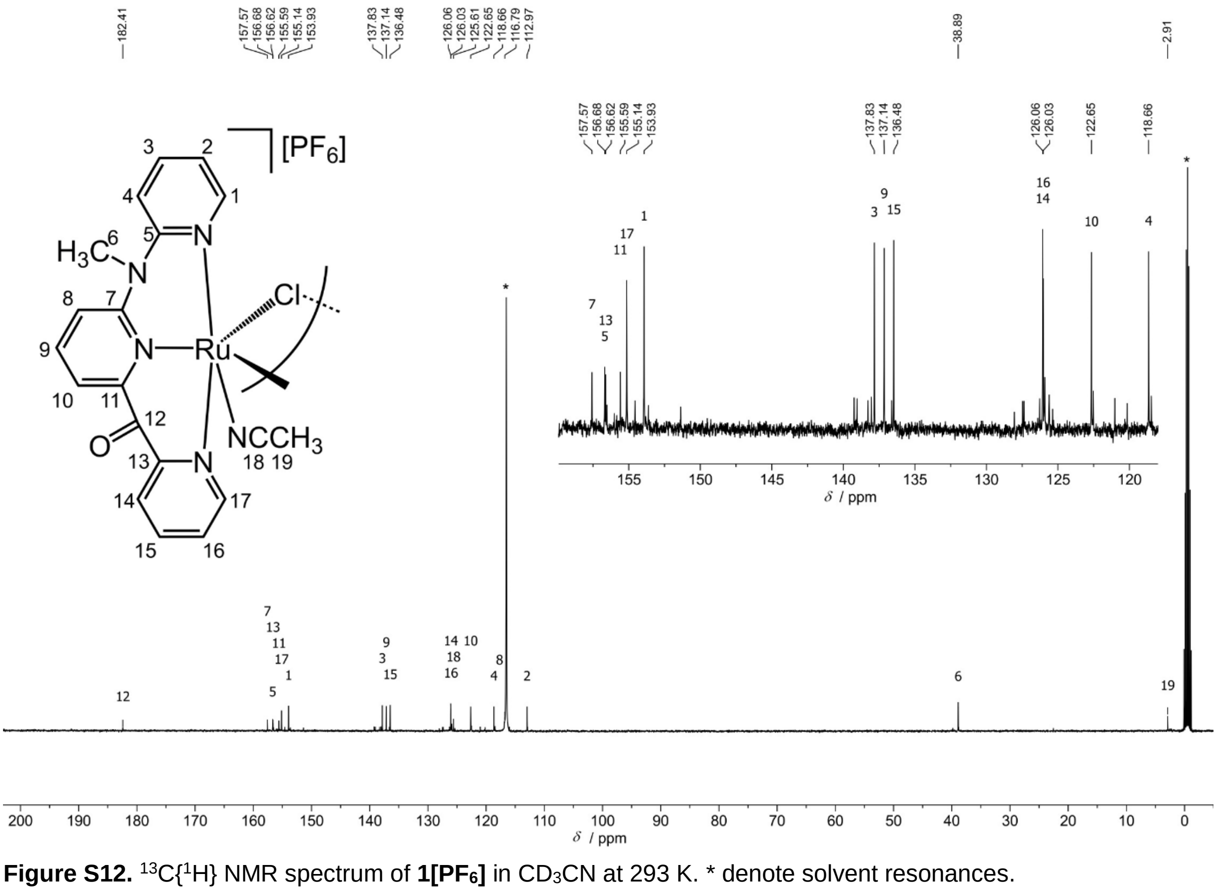

Figure S12. ^{13}C{^{1}H} NMR spectrum of **1[PF₆]** in CD₃CN at 293 K. * denote solvent resonances.

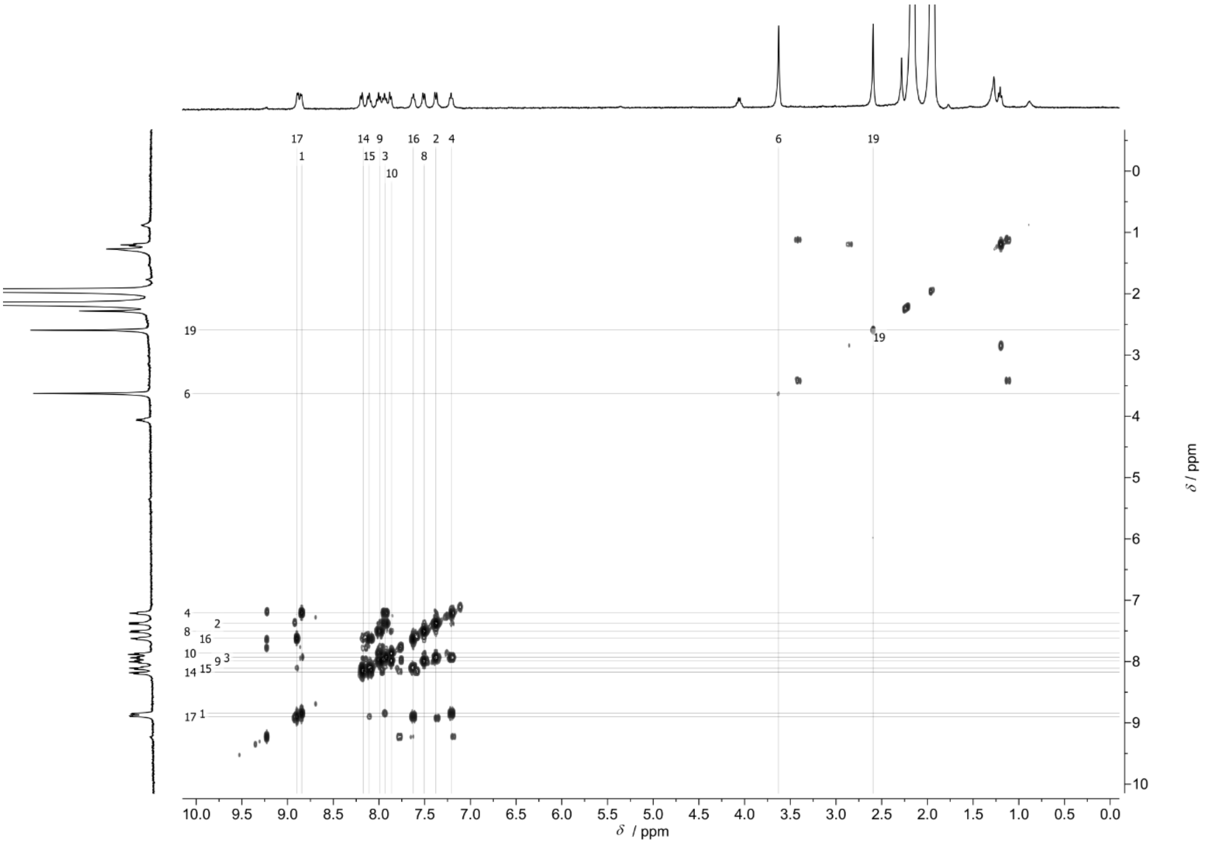

Figure S13. ^{1}H-^{1}H COSY of **1[PF$_6$]** in CD_3CN at 293 K.

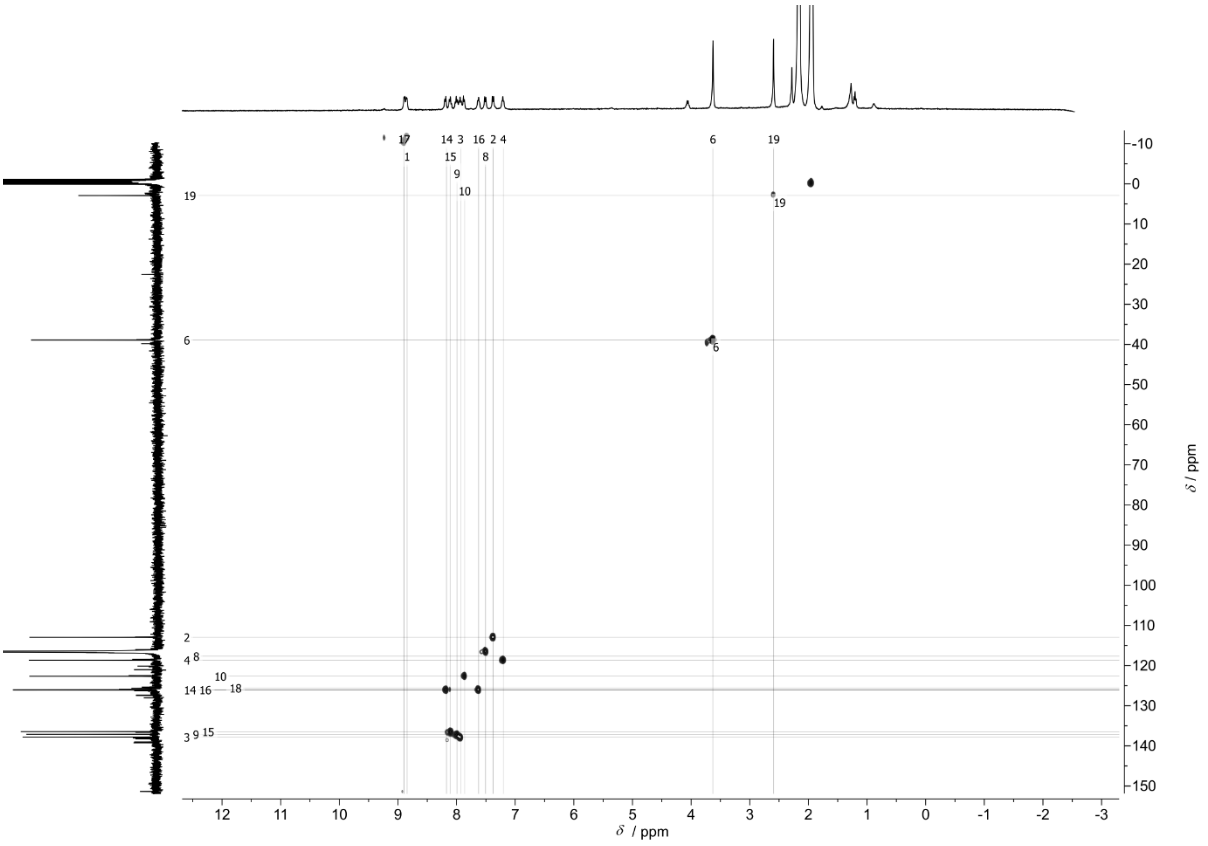

Figure S14. ^{1}H-^{13}C HSQC of **1[PF$_6$]** in CD_3CN at 293 K.

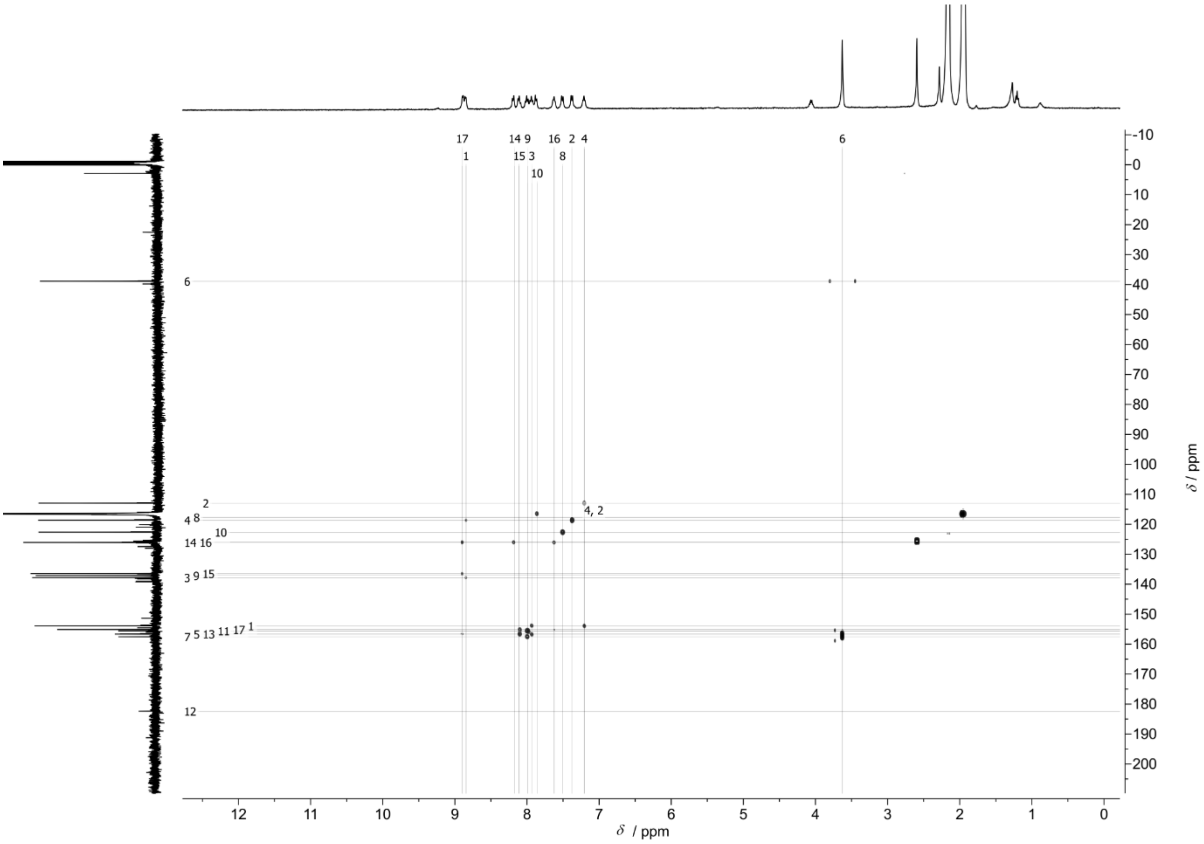

Figure S15. ^{1}H-^{13}C HMBC of **1[PF$_6$]** in CD_3CN at 293 K.

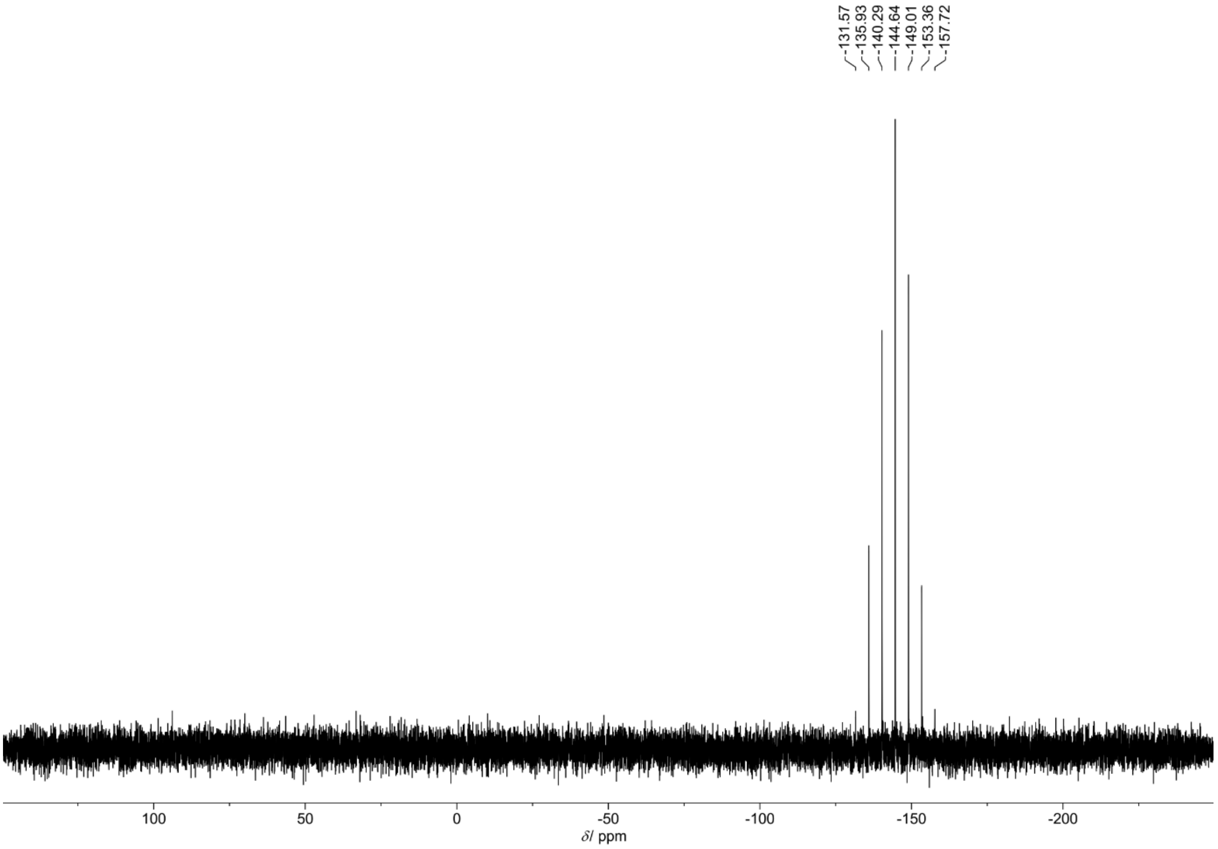

Figure S16. $^{31}P\{^{1}H\}$ NMR spectrum of **1[PF$_6$]** in CD_3CN at 293 K.

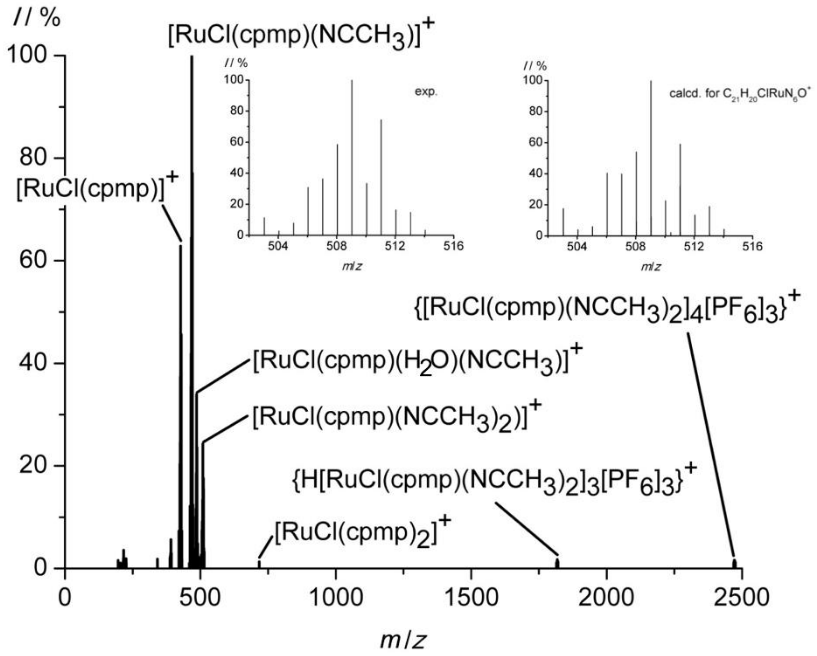

Figure S17. ESI$^+$ mass spectrum of **1[PF$_6$]** in acetonitrile.

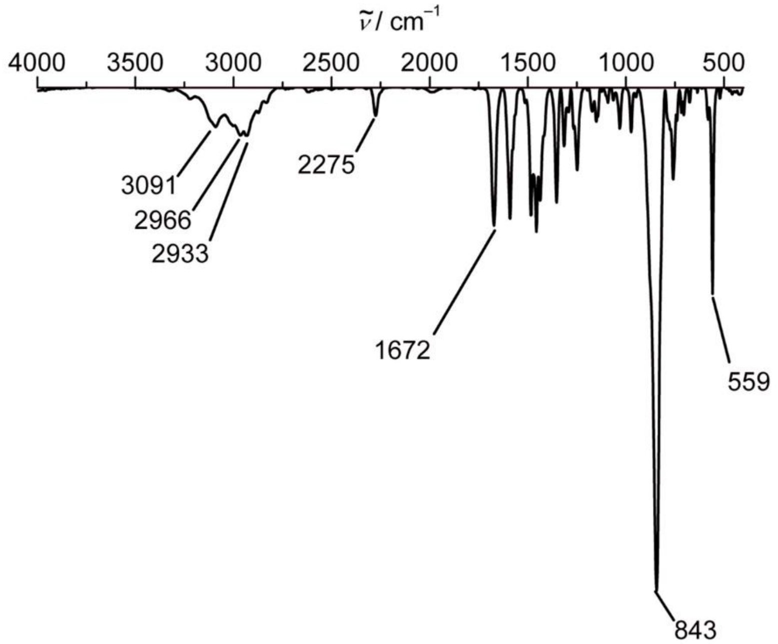

Figure S18. IR spectrum of **1[PF$_6$]** in KBr.

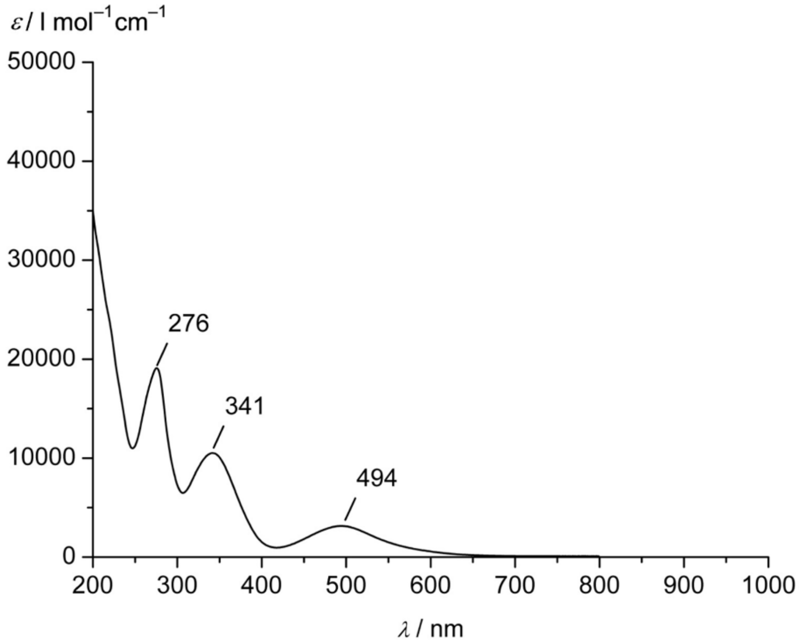

Figure S19. UV/Vis/NIR absorption spectrum of **1[PF_6]** in acetonitrile at 298 K.

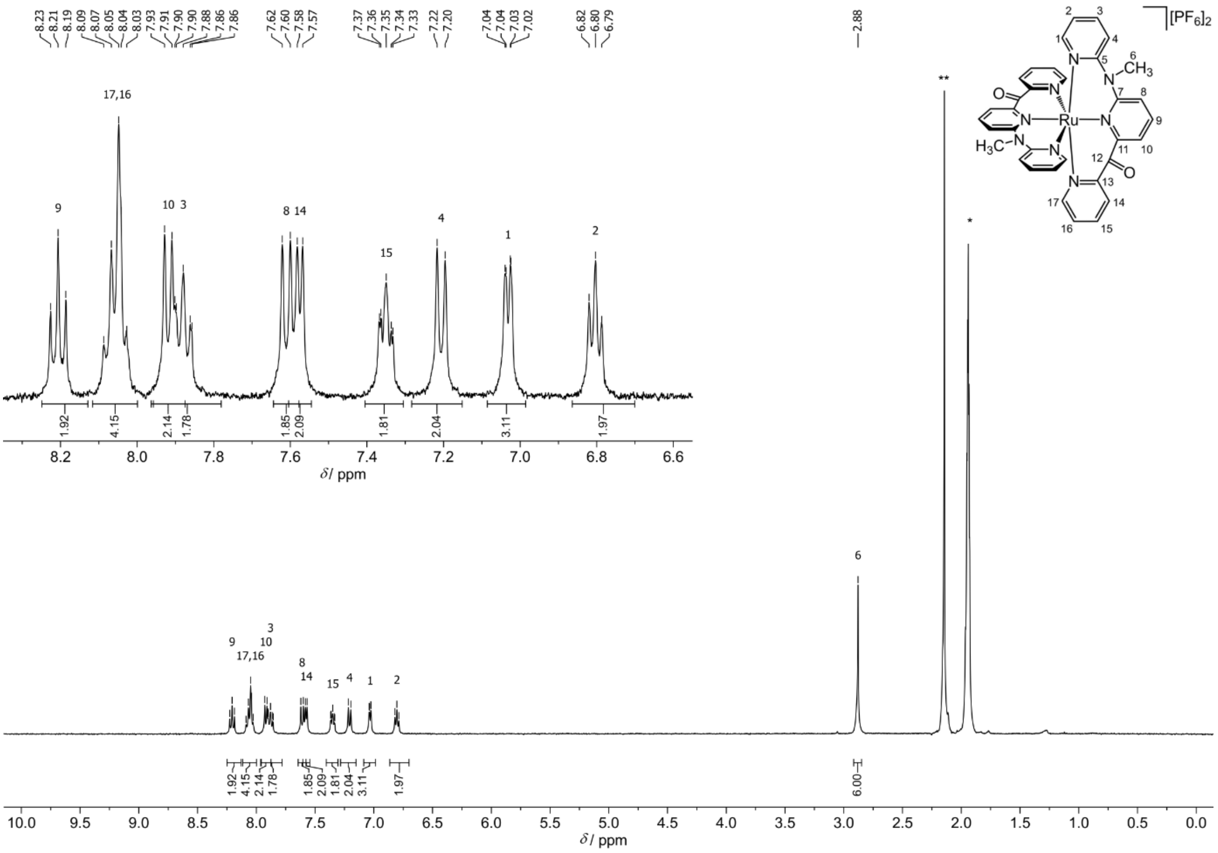

Figure S20. ^{1}H NMR spectrum of **2[PF$_6$]$_2$** in CD_3CN at 293 K. * denotes solvent resonance; ** denotes water resonance.

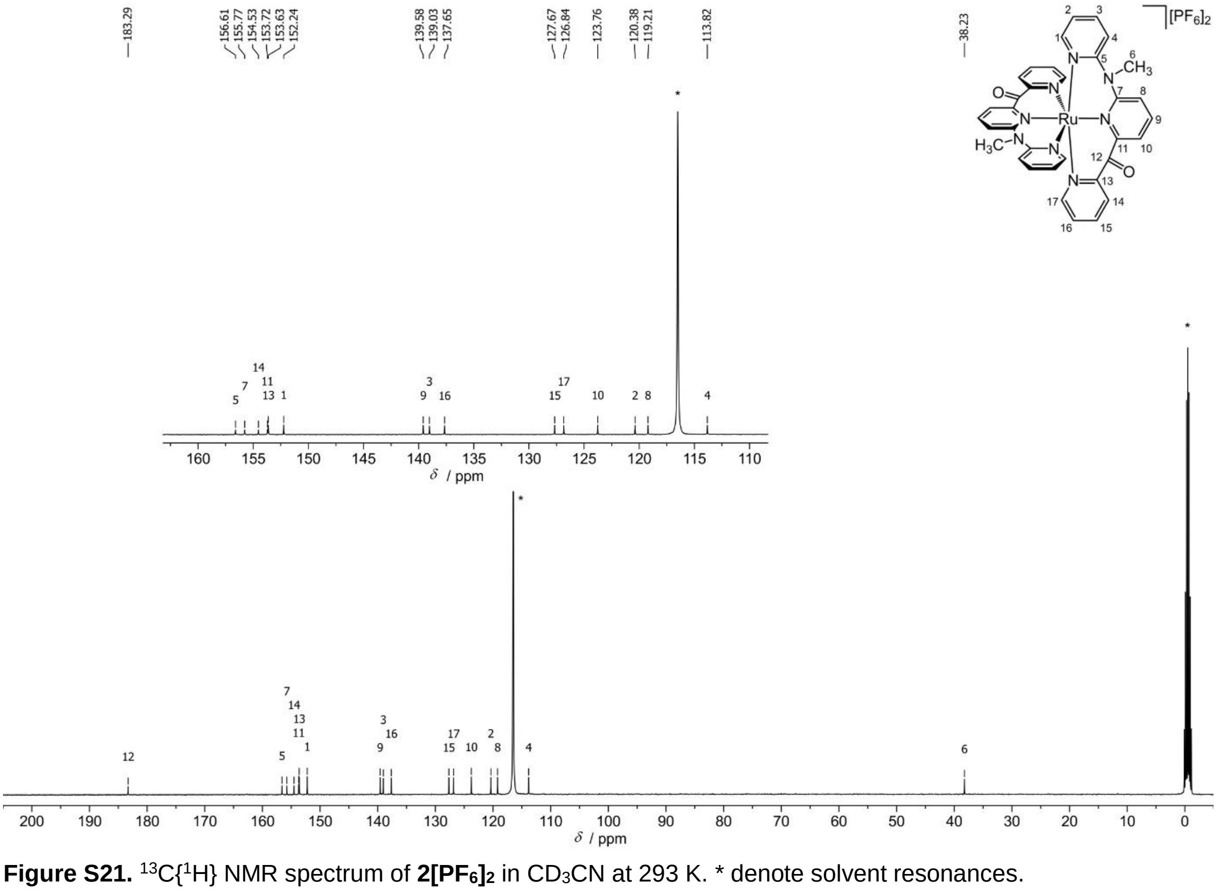

Figure S21. ^{13}C{^{1}H} NMR spectrum of **2[PF$_6$]$_2$** in CD_3CN at 293 K. * denote solvent resonances.

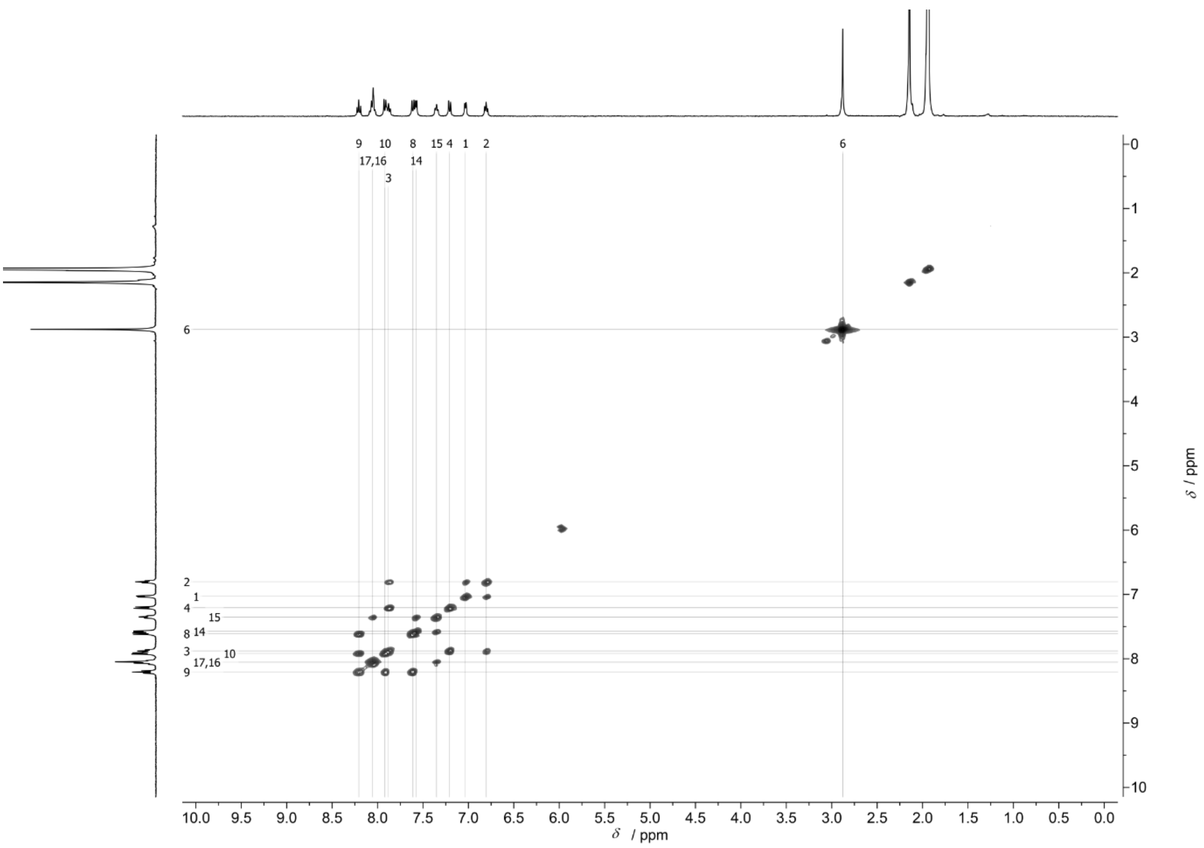

Figure S22. ^{1}H-^{1}H COSY of **2[PF$_6$]$_2$** in CD_3CN at 293 K.

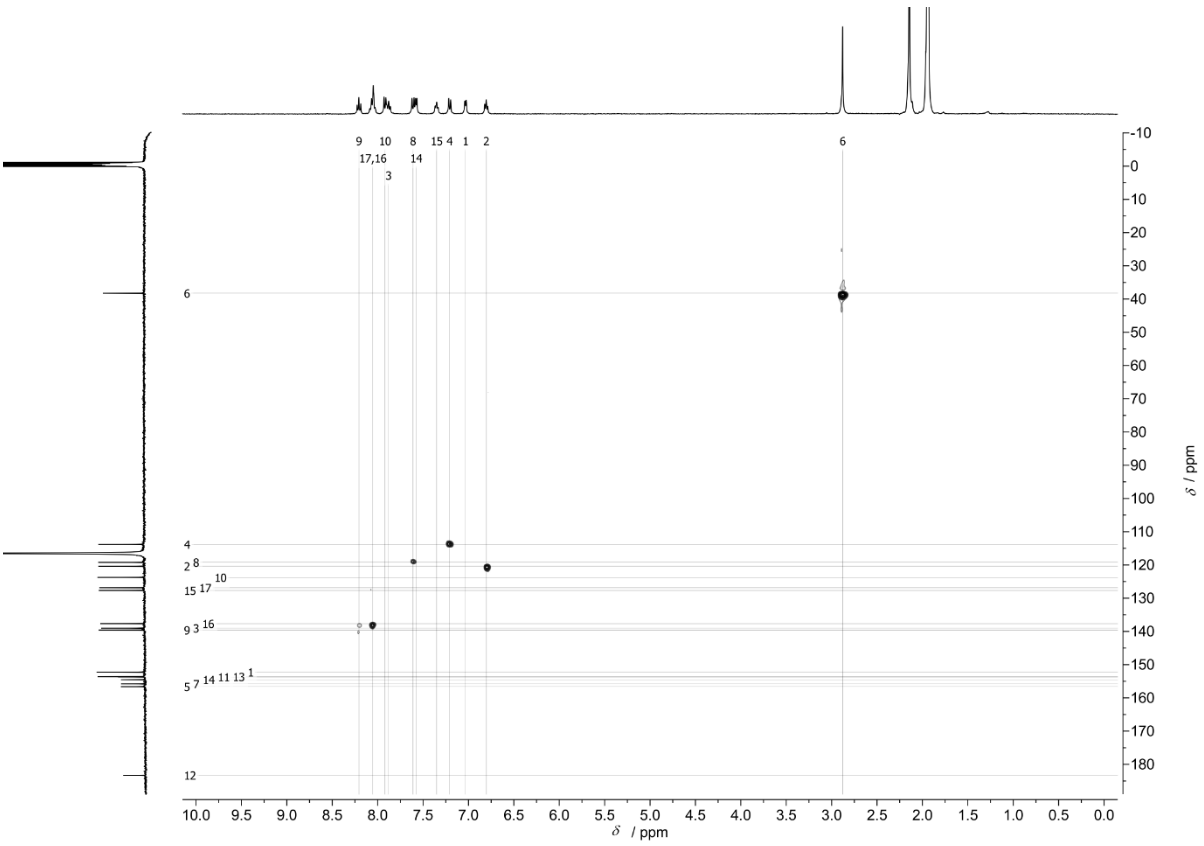

Figure S23. ^{1}H-^{13}C HSQC of **2[PF$_6$]$_2$** in CD_3CN at 293 K.

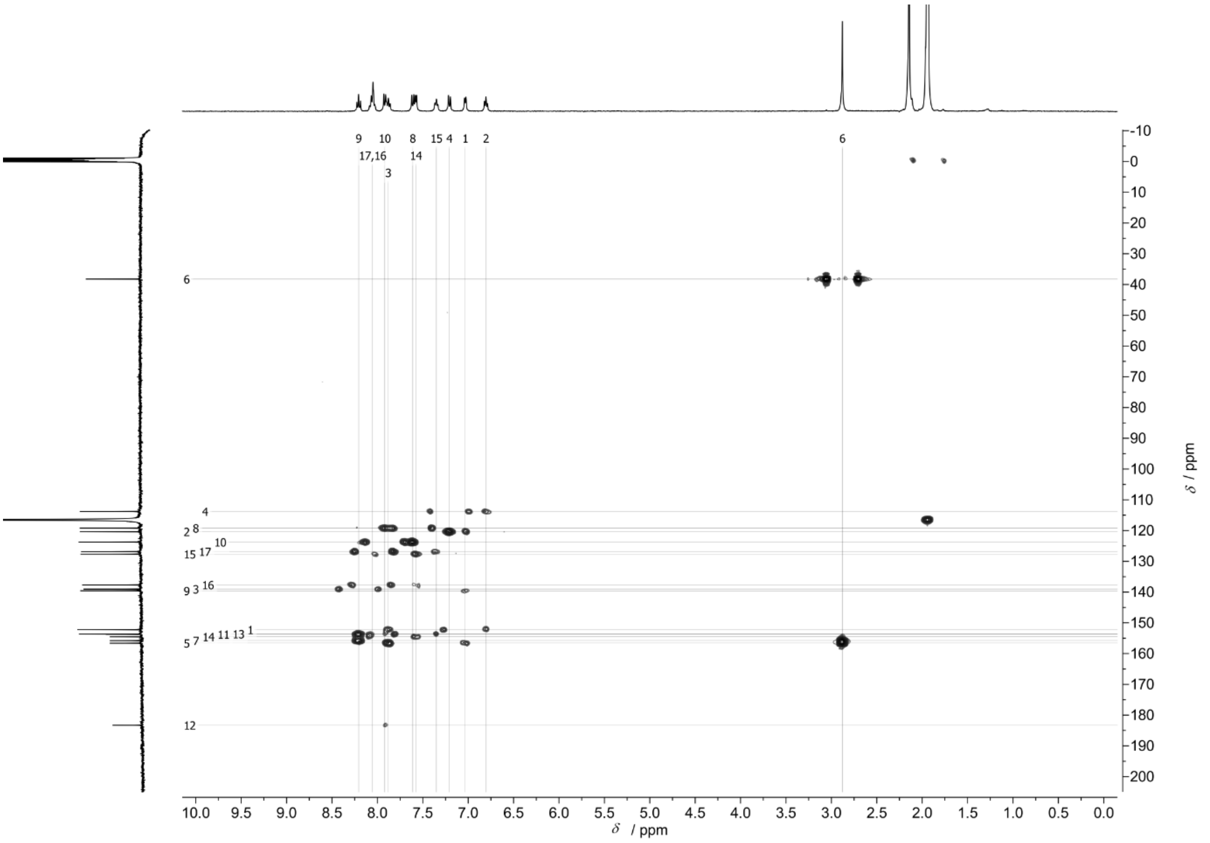

Figure S24. ^{1}H-^{13}C HMBC of **2[PF$_6$]$_2$** in CD_3CN at 293 K.

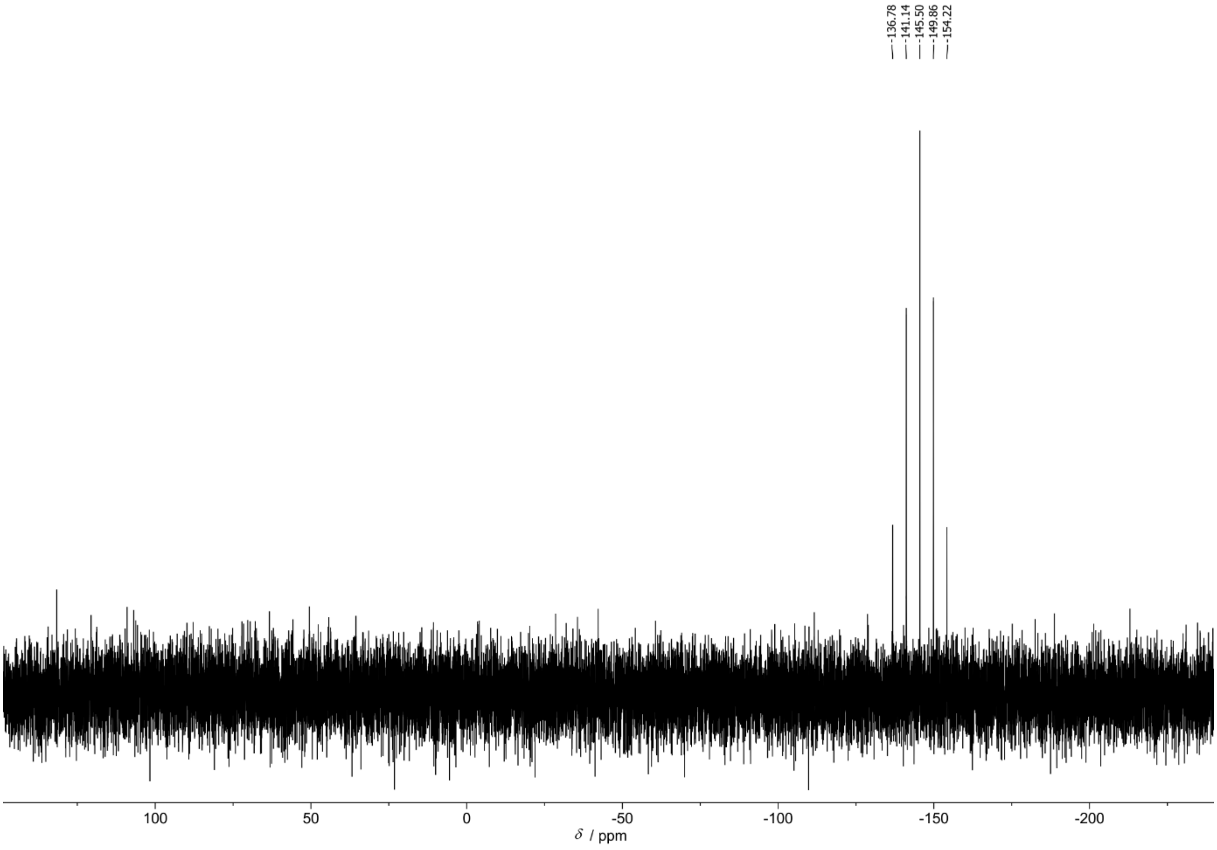

Figure S25. $^{31}P\{^{1}H\}$ NMR spectrum of **2[PF$_6$]$_2$** in CD_3CN at 293 K.

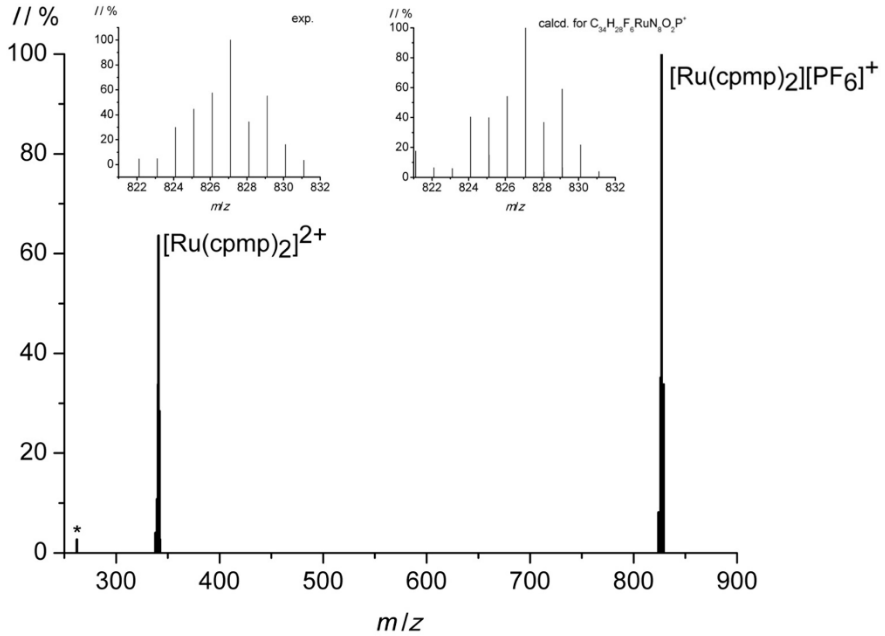

Figure S26. ESI$^+$ mass spectrum of **2[PF$_6$]$_2$** in acetonitrile. * denotes an impurity in the ESI spectrometer.

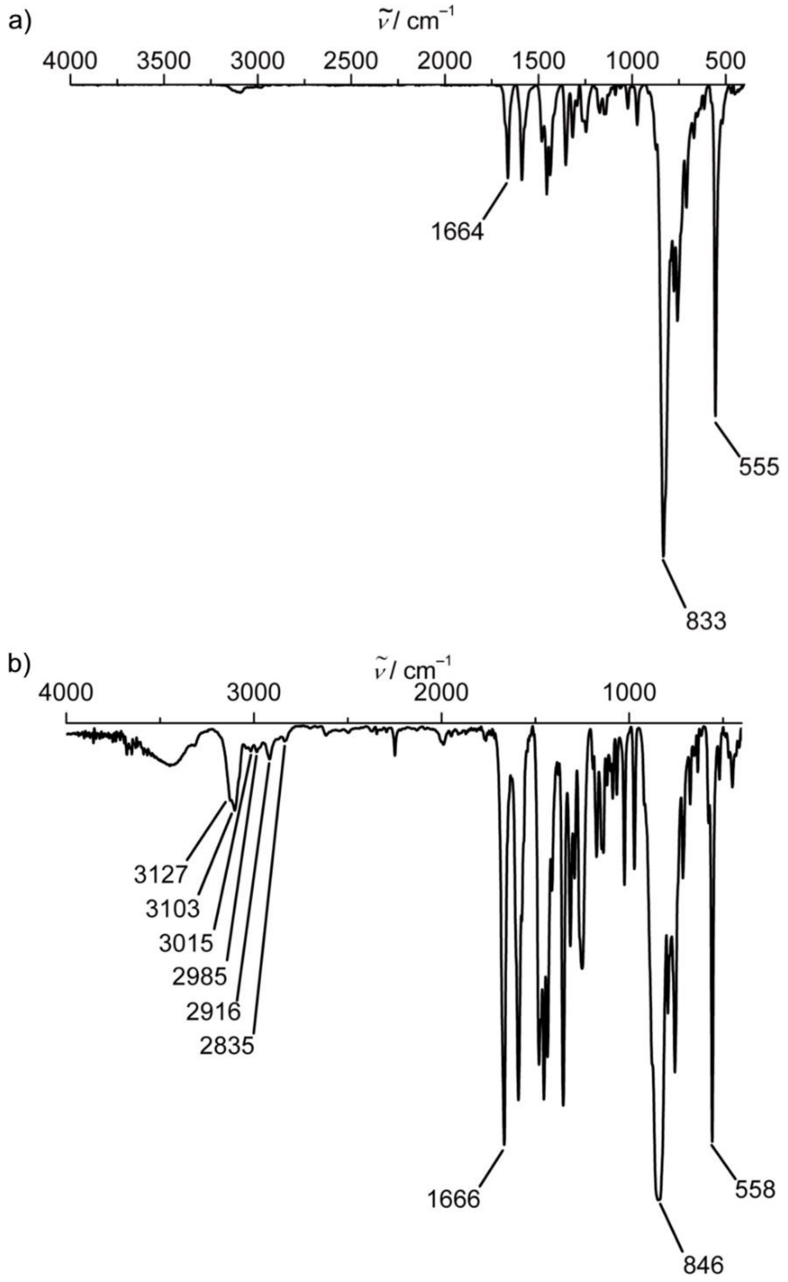

Figure S27. a) ATR-IR spectrum of **2[PF$_6$]$_2$** and b) IR spectrum of **2[PF$_6$]$_2$** in KBr.

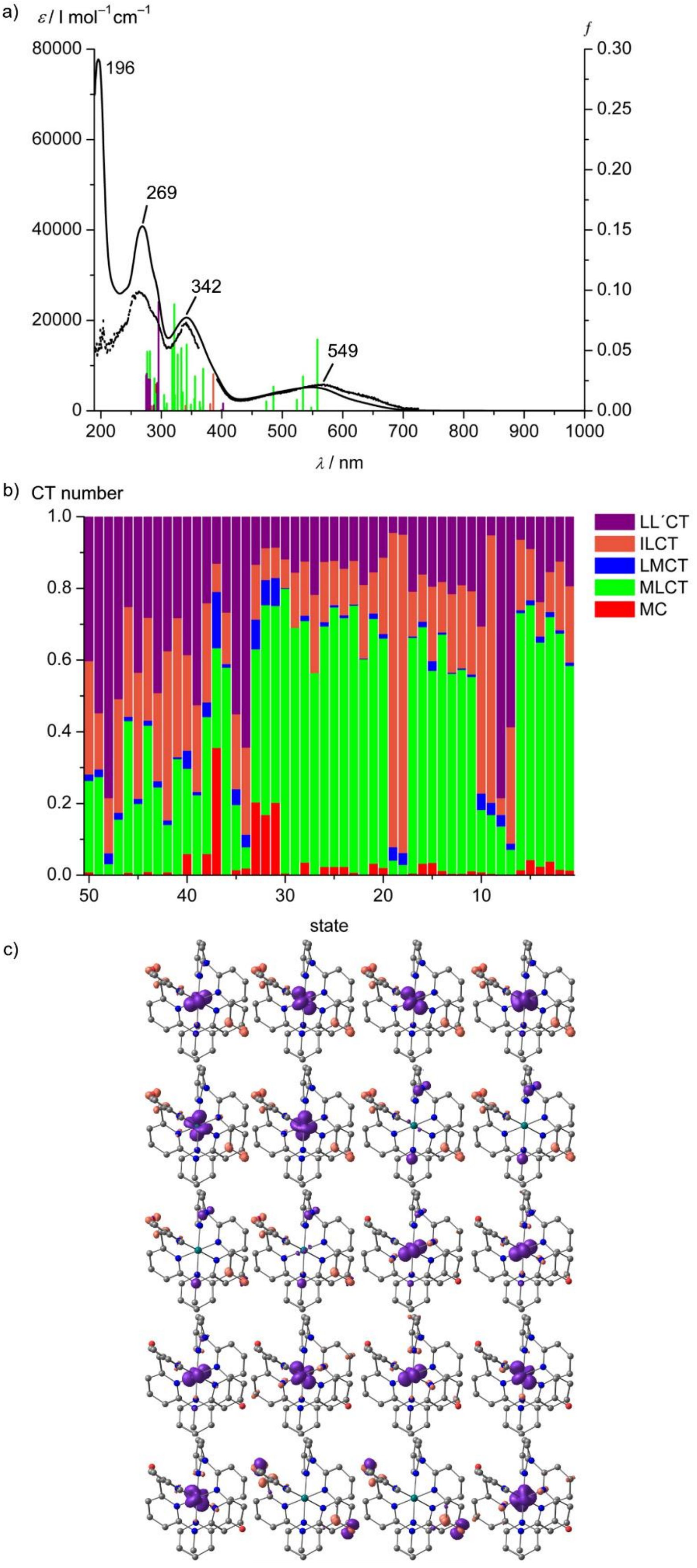

Figure S28. a) UV/Vis/NIR absorption (black solid) and excitation spectrum (black dotted, λ_{em} = 709 nm) of **2[PF$_6$]$_2$** in acetonitrile at 298 K and TD-DFT calculated oscillator strengths of **2^{2+}** (stick spectrum, color code according to charge transfer (CT) numbers); b) TD-DFT CT numbers from 0 to 1 of the 50 lowest lying singlets and c) difference densities of 20 lowest energy transitions (TD-DFT, contour value of 0.01; purple = electron depletion; orange = electron gain).

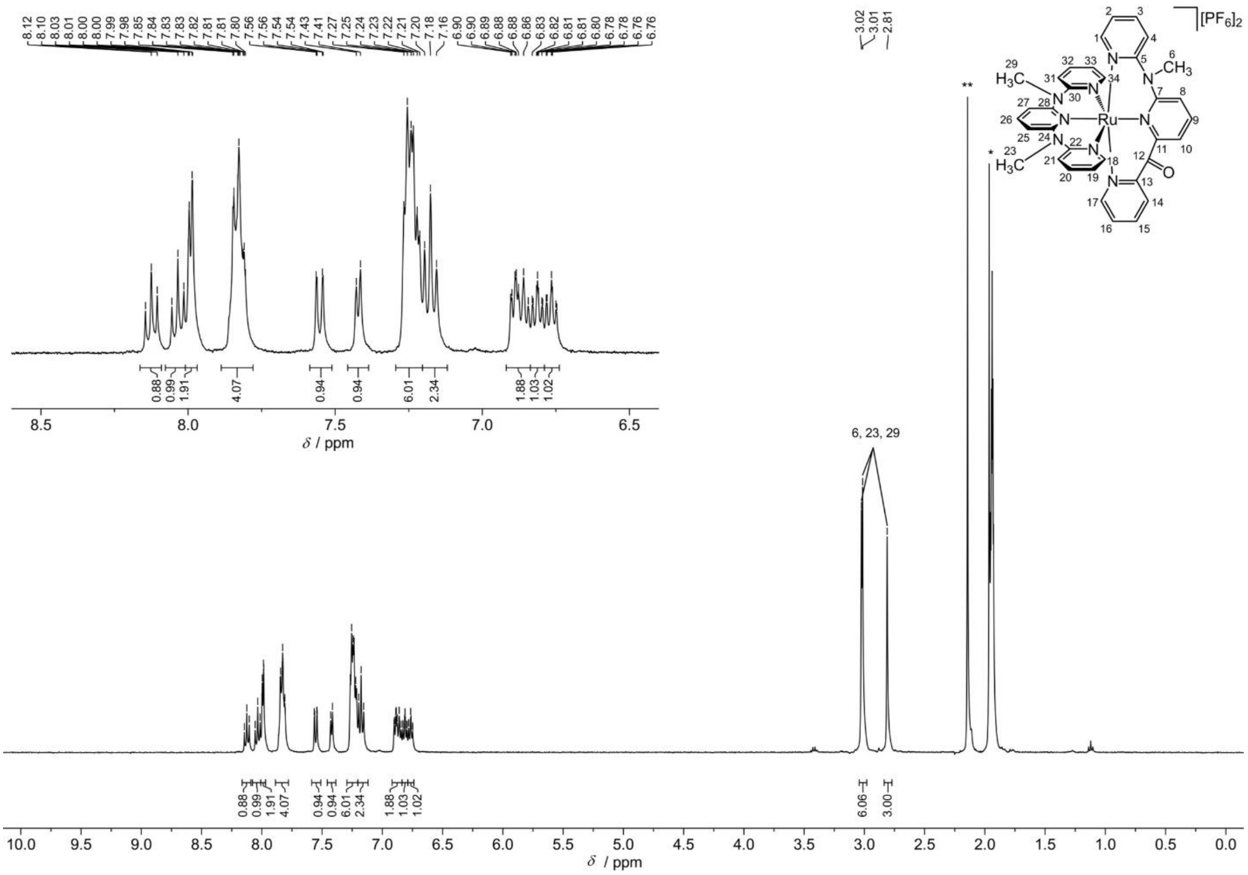

Figure S29. ^{1}H NMR spectrum of **3[PF$_6$]$_2$** in CD_3CN at 293 K. * denotes solvent resonance; ** denotes water resonance.

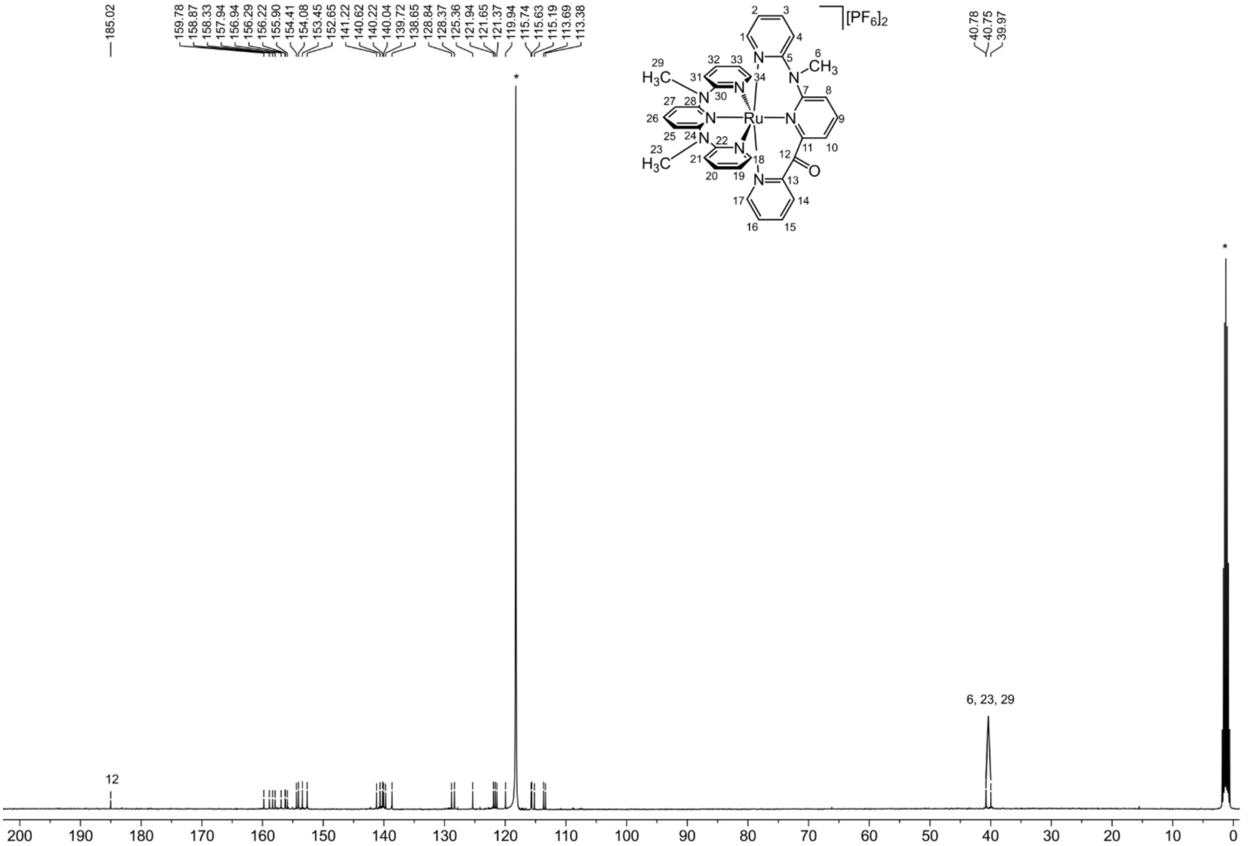

Figure S30. $^{13}C\{^{1}H\}$ NMR spectrum of **3[PF$_6$]$_2$** in CD_3CN at 293 K. * denote solvent resonances.

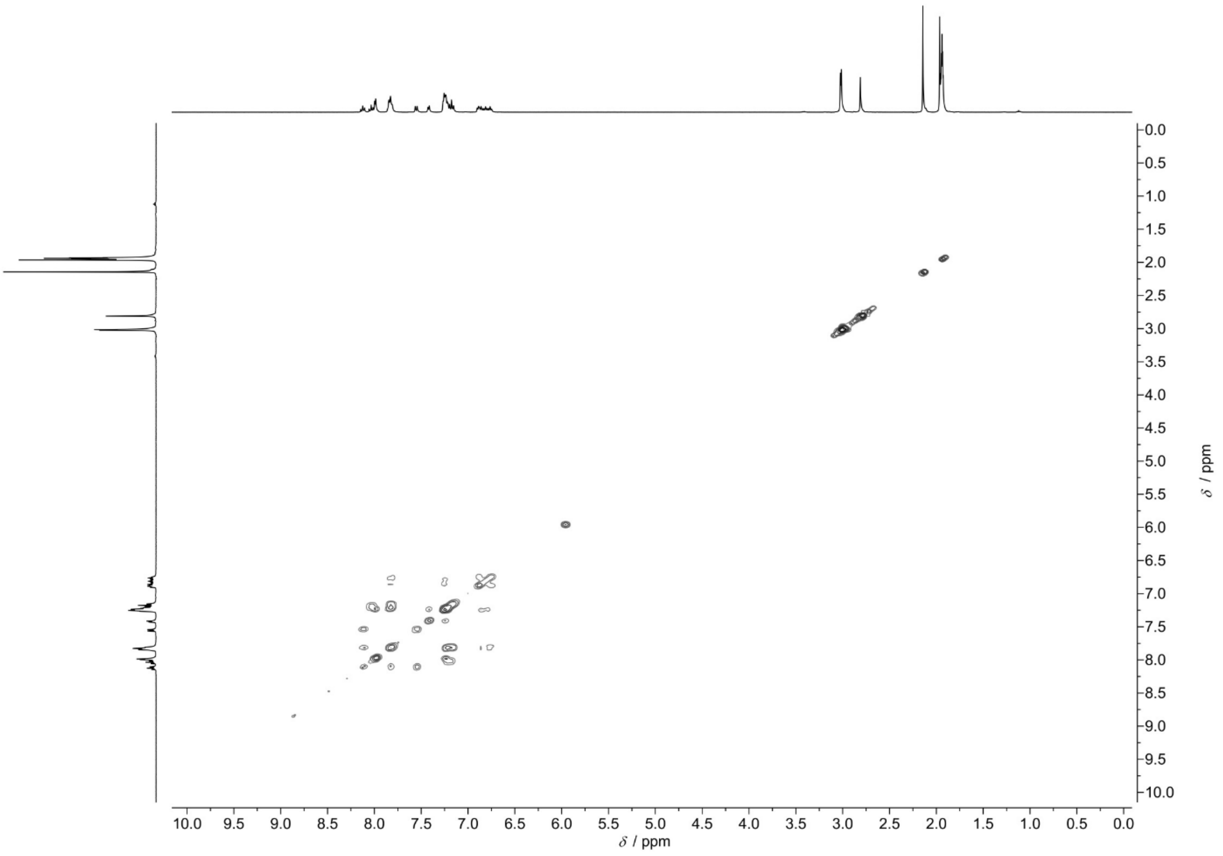

Figure S31. ^{1}H-^{1}H-COSY of **3[PF$_6$]$_2$** in CD_3CN at 293 K.

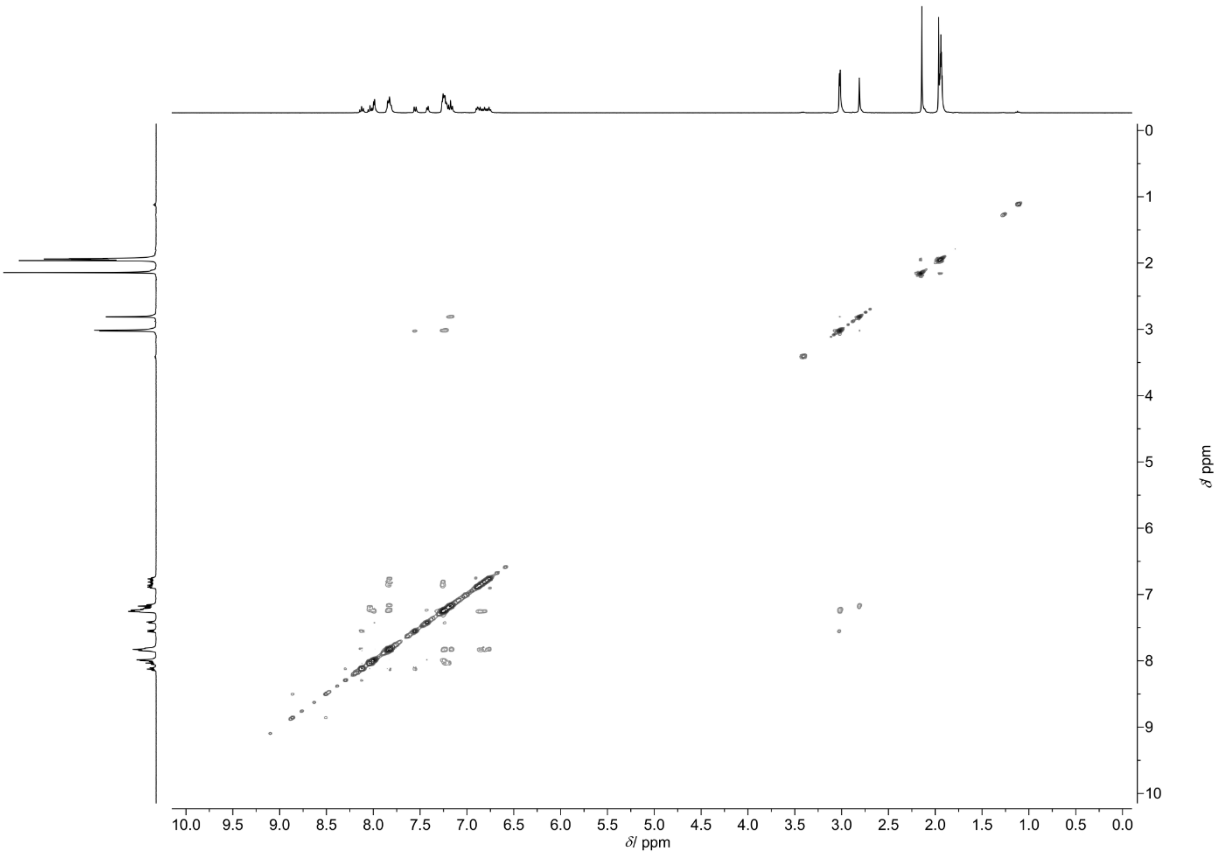

Figure S32. ^{1}H-^{1}H-NOESY of **3[PF$_6$]$_2$** in CD_3CN at 293 K (t_{mix} = 1 s).

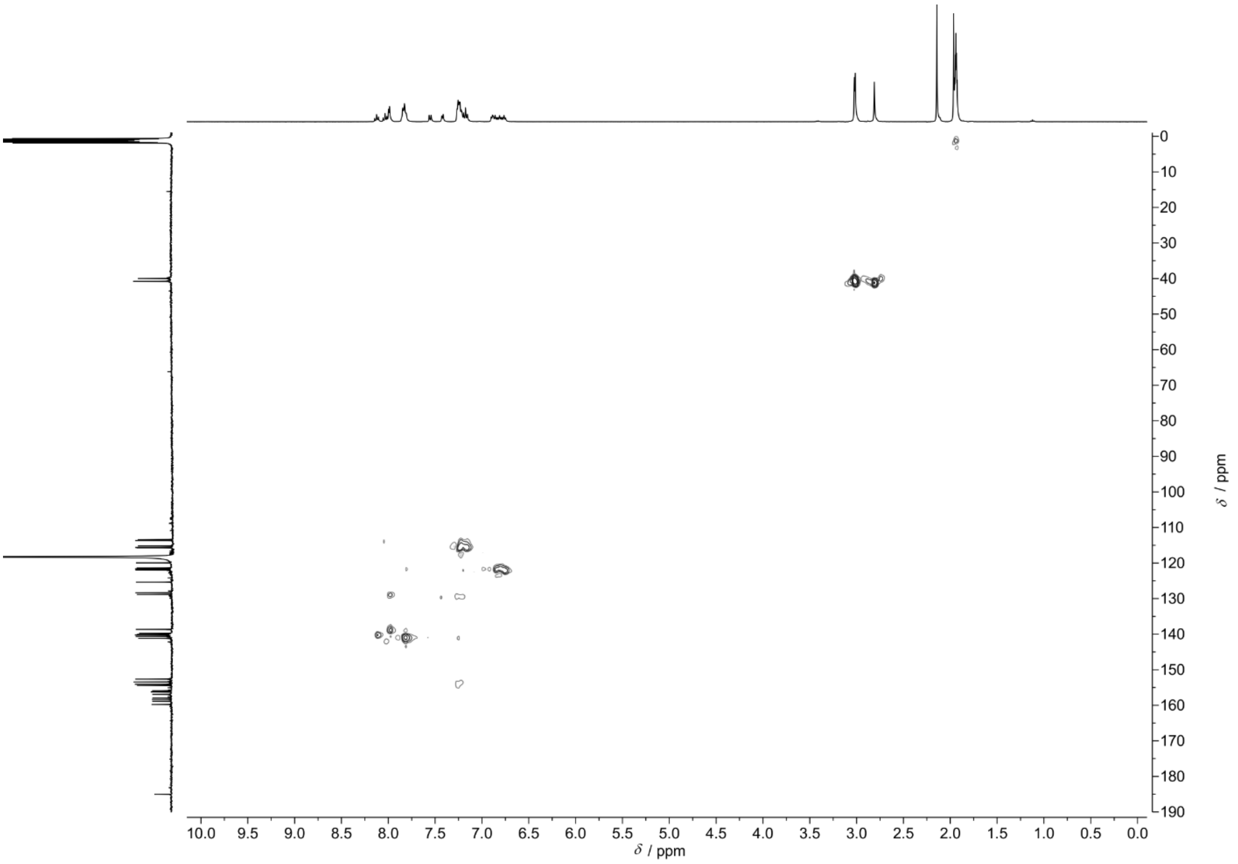

Figure S33. ^{1}H-^{13}C HSQC of **3[PF$_6$]$_2$** in CD_3CN at 293 K.

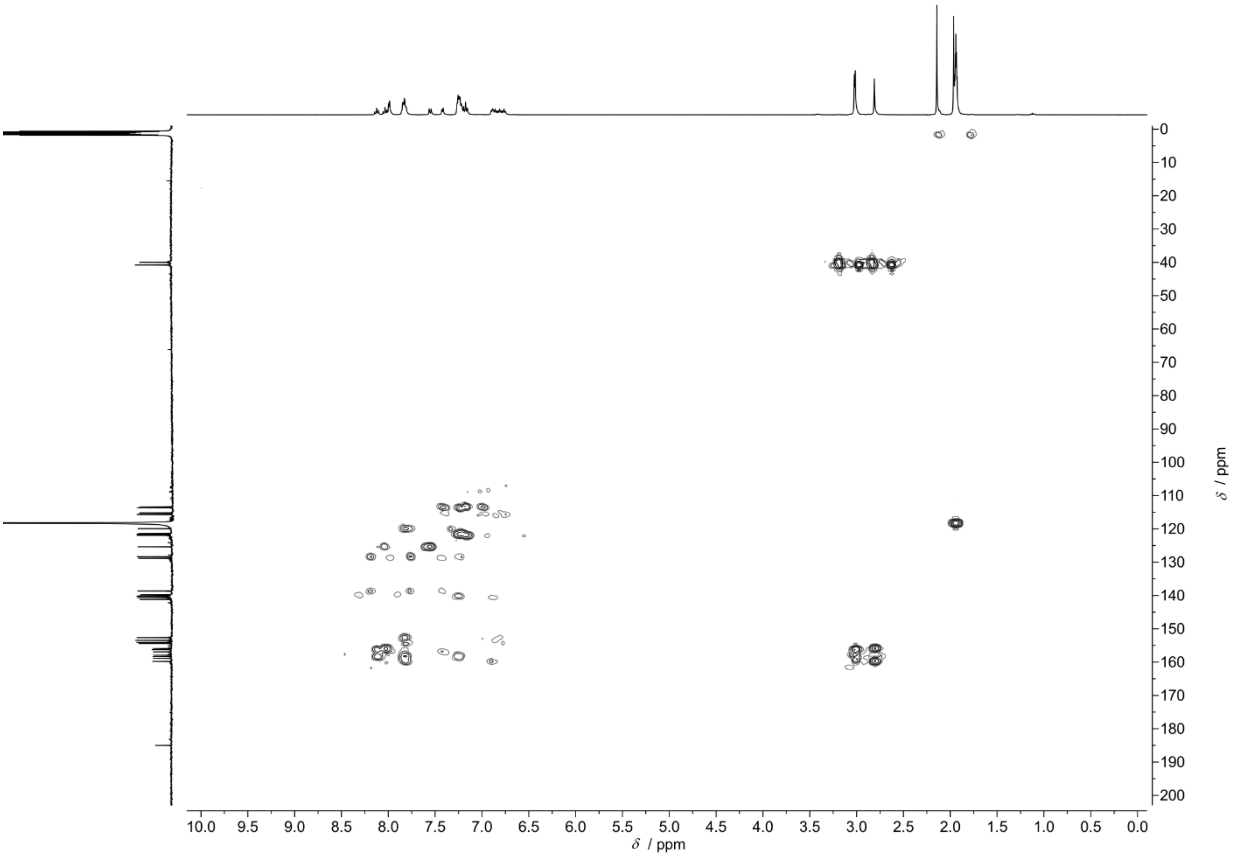

Figure S34. ^{1}H-^{13}C-HMBC of **3[PF$_6$]$_2$** in CD_3CN at 293 K.

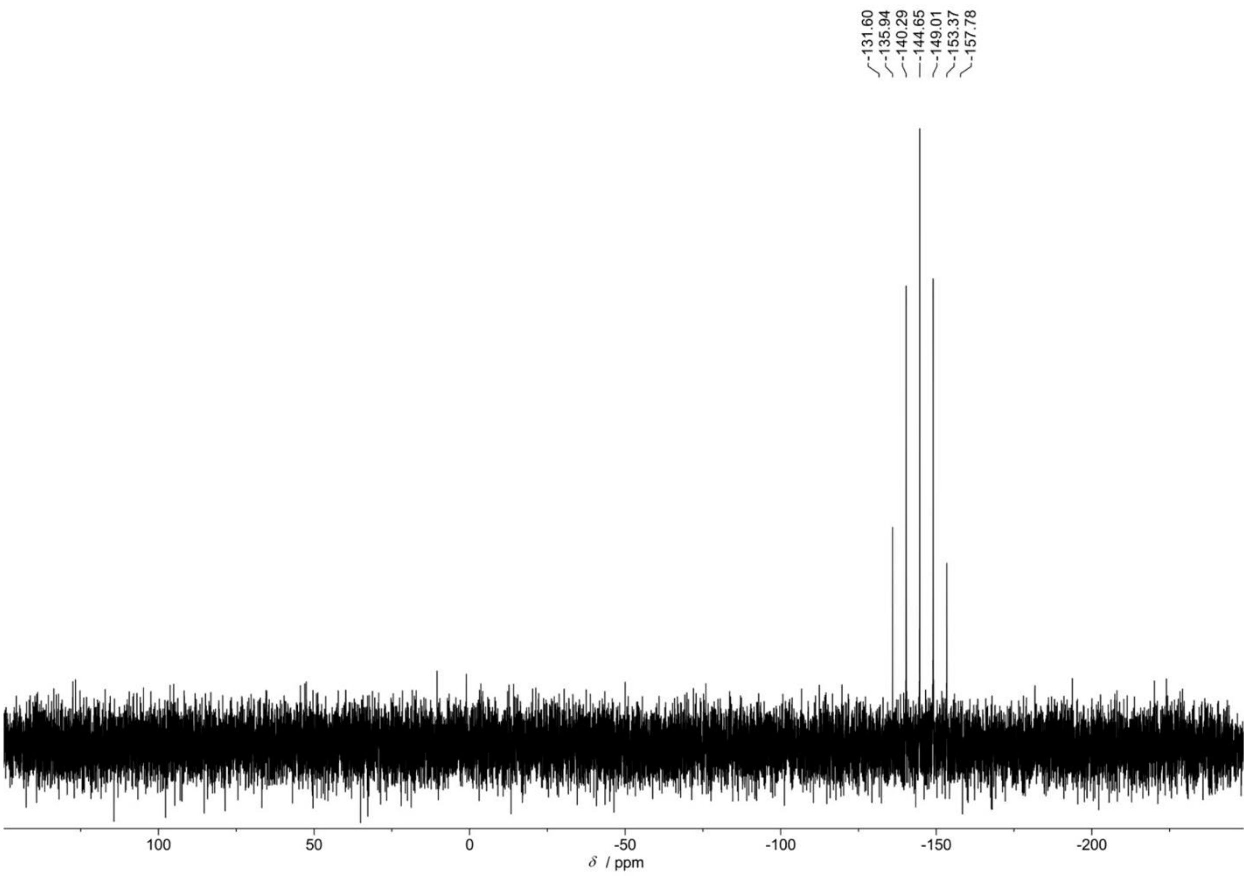

Figure S35. $^{31}P\{^{1}H\}$ NMR spectrum of **3[PF$_6$]$_2$** in CD_3CN at 293 K.

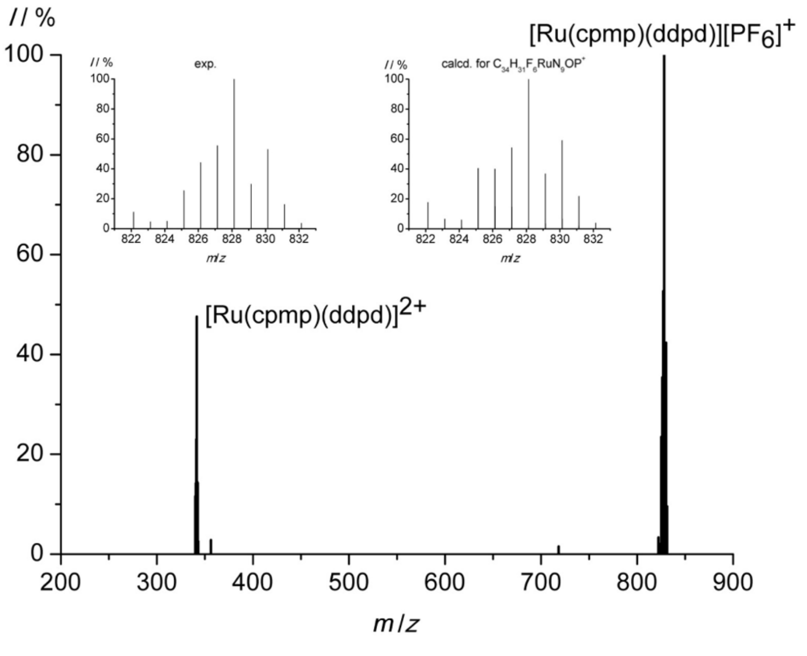

Figure S36. ESI$^+$ mass spectrum of **3[PF$_6$]$_2$** in acetonitrile.

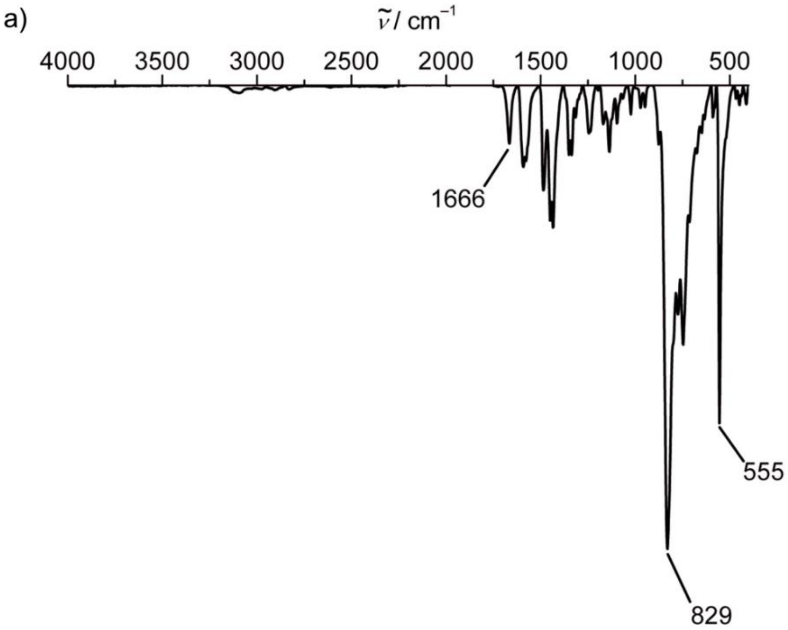

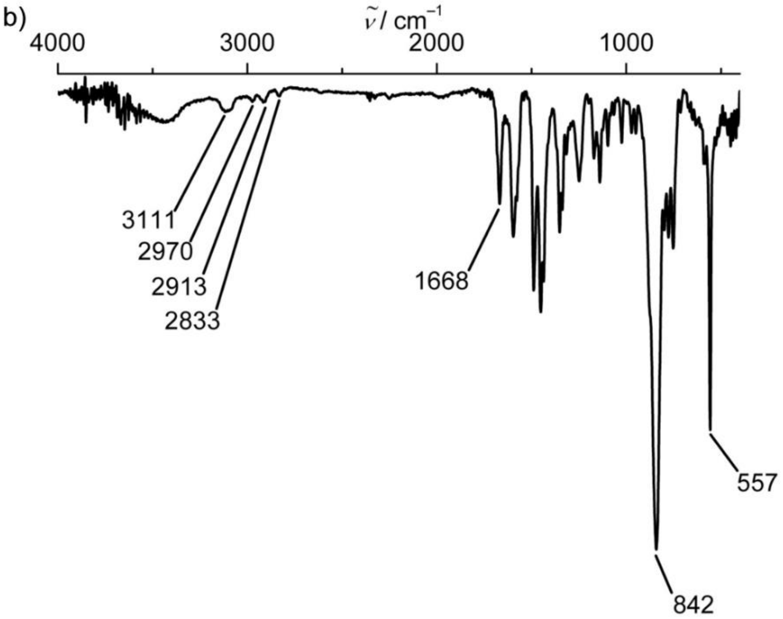

Figure S37. a) ATR-IR spectrum of **3[PF$_6$]$_2$** and b) IR spectrum of **3[PF$_6$]$_2$** in KBr.

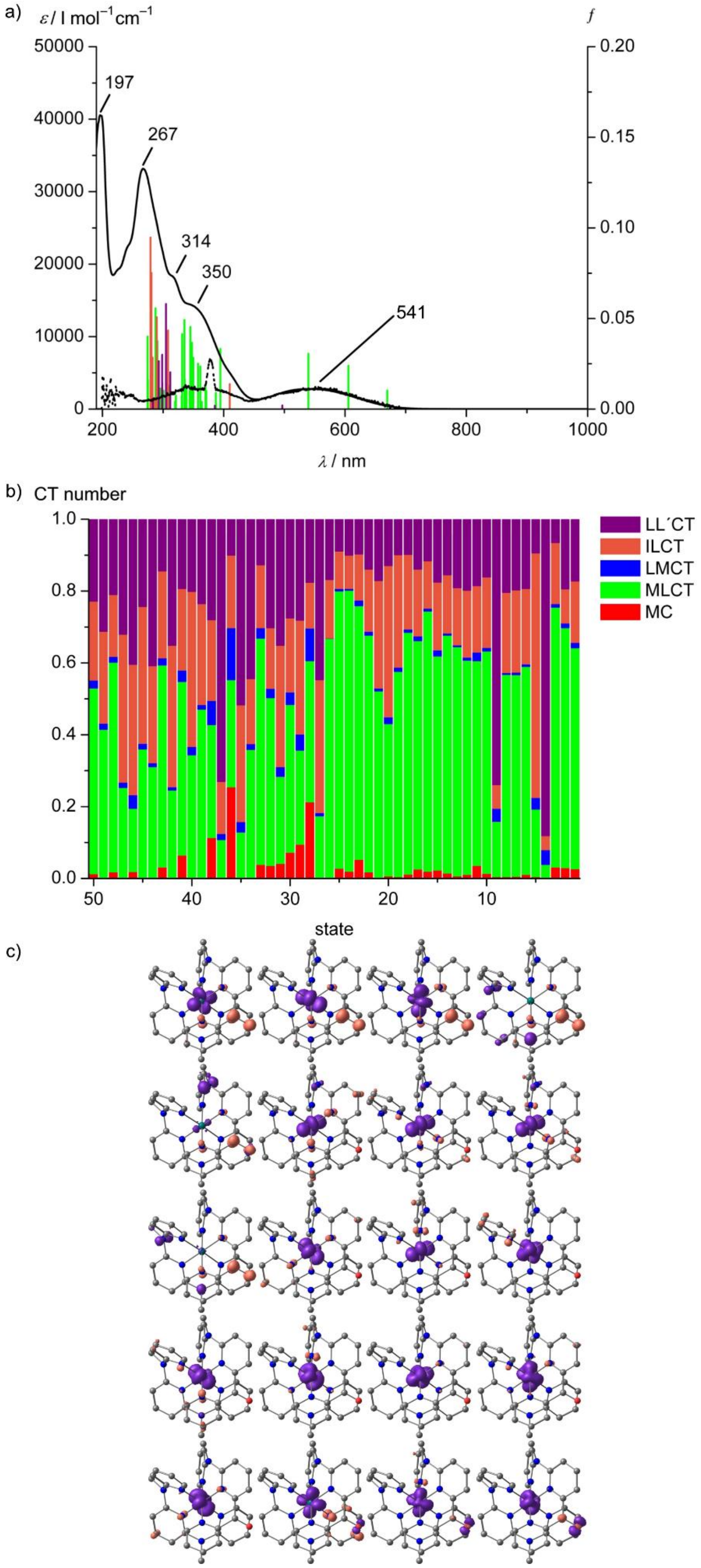

Figure S38. a) UV/Vis/NIR absorption (black solid) and excitation spectrum (black dotted, λ_{em} = 755 nm) of **3[PF$_6$]$_2$** in acetonitrile at 298 K and TD-DFT calculated oscillator strengths of **3^{2+}** (stick spectrum, color code according to charge transfer (CT) numbers); b) TD-DFT CT numbers from 0 to 1 of the 50 lowest lying singlets and c) difference densities of 20 lowest energy transitions (TD-DFT, contour value of 0.01; purple = electron depletion; orange = electron gain).

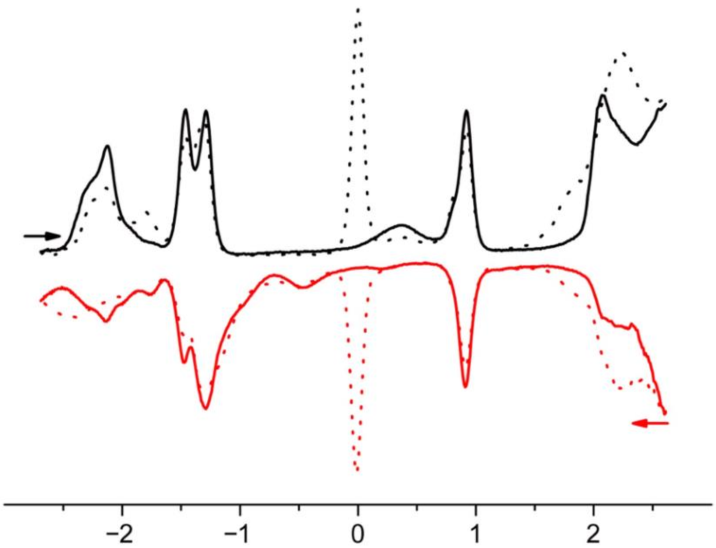

Figure S39. Square wave voltammogram of **2[PF$_6$]$_2$** without (solid) and with (dotted) added ferrocene, 1 mM in acetonitrile, 0.1 M [^{n}Bu$_4$N][PF$_6$].

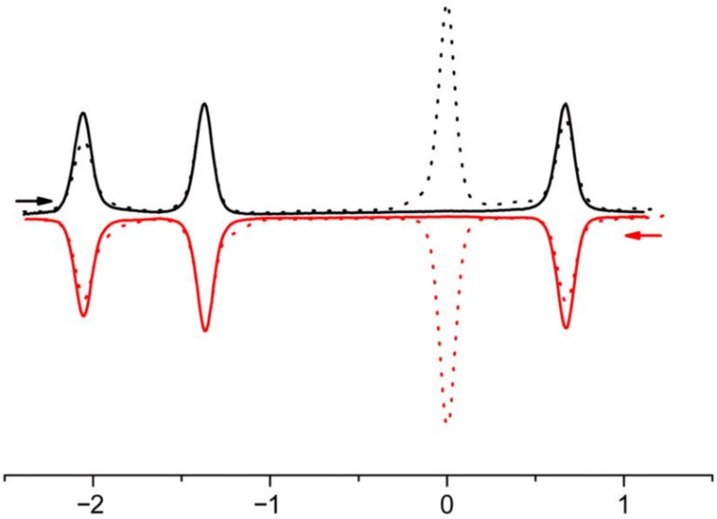

Figure S40. Square wave voltammogram of **3[PF$_6$]$_2$** without (solid) and with (dotted) added ferrocene, 1 mM in acetonitrile, 0.1 M [^{n}Bu$_4$N][PF$_6$].

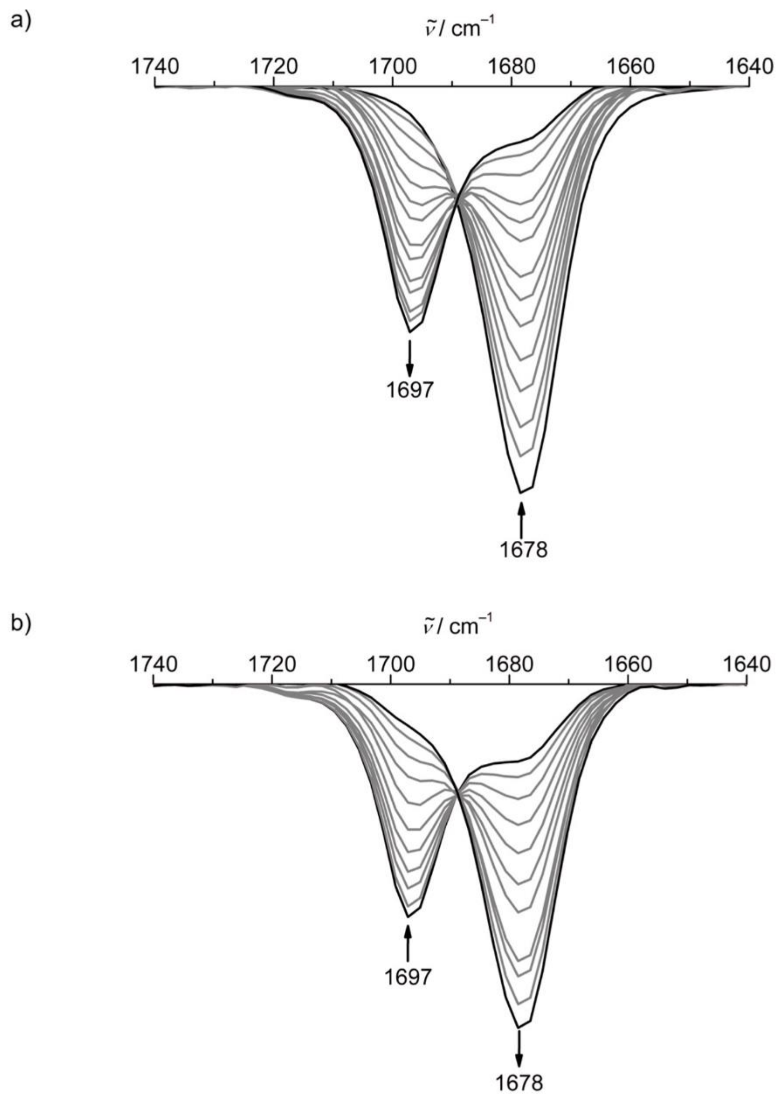

Figure S41. IR spectra (CO region) of **2[PF$_6$]$_2$** in acetonitrile, 0.1 M [^{n}Bu$_4$N][PF$_6$] a) during oxidation **2**$^{2+}$ $\rightarrow$ **2**$^{3+}$ and b) during re-reduction **2**$^{3+}$ $\rightarrow$ **2**$^{2+}$.

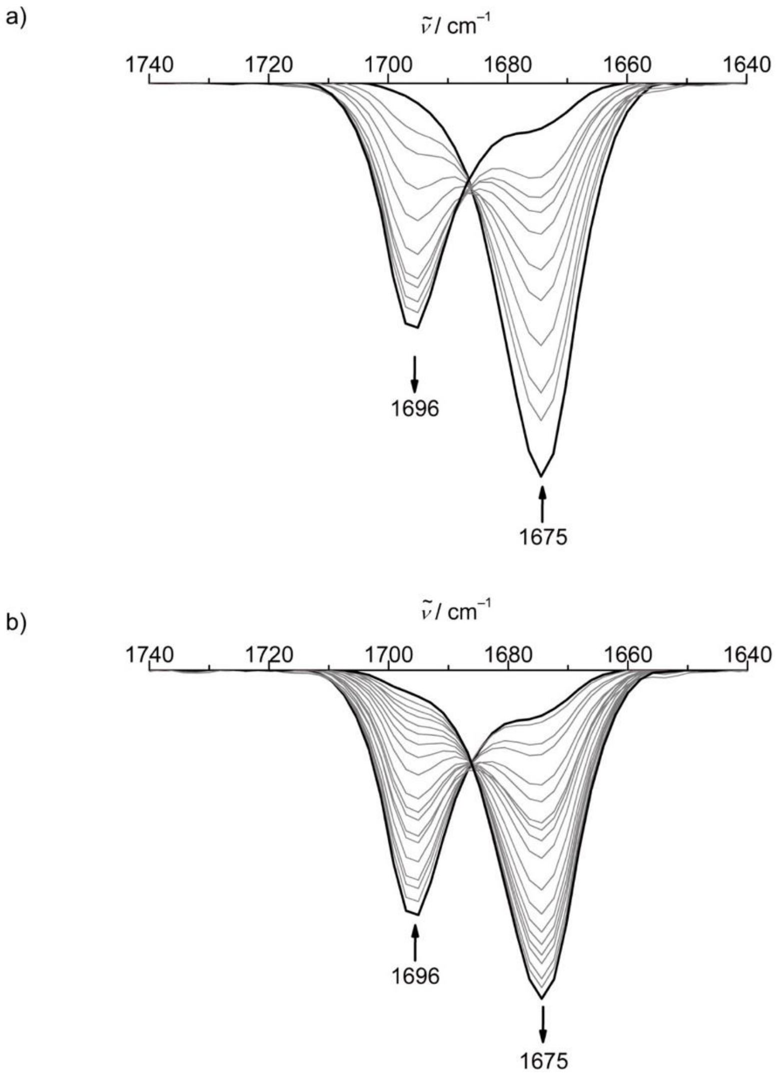

Figure S42. IR spectra (CO region) of **3[PF$_6$]$_2$** in acetonitrile, 0.1 M [^{n}Bu$_4$N][PF$_6$] a) during oxidation **3^{2+}** $\rightarrow$ **3^{3+}** and b) during re-reduction **3^{3+}** $\rightarrow$ **3^{2+}**.

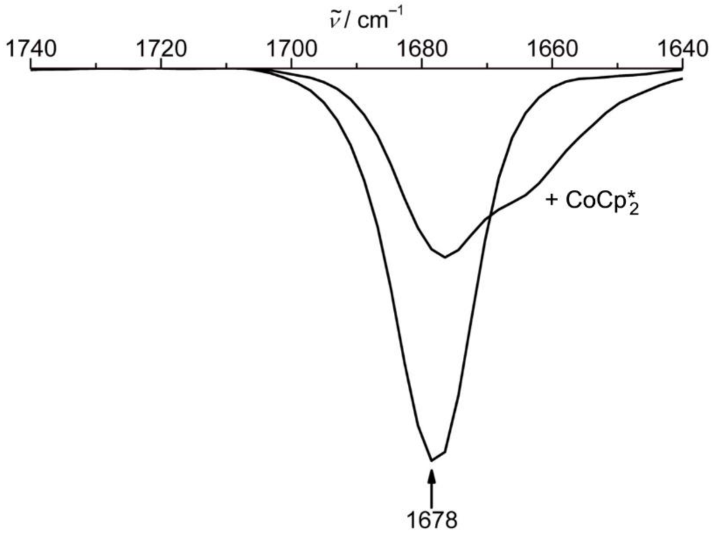

Figure S43. IR spectra (CO region) of **2[PF$_6$]$_2$** in acetonitrile before and after reduction **2^{2+}** $\rightarrow$ **2^{+}** with 1 eq. of decamethylcobaltocene.

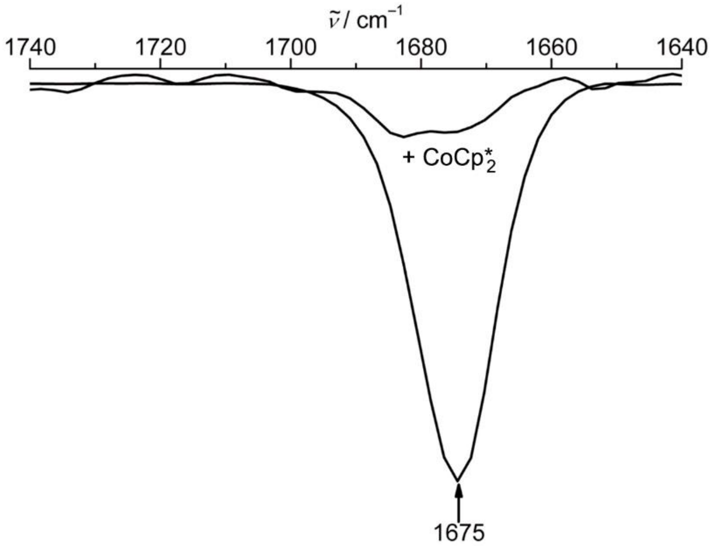

Figure S44. IR spectra (CO region) of **3[PF$_6$]$_2$** in acetonitrile before and after reduction **3^{2+}** $\rightarrow$ **3^{+}** with 1 eq. of decamethylcobaltocene.

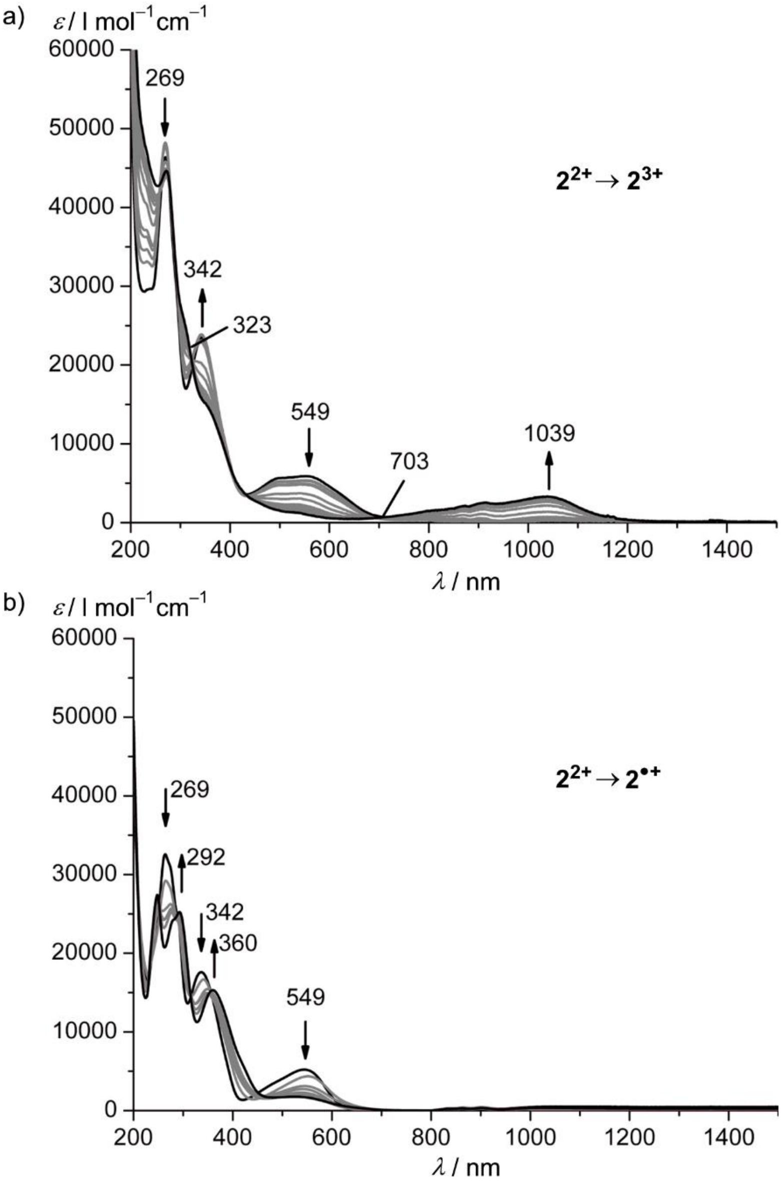

Figure S45. UV/Vis/NIR spectra of **2[PF$_6$]$_2$** in acetonitrile with 0.1 M [^{n}Bu$_4$N][PF$_6$] at 298 K collected during a) electrochemical oxidation and b) electrochemical reduction.

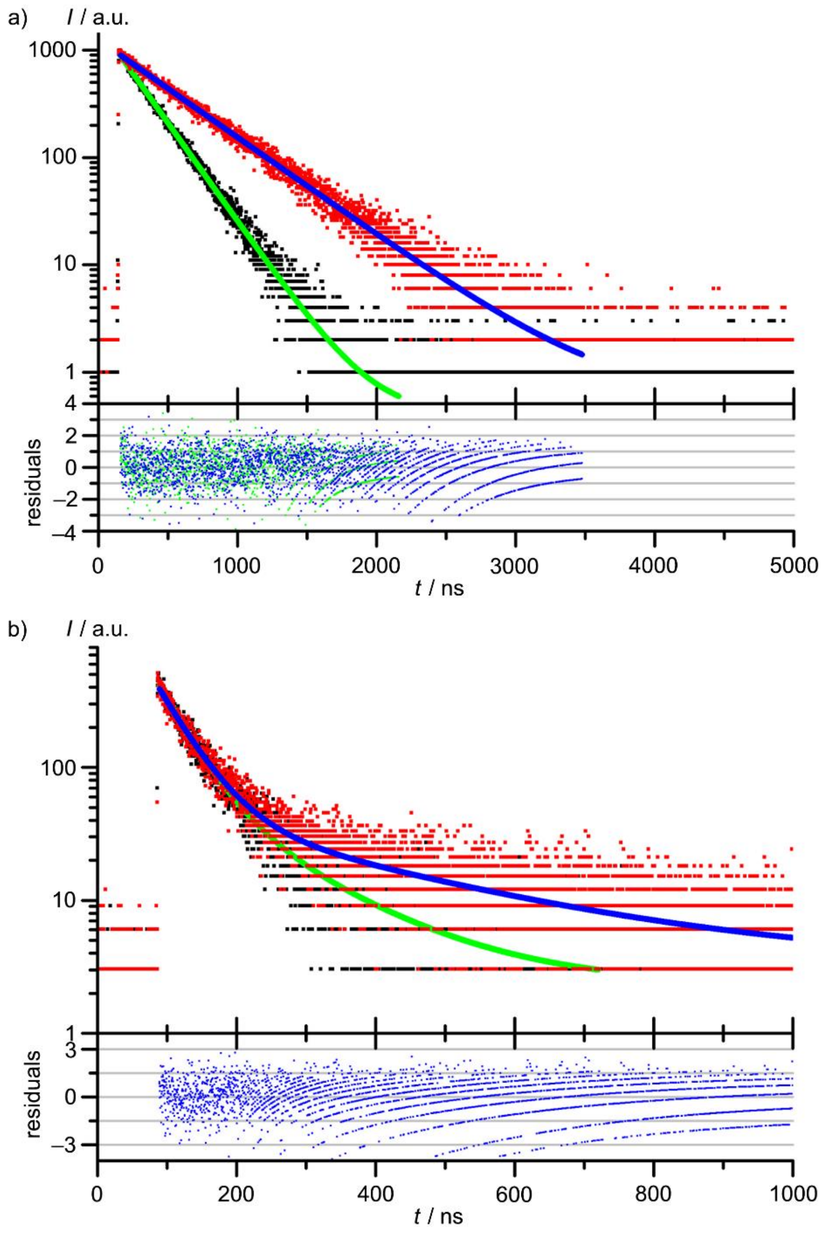

Figure S46. a) Luminescence decay of **2[PF$_6$]$_2$** in acetonitrile (λ_{exc} = 544 nm, λ_{em} = 709 nm) under argon (red) and air-saturated (black) with monoexponential fit functions (blue: τ = 477 ns; green: τ = 237 ns) and b) luminescence decay of **3[PF$_6$]$_2$** in acetonitrile (λ_{exc} = 542 nm, λ_{em} = 755 nm) under argon (red) and air-saturated (black) with biexponential fit functions (blue: τ_1 = 45 ns and τ_2 = 294 ns; green: τ_1 = 41 ns and τ_2 = 137 ns).

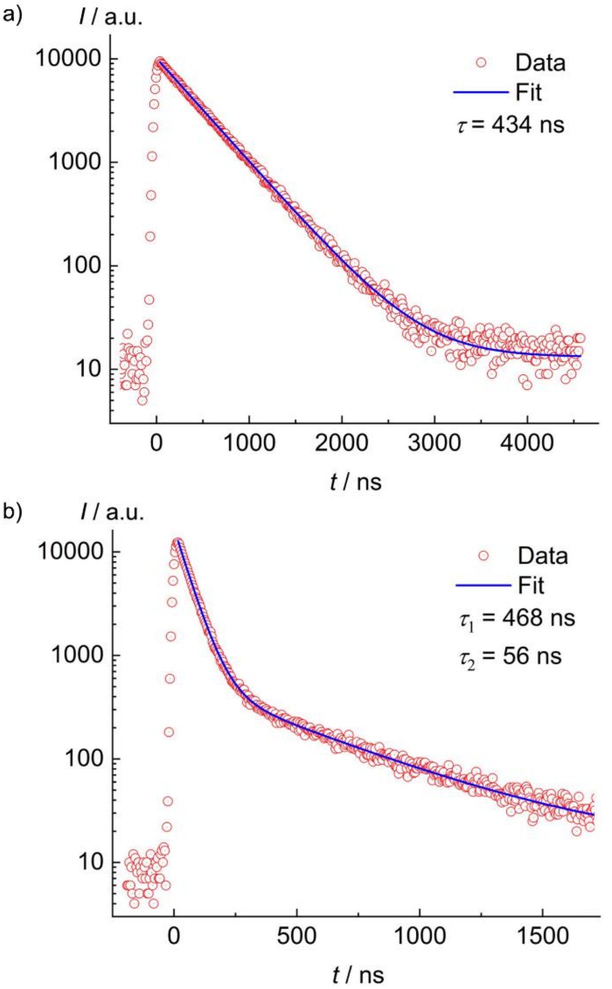

Figure S47. a) Streak camera measurement of **2[PF$_6$]$_2$** in acetonitrile (λ_{exc} = 540 nm), emission data integrated from 590 – 900 nm; monoexponential fit function with τ = 434 ns (blue) and b) streak camera measurement of **3[PF$_6$]$_2$** in acetonitrile (λ_{exc} = 540 nm), emission data integrated from 620 – 930 nm; biexponential fit function with τ_1 = 468 ns and τ_2 = 56 ns (blue).

a)

b)

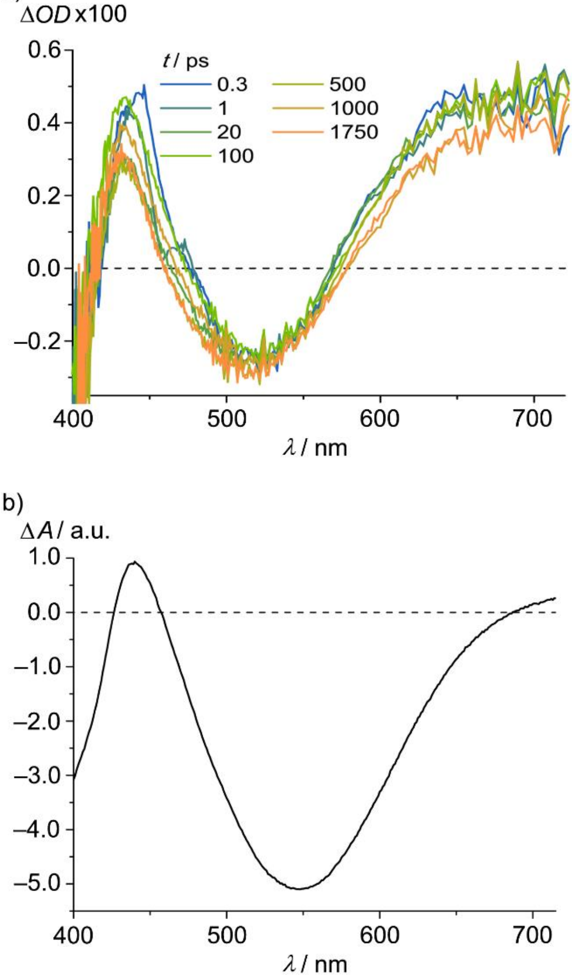

Figure S48. fs-Transient absorption spectra of **3[PF$_6$]$_2$** in acetonitrile at different delay times after excitation at 540 nm at 293 K (magic angle) and b) estimated difference spectrum derived from absorption spectra of $\mathbf{3}^{2+}$, $\mathbf{3}^{3+}$ and $\mathbf{3}^{\bullet+}$, prepared electrochemically in acetonitrile with 0.1 M [^{n}Bu$_4$N][PF$_6$] at 298 K by "scaled [½($\mathbf{3}^{3+}$ + $\mathbf{3}^{\bullet+}$) – $\mathbf{3}^{2+}$]".

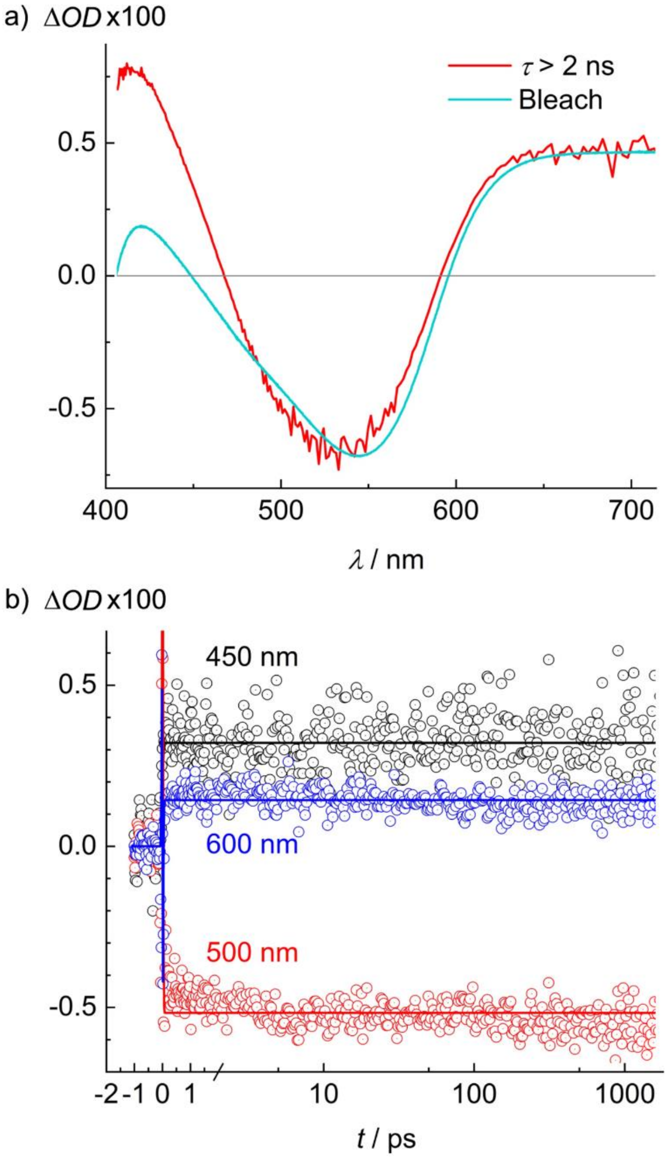

Figure S49. a) Amplitude spectrum (red) for **$2[PF_6]_2$** in acetonitrile obtained by a global fit of a long living component to the transient absorption data and compared to the bleach (cyan, scaled and vertically shifted negative ground state absorption). b) Time traces of the transient absorption of **$2[PF_6]_2$** at the detection wavelengths 450 nm (black), 500 nm (red), and 600 nm (blue) together with the time curves resulting from the global fit.

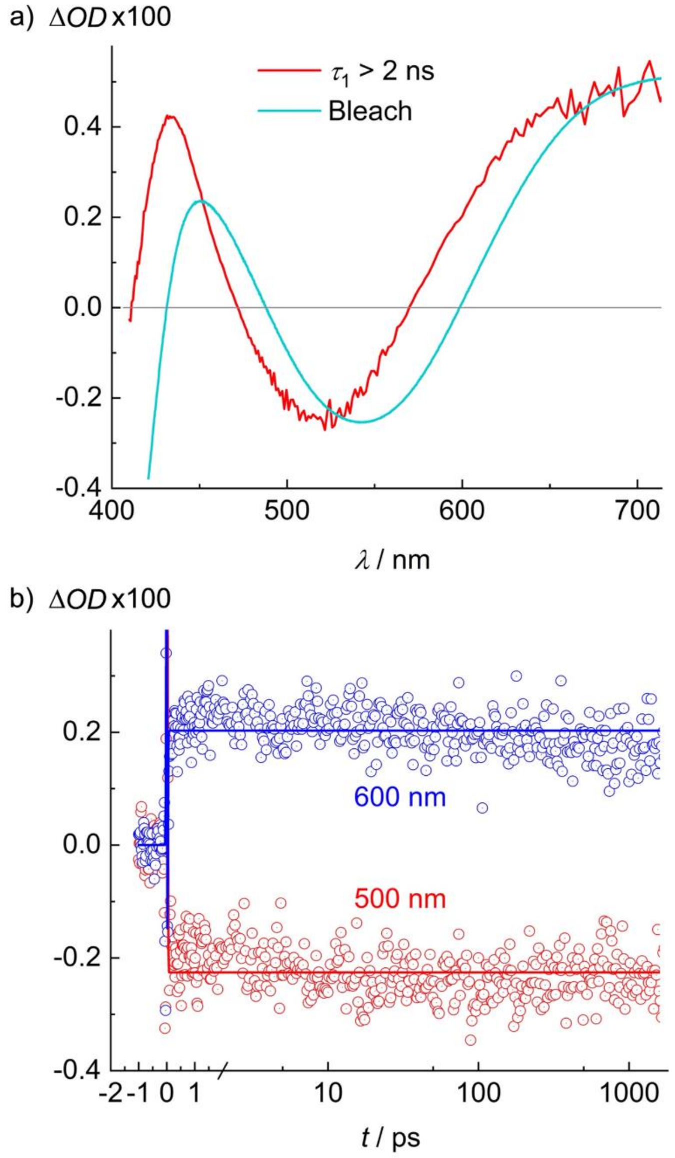

Figure S50. a) Amplitude spectrum (red) for **3[PF$_6$]$_2$** in acetonitrile obtained by a global fit of a long living component to the transient absorption data and compared to the bleach (cyan, scaled and vertically shifted negative ground state absorption). b) Time traces of the transient absorption of **3[PF$_6$]$_2$** at the detection wavelengths 500 nm (red) and 600 nm (blue) together with the time curves resulting from the global fit.

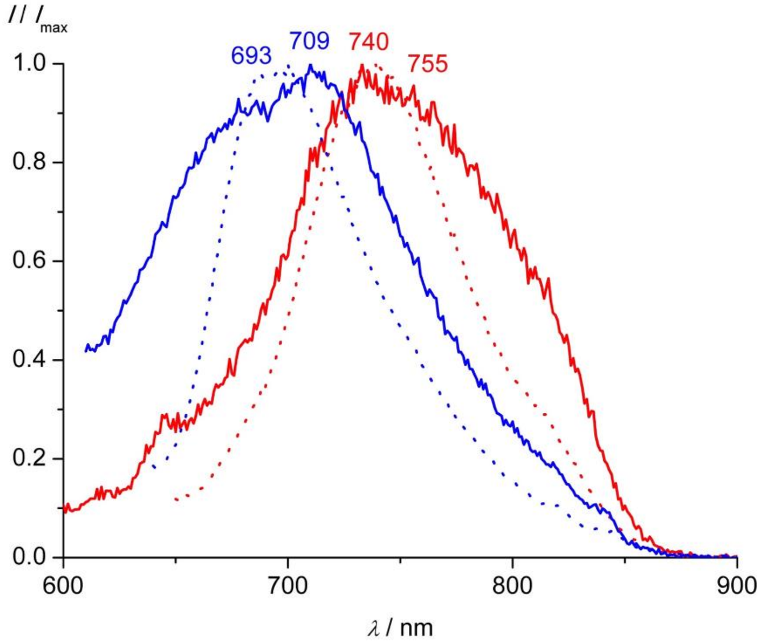

Figure S51. Normalized emission spectra of **2[PF6]2** (blue; λ_{exc} = 549 nm) and **3[PF6]2** (red; λ_{exc} = 541 nm) in butyronitrile at 298 K (solid lines) and at 77 K (dotted lines).

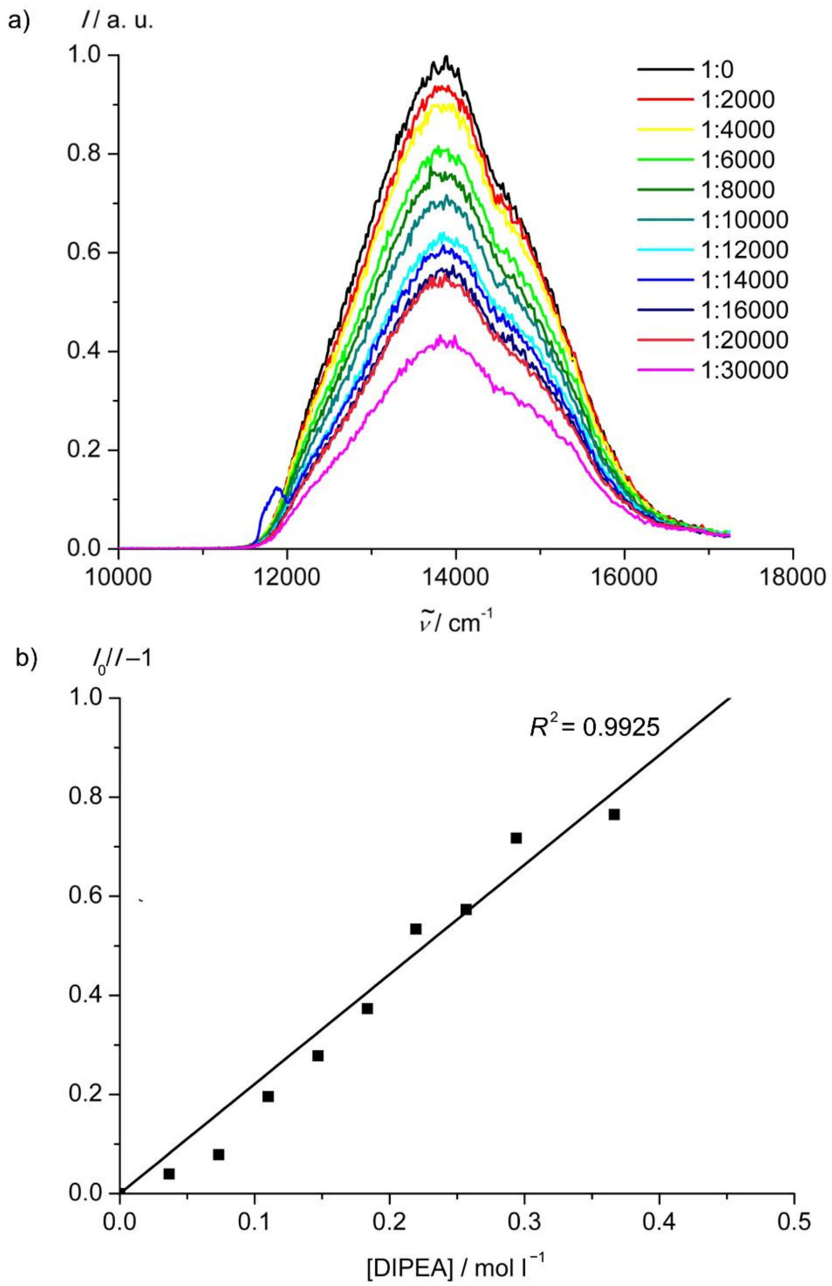

Figure S52. a) Emission spectra of of **$2[PF_6]_2$** with increasing concentration of DIPEA in dry and deaerated DMF at 298 K (λ_{exc} = 555 nm) and b) Stern-Volmer plot for phosphorescence quenching of **$2[PF_6]_2$** with DIPEA in in dry and deaerated DMF at 298 K (K_{SV} = 2.2 M^{-1}).

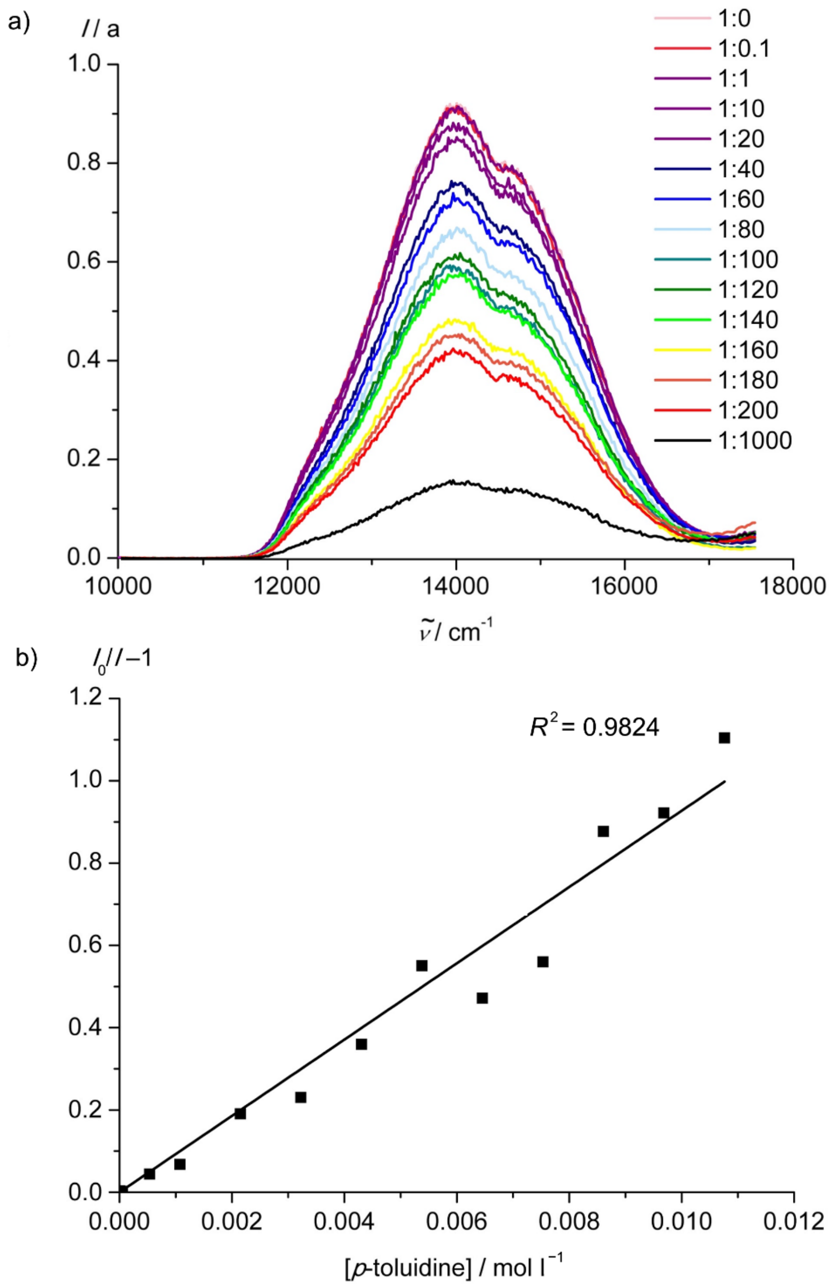

Figure S53. a) Emission spectra of of **2[PF$_6$]$_2$** with increasing concentration of *p*-toluidine in dry and deaerated CH_3CN at 298 K (λ_{exc} = 546 nm) and b) Stern-Volmer plot for phosphorescence quenching of **2[PF$_6$]$_2$** with *p*-toluidine in dry and deaerated CH_3CN at 298 K (K_{SV} = 93 M^{-1}).

a)

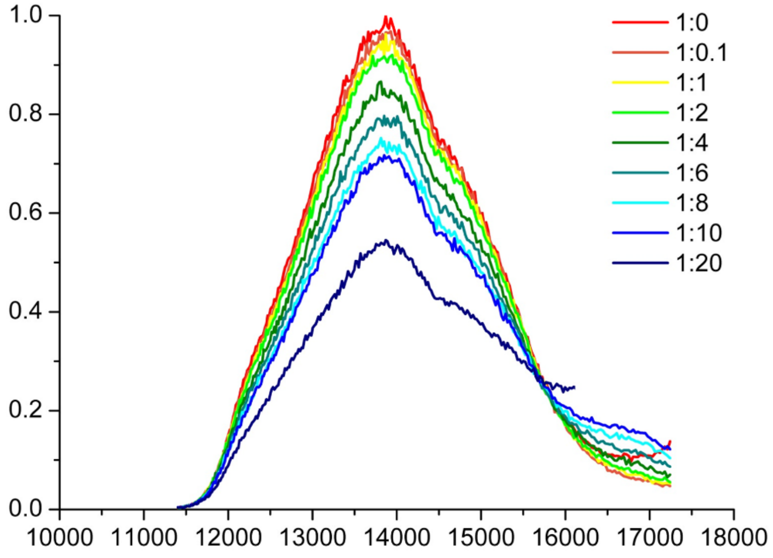

b)

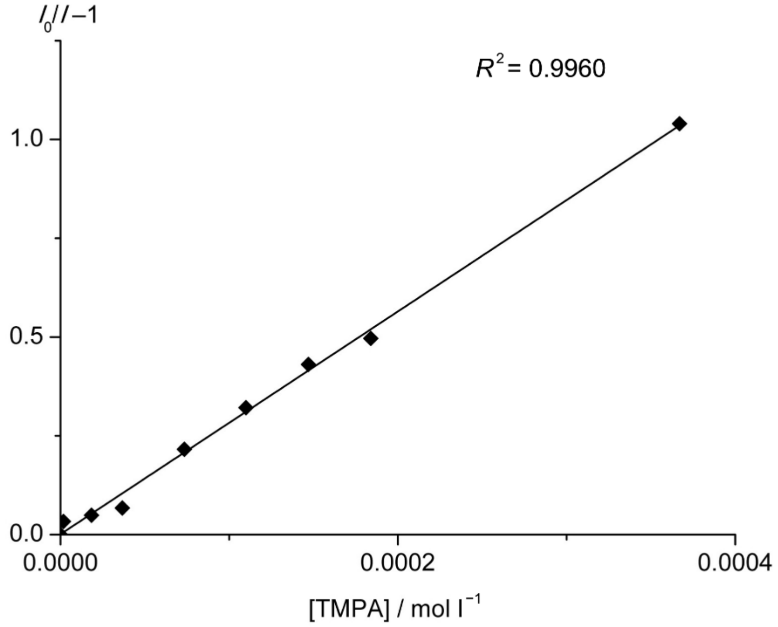

Figure S54. a) Emission spectra of of **2[PF$_6$]$_2$** with increasing concentration of TMPA in in dry and deaerated DMF at 298 K (λ_{exc} = 555 nm) and b) Stern-Volmer plot for phosphorescence quenching of **2[PF$_6$]$_2$** with TMPA in in dry and deaerated DMF at 298 K (K_{SV} = 2819 M^{-1}).

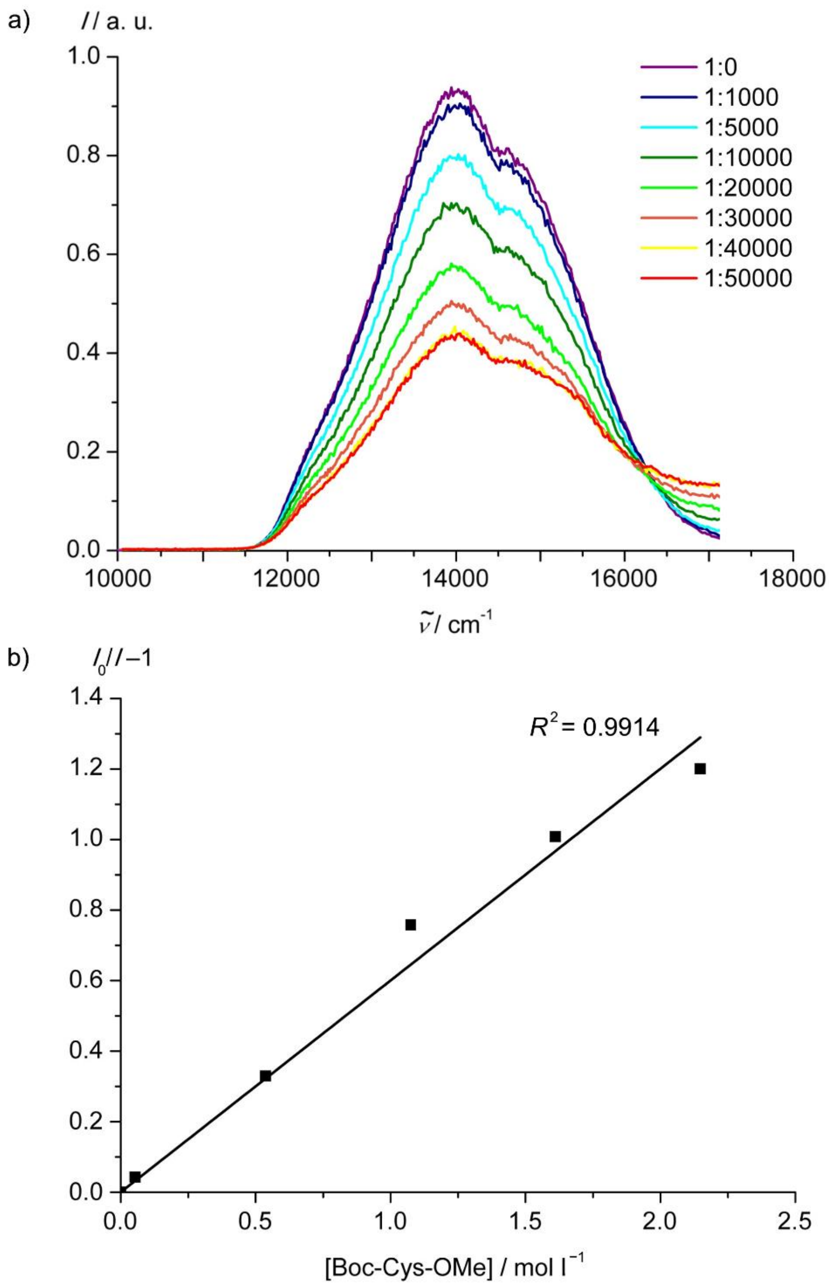

Figure S55. a) Emission spectra of of **2[PF$_6$]$_2$** with increasing concentration of *N*-Boc-cysteine methyl ester in in dry and deaerated CH_3CN at 298 K (λ_{exc} = 546 nm) and b) Stern-Volmer plot for phosphorescence quenching of **2[PF$_6$]$_2$** with *N*-Boc-cysteine methyl ester in in dry and deaerated CH_3CN at 298 K (K_{SV} = 0.60 M^{-1}).

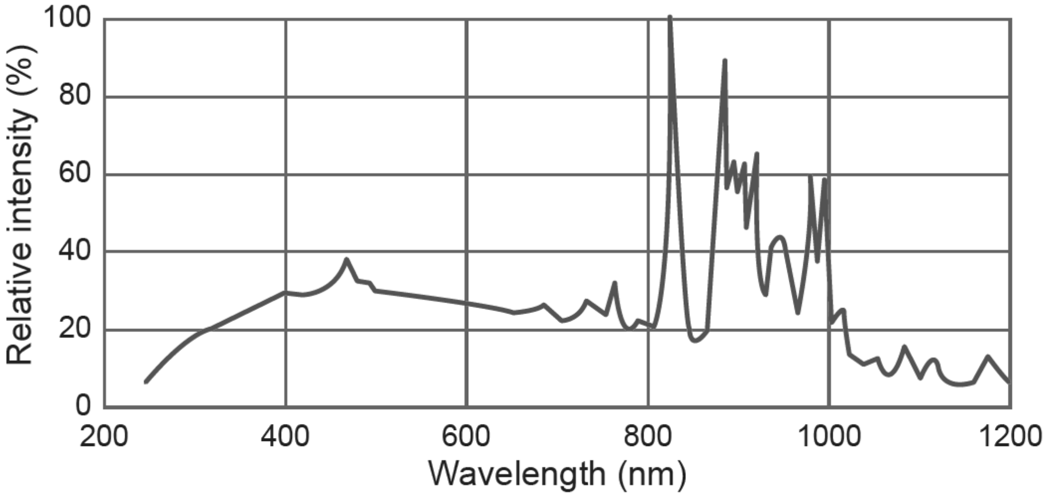

Figure S56. Spectral irradiance of the employed light source Asahi Spectra Max-303 Xenon Light Source (300 W).

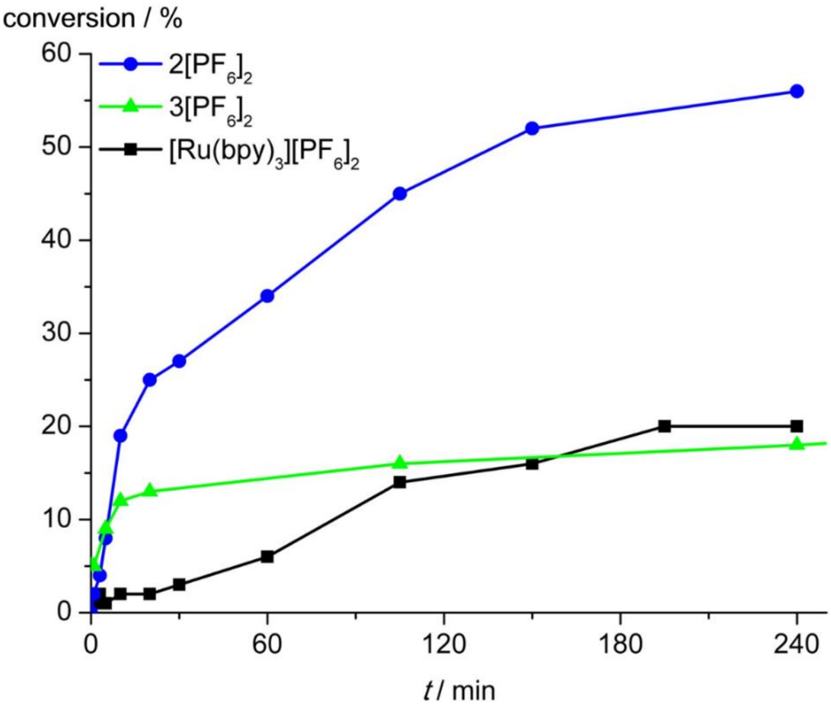

Figure S57. Conversion vs. time traces of the sensitized thiol-ene reaction between *N*-Boc cysteine methyl ester and allyl alcohol using **2[PF$_6$]$_2$** (blue), **3[PF$_6$]$_2$** (green) and [Ru(bpy)$_3$][PF$_6$]$_2$ (black) as sensitizer and *p*-toluidine as mediator in acetonitrile (λ_{exc} = 560±5 nm).

Table S1. Cartesian coordinates of DFT optimized geometry of cpmp (^{1}GS)

6	-2.129222000	3.161485000	-4.261733000
6	-2.912723000	4.023627000	-3.513729000
6	-3.417422000	3.584081000	-2.284910000
7	-3.220491000	2.350701000	-1.827717000
6	-2.470421000	1.519610000	-2.564419000
6	-1.884834000	1.882677000	-3.772799000
1	-1.725574000	3.475095000	-5.215567000
1	-3.146396000	5.019005000	-3.865845000
1	-1.269668000	1.176165000	-4.311869000
7	-4.199518000	4.449003000	-1.485082000
8	-1.090023000	-0.356982000	-2.194675000
6	-1.948588000	7.269119000	-0.779719000
6	-2.893001000	8.193492000	-0.323763000
6	-4.214145000	7.784916000	-0.277227000
7	-4.640466000	6.574772000	-0.667715000
6	-3.732602000	5.698076000	-1.120966000
6	-2.356226000	6.012146000	-1.172674000
1	-0.897263000	7.528409000	-0.815412000
1	-2.605801000	9.184478000	-0.000472000
1	-4.984036000	8.455871000	0.088657000
1	-1.633125000	5.278350000	-1.496487000
6	-5.593802000	4.083633000	-1.238704000
1	-6.268909000	4.667796000	-1.868109000
1	-5.707340000	3.027733000	-1.472215000
1	-5.858320000	4.259107000	-0.196729000
6	-3.219470000	-0.534500000	-1.181538000
7	-4.502612000	-0.309573000	-1.498917000
6	-2.820647000	-1.425339000	-0.185140000
1	-1.769095000	-1.575699000	0.018199000
6	-5.432005000	-0.977784000	-0.815842000
6	-3.800075000	-2.100256000	0.531440000
1	-6.462053000	-0.791423000	-1.099464000
1	-3.527513000	-2.789199000	1.320802000
6	-5.132607000	-1.873553000	0.209999000
1	-5.930491000	-2.376937000	0.739164000
6	-2.177199000	0.164633000	-1.995820000

Table S2. Cartesian coordinates of DFT optimized geometry of **2^{2+}** (^{1}GS).

6	1.570086000	-2.602123000	-0.160146000
6	1.748467000	-3.988079000	-0.146245000
6	0.656832000	-4.815102000	0.036276000
6	-0.604198000	-4.252699000	0.197025000
6	-0.714196000	-2.873414000	0.211790000
7	0.352813000	-2.056759000	0.051062000
1	2.728531000	-4.407224000	-0.312402000
1	-1.485715000	-4.861440000	0.331306000
6	1.511101000	0.995841000	-2.389047000
6	2.507249000	1.164453000	-3.323336000
6	3.635008000	0.352067000	-3.239724000
6	3.693494000	-0.622600000	-2.260925000
6	2.639823000	-0.749330000	-1.351778000
7	1.582312000	0.086117000	-1.391641000
1	4.445939000	0.455606000	-3.948881000
1	0.621680000	1.605874000	-2.409249000
1	2.404798000	1.921169000	-4.088100000
1	4.536123000	-1.294384000	-2.210655000
6	-2.253814000	-1.195970000	1.465738000
6	-3.336529000	-1.272076000	2.336672000
6	-3.463639000	-0.326924000	3.343387000
6	-2.491945000	0.659836000	3.453669000
6	-1.458176000	0.693476000	2.535121000
7	-1.342301000	-0.204377000	1.540256000
1	-4.293404000	-0.369190000	4.036162000
1	-4.049573000	-2.075768000	2.223680000
1	-2.533109000	1.408607000	4.232728000
1	-0.699928000	1.459852000	2.584296000
6	0.944139000	2.827463000	0.271426000
6	0.834125000	4.206947000	0.273267000
6	-0.423298000	4.771740000	0.093399000
6	-1.510522000	3.947100000	-0.122672000
6	-1.331872000	2.561584000	-0.148949000
7	-0.118970000	2.013006000	0.077848000
1	1.712342000	4.813676000	0.435597000
1	-2.486936000	4.368395000	-0.304676000
6	1.641565000	-0.775711000	2.555537000
6	2.652795000	-0.753153000	3.499065000
6	3.622993000	0.238742000	3.427670000
6	3.517401000	1.197942000	2.431909000
6	2.456330000	1.131703000	1.533918000
7	1.546250000	0.137154000	1.572122000
1	4.435488000	0.273277000	4.141118000
1	0.884077000	-1.544128000	2.574809000
1	2.677521000	-1.513594000	4.267428000
1	4.230315000	2.005412000	2.348569000
6	-2.382464000	0.726387000	-1.383320000
6	-3.422156000	0.612891000	-2.310193000
6	-3.348061000	-0.347381000	-3.301936000
6	-2.218687000	-1.158150000	-3.379788000
6	-1.237072000	-1.003170000	-2.427897000
7	-1.323998000	-0.108161000	-1.418589000
1	-4.147897000	-0.440748000	-4.025022000
1	-4.265788000	1.283736000	-2.263177000
1	-2.103565000	-1.903114000	-4.154215000
1	-0.347243000	-1.612711000	-2.443510000
7	2.664695000	-1.751075000	-0.371646000
6	-2.081492000	-2.295408000	0.463466000
8	-3.057378000	-2.841872000	-0.016714000
1	0.782759000	-5.889546000	0.031497000
1	-0.549309000	5.846154000	0.100409000
44	0.116566000	-0.022191000	0.080374000
7	-2.422810000	1.713471000	-0.389391000
6	2.305404000	2.245262000	0.544336000
8	3.291908000	2.797696000	0.093598000
6	-3.748437000	2.188850000	0.029192000
1	-4.191343000	2.902908000	-0.668207000
1	-4.404452000	1.326806000	0.123103000

1	-3.655202000	2.657245000	1.006084000
6	3.983792000	-2.232818000	0.060636000
1	3.874696000	-2.715190000	1.028985000
1	4.436941000	-2.936820000	-0.640571000
1	4.638048000	-1.372244000	0.178141000

Table S3. Cartesian coordinates of DFT optimized geometry of **2^{2+}** (^{3}MLCT; spin density at 0.05 a.u.).

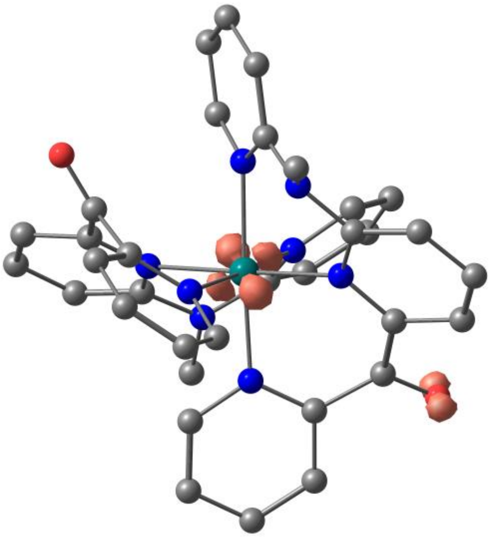

6	1.586066000	-2.583220000	-0.135360000
6	1.762784000	-3.972682000	-0.058389000
6	0.667589000	-4.786758000	0.114456000
6	-0.604749000	-4.220958000	0.219264000
6	-0.717076000	-2.850132000	0.188725000
7	0.359845000	-2.036752000	0.031963000
1	2.745301000	-4.398750000	-0.183041000
1	-1.486632000	-4.830119000	0.348254000
6	1.519044000	1.007270000	-2.369363000
6	2.528303000	1.196359000	-3.281183000
6	3.669306000	0.402097000	-3.179918000
6	3.731114000	-0.574587000	-2.206358000
6	2.659719000	-0.729364000	-1.321390000
7	1.592375000	0.088179000	-1.378734000
1	4.490000000	0.523097000	-3.874661000
1	0.622662000	1.605126000	-2.396697000
1	2.428865000	1.956921000	-4.042152000
1	4.584428000	-1.230956000	-2.146082000
6	-2.285901000	-1.185215000	1.413596000
6	-3.381771000	-1.259654000	2.263168000
6	-3.516708000	-0.317116000	3.273800000
6	-2.539950000	0.658837000	3.411536000
6	-1.487222000	0.690706000	2.513349000
7	-1.370197000	-0.202919000	1.517365000
1	-4.359484000	-0.356456000	3.950624000
1	-4.098400000	-2.057581000	2.133934000
1	-2.587473000	1.400548000	4.196520000
1	-0.721517000	1.448021000	2.580833000
6	0.979748000	2.842279000	0.279410000
6	0.830212000	4.230498000	0.178066000
6	-0.414971000	4.776048000	-0.046806000
6	-1.521047000	3.942249000	-0.206666000
6	-1.331774000	2.571534000	-0.167523000
7	-0.109276000	2.025773000	0.075881000
1	1.702339000	4.848099000	0.325302000
1	-2.495610000	4.352787000	-0.415752000
6	1.622601000	-0.776016000	2.524833000
6	2.683533000	-0.857738000	3.393172000
6	3.716512000	0.083187000	3.269177000
6	3.602832000	1.088027000	2.336591000
6	2.468310000	1.163358000	1.503094000
7	1.531264000	0.173999000	1.566957000

1	4.582234000	0.033628000	3.917170000
1	0.817306000	-1.493762000	2.569765000
1	2.712910000	-1.643840000	4.134136000
1	4.355394000	1.855751000	2.237398000
6	-2.405291000	0.731978000	-1.365534000
6	-3.465897000	0.584708000	-2.264950000
6	-3.372913000	-0.354624000	-3.272723000
6	-2.212624000	-1.117869000	-3.395116000
6	-1.217946000	-0.944254000	-2.463475000
7	-1.325283000	-0.069061000	-1.437160000
1	-4.185119000	-0.470093000	-3.978408000
1	-4.334495000	1.219201000	-2.188079000
1	-2.087988000	-1.844498000	-4.184902000
1	-0.309575000	-1.524628000	-2.503961000
7	2.680233000	-1.760030000	-0.373529000
6	-2.094280000	-2.272571000	0.400680000
8	-3.052656000	-2.806891000	-0.117289000
1	0.790554000	-5.861094000	0.142692000
1	-0.538802000	5.849597000	-0.107349000
44	0.119044000	0.001700000	0.077920000
7	-2.429200000	1.712575000	-0.374643000
6	2.304306000	2.335869000	0.649501000
8	3.312352000	3.070617000	0.417922000
6	-3.749690000	2.201102000	0.050451000
1	-4.198535000	2.895764000	-0.661599000
1	-4.405465000	1.342836000	0.176798000
1	-3.639410000	2.697250000	1.010795000
6	4.003691000	-2.245932000	0.057854000
1	3.891858000	-2.744244000	1.016374000
1	4.455020000	-2.931476000	-0.660255000
1	4.648964000	-1.382508000	0.195258000

Table S4. Cartesian coordinates of DFT optimized geometry of $\mathbf{2}^{2+}$ (^{3}MC; spin density at 0.05 a.u.).

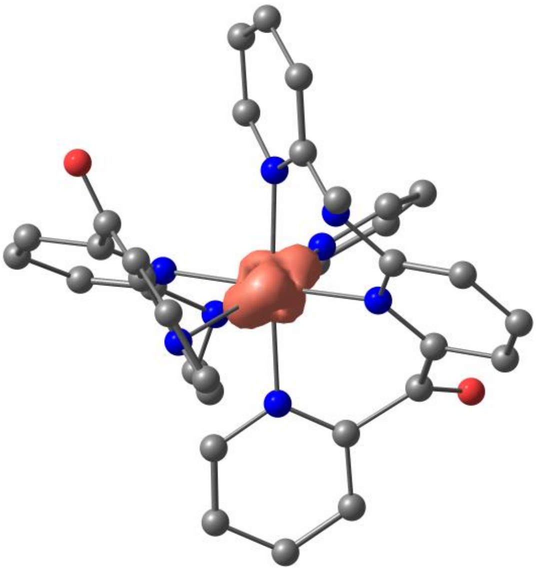

6	1.591740000	-2.648073000	-0.202235000
6	1.770057000	-4.035664000	-0.168134000
6	0.703019000	-4.862297000	0.109305000
6	-0.545738000	-4.296286000	0.338071000
6	-0.667719000	-2.920017000	0.308842000
7	0.381519000	-2.096282000	0.053265000
1	2.739575000	-4.454625000	-0.386567000
1	-1.412275000	-4.904636000	0.550457000
6	1.818895000	0.872235000	-2.619616000
6	2.879632000	0.979032000	-3.497454000
6	3.946039000	0.096699000	-3.338360000
6	3.905979000	-0.859960000	-2.337186000
6	2.794197000	-0.897959000	-1.487030000
7	1.794243000	-0.027030000	-1.626817000
1	4.798788000	0.141584000	-4.003656000

1	0.964594000	1.535480000	-2.682507000
1	2.874414000	1.731361000	-4.273730000
1	4.712467000	-1.568922000	-2.227982000
6	-2.343557000	-1.340959000	1.574750000
6	-3.656778000	-1.028715000	1.915527000
6	-3.876232000	-0.058173000	2.885832000
6	-2.783028000	0.568845000	3.473523000
6	-1.505455000	0.206594000	3.064491000
7	-1.287606000	-0.731651000	2.140242000
1	-4.883240000	0.214241000	3.173516000
1	-4.475616000	-1.535668000	1.425680000
1	-2.912825000	1.330908000	4.229858000
1	-0.628495000	0.688533000	3.478377000
6	0.847664000	2.906372000	0.331025000
6	0.695047000	4.276840000	0.385814000
6	-0.590698000	4.801208000	0.291587000
6	-1.660166000	3.950427000	0.100154000
6	-1.438746000	2.572543000	0.004835000
7	-0.196758000	2.062437000	0.158560000
1	1.559871000	4.909486000	0.518035000
1	-2.655051000	4.349044000	-0.016321000
6	1.878403000	-0.710476000	2.490572000
6	2.947203000	-0.644411000	3.367155000
6	3.848761000	0.406429000	3.256969000
6	3.626591000	1.373571000	2.287391000
6	2.512836000	1.258768000	1.462671000
7	1.664472000	0.215515000	1.539816000
1	4.701791000	0.477576000	3.918299000
1	1.162793000	-1.516941000	2.540542000
1	3.065419000	-1.414067000	4.117408000
1	4.287325000	2.220158000	2.170922000
6	-2.477513000	0.743107000	-1.257026000
6	-3.545737000	0.624432000	-2.149718000
6	-3.479429000	-0.310712000	-3.165860000
6	-2.339537000	-1.100290000	-3.298014000
6	-1.330316000	-0.943438000	-2.377565000
7	-1.403453000	-0.066439000	-1.351338000
1	-4.299503000	-0.403118000	-3.866083000
1	-4.402300000	1.273856000	-2.064201000
1	-2.237059000	-1.826472000	-4.091574000
1	-0.426876000	-1.530651000	-2.427849000
7	2.716180000	-1.834123000	-0.436791000
6	-2.068903000	-2.388030000	0.545478000
8	-2.985397000	-2.916601000	-0.057088000
1	0.833236000	-5.936233000	0.126803000
1	-0.752331000	5.869701000	0.342769000
44	0.103536000	0.014811000	0.119251000
7	-2.512826000	1.697291000	-0.223494000
6	2.244138000	2.358599000	0.481599000
8	3.166194000	2.925305000	-0.069833000
6	-3.846018000	2.170793000	0.183360000
1	-4.274432000	2.891353000	-0.515784000
1	-4.503163000	1.309686000	0.259291000
1	-3.766988000	2.625173000	1.166872000
6	3.997838000	-2.334470000	0.084527000
1	3.825960000	-2.778294000	1.061649000
1	4.471054000	-3.074243000	-0.564420000
1	4.668832000	-1.486549000	0.204843000

Table S5. Cartesian coordinates of DFT optimized geometry of **3**$^{2+}$ (^{1}GS).

6	1.424306000	-2.566881000	-0.261222000
6	1.597366000	-3.946103000	-0.290281000
6	0.499131000	-4.761837000	-0.076064000
6	-0.741614000	-4.198499000	0.172246000
6	-0.855880000	-2.812237000	0.202292000
7	0.212398000	-2.011350000	-0.021505000
1	2.564780000	-4.370318000	-0.507019000
1	-1.599460000	-4.820830000	0.371259000
6	1.408078000	1.057944000	-2.457486000
6	2.407828000	1.226626000	-3.388035000
6	3.528694000	0.403929000	-3.308585000
6	3.575040000	-0.580816000	-2.339364000
6	2.517114000	-0.707546000	-1.433599000
7	1.468016000	0.139520000	-1.467183000
1	4.342970000	0.507558000	-4.014015000
1	0.523349000	1.674843000	-2.474852000
1	2.314315000	1.991399000	-4.146034000
1	4.412176000	-1.259867000	-2.293153000
6	-2.279155000	-1.243155000	1.446676000
6	-3.316682000	-1.364011000	2.377280000
6	-3.474038000	-0.397042000	3.352987000
6	-2.572580000	0.662315000	3.410944000
6	-1.572838000	0.719329000	2.465358000
7	-1.440544000	-0.189839000	1.476818000
1	-4.273871000	-0.485094000	4.076757000
1	-3.979169000	-2.214716000	2.344002000
1	-2.642254000	1.430165000	4.168621000
1	-0.846918000	1.518008000	2.472593000
6	0.874439000	2.867585000	0.214827000
6	0.784838000	4.249782000	0.226739000
6	-0.461977000	4.835118000	0.045957000
6	-1.559828000	4.026739000	-0.182983000
6	-1.402613000	2.639169000	-0.216050000
7	-0.199170000	2.068660000	0.013418000
1	1.671844000	4.841396000	0.397108000
1	-2.529011000	4.463134000	-0.368809000
6	1.521800000	-0.757305000	2.477189000
6	2.538168000	-0.758636000	3.415034000
6	3.526033000	0.216393000	3.345112000
6	3.431025000	1.183104000	2.356148000
6	2.364071000	1.141151000	1.462592000
7	1.437561000	0.161314000	1.497242000
1	4.342786000	0.231829000	4.054410000
1	0.751879000	-1.512784000	2.495020000
1	2.553132000	-1.524197000	4.178696000
1	4.156506000	1.979508000	2.274190000
6	-2.472969000	0.827330000	-1.466215000
6	-3.511704000	0.734212000	-2.397502000
6	-3.454380000	-0.230436000	-3.386975000
6	-2.343124000	-1.065954000	-3.454589000
6	-1.359653000	-0.924031000	-2.500568000
7	-1.430206000	-0.022662000	-1.499492000
1	-4.253245000	-0.309908000	-4.112915000
1	-4.341646000	1.422486000	-2.354400000
1	-2.241314000	-1.818237000	-4.224034000
1	-0.482097000	-1.551540000	-2.509052000
7	2.527591000	-1.716862000	-0.463731000
7	-2.104173000	-2.213841000	0.447455000
6	3.841093000	-2.220071000	-0.040530000
1	4.287181000	-2.918506000	-0.752189000
1	4.506624000	-1.369833000	0.090701000
1	3.726206000	-2.717906000	0.919168000
6	-3.296670000	-2.964161000	0.036095000
1	-3.583782000	-3.745251000	0.743896000
1	-4.120412000	-2.261761000	-0.072694000
1	-3.100375000	-3.417799000	-0.932336000
1	0.610018000	-5.837996000	-0.101477000
1	-0.572343000	5.911200000	0.061445000

44	0.006865000	0.032135000	0.004820000
7	-2.504681000	1.808712000	-0.464012000
6	2.226994000	2.266625000	0.485821000
8	3.221807000	2.816060000	0.046854000
6	-3.823881000	2.298991000	-0.047630000
1	-4.257262000	3.021713000	-0.742901000
1	-4.491627000	1.445043000	0.041316000
1	-3.727968000	2.762193000	0.931542000

Table S6. Cartesian coordinates of DFT optimized geometry of **3^{2+}** (^{3}MLCT; spin density at 0.05 a.u.).

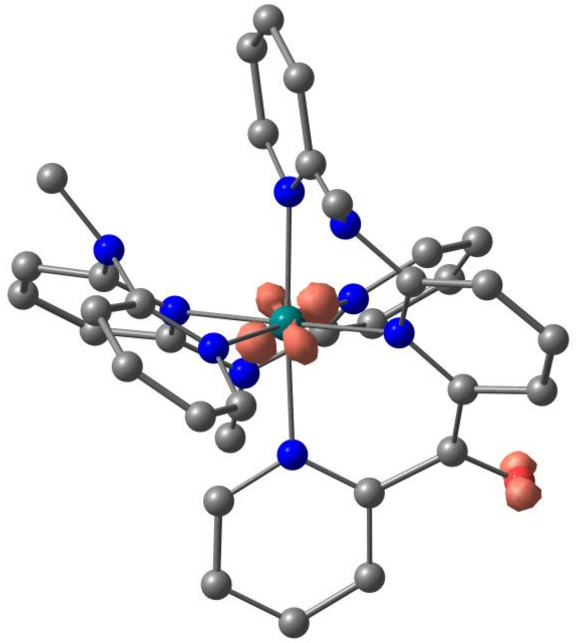

6	1.421907000	-2.567610000	-0.253335000
6	1.567575000	-3.948041000	-0.271957000
6	0.450071000	-4.742923000	-0.080445000
6	-0.788723000	-4.164867000	0.139259000
6	-0.883900000	-2.779790000	0.177445000
7	0.207898000	-1.993877000	-0.025722000
1	2.529530000	-4.391284000	-0.472832000
1	-1.657122000	-4.776779000	0.321326000
6	1.447501000	1.061622000	-2.424863000
6	2.460288000	1.238014000	-3.337310000
6	3.579484000	0.413119000	-3.249140000
6	3.616350000	-0.579353000	-2.288898000
6	2.544630000	-0.715553000	-1.400912000
7	1.500572000	0.130027000	-1.447059000
1	4.402822000	0.523456000	-3.942729000
1	0.565042000	1.681115000	-2.443316000
1	2.378976000	2.011120000	-4.087849000
1	4.453552000	-1.257152000	-2.236712000
6	-2.311070000	-1.192517000	1.394055000
6	-3.403639000	-1.248096000	2.267555000
6	-3.532411000	-0.286553000	3.250379000
6	-2.554566000	0.698961000	3.382389000
6	-1.513182000	0.706633000	2.486995000
7	-1.417114000	-0.189534000	1.477931000
1	-4.370465000	-0.325811000	3.933683000
1	-4.122657000	-2.047962000	2.192125000
1	-2.603825000	1.449986000	4.157712000
1	-0.730879000	1.447382000	2.540169000
6	0.883190000	2.887730000	0.240548000
6	0.725670000	4.277272000	0.154128000
6	-0.523412000	4.820063000	-0.051701000
6	-1.627796000	3.983116000	-0.209361000
6	-1.430397000	2.612330000	-0.191122000
7	-0.204188000	2.067093000	0.033973000
1	1.596768000	4.896729000	0.299265000

1	-2.605847000	4.391533000	-0.405720000
6	1.581611000	-0.757672000	2.435559000
6	2.641053000	-0.827562000	3.309489000
6	3.650750000	0.137930000	3.206428000
6	3.517369000	1.149510000	2.283705000
6	2.386537000	1.206440000	1.442967000
7	1.469255000	0.202359000	1.493189000
1	4.513600000	0.101594000	3.859205000
1	0.795472000	-1.496770000	2.466324000
1	2.683856000	-1.624365000	4.038527000
1	4.251625000	1.936119000	2.196497000
6	-2.508386000	0.773334000	-1.389681000
6	-3.590217000	0.601183000	-2.262422000
6	-3.503520000	-0.339555000	-3.268476000
6	-2.331661000	-1.082374000	-3.416299000
6	-1.318177000	-0.885531000	-2.510434000
7	-1.416102000	-0.007893000	-1.486328000
1	-4.329584000	-0.472467000	-3.954795000
1	-4.467902000	1.220791000	-2.168644000
1	-2.213657000	-1.809437000	-4.206783000
1	-0.399432000	-1.447243000	-2.570167000
7	2.534752000	-1.743341000	-0.450686000
7	-2.126535000	-2.173742000	0.414621000
6	3.841805000	-2.272269000	-0.028561000
1	4.281870000	-2.953588000	-0.759080000
1	4.511687000	-1.431051000	0.132115000
1	3.712007000	-2.792446000	0.916226000
6	-3.322728000	-2.916766000	-0.015467000
1	-3.626380000	-3.682810000	0.699743000
1	-4.134196000	-2.205552000	-0.149028000
1	-3.109624000	-3.382531000	-0.973563000
1	0.544928000	-5.820563000	-0.102221000
1	-0.652507000	5.893704000	-0.099516000
44	0.020893000	0.042497000	0.016594000
7	-2.528977000	1.757654000	-0.406379000
6	2.210723000	2.386872000	0.597578000
8	3.212482000	3.134472000	0.377117000
6	-3.846813000	2.235256000	0.038111000
1	-4.311838000	2.923956000	-0.669346000
1	-4.493266000	1.371812000	0.176771000
1	-3.725715000	2.732740000	0.996030000

Table S7. Cartesian coordinates of DFT optimized geometry of $\mathbf{3^{2+}}$ (^{3}MC; spin density at 0.05 a.u.).

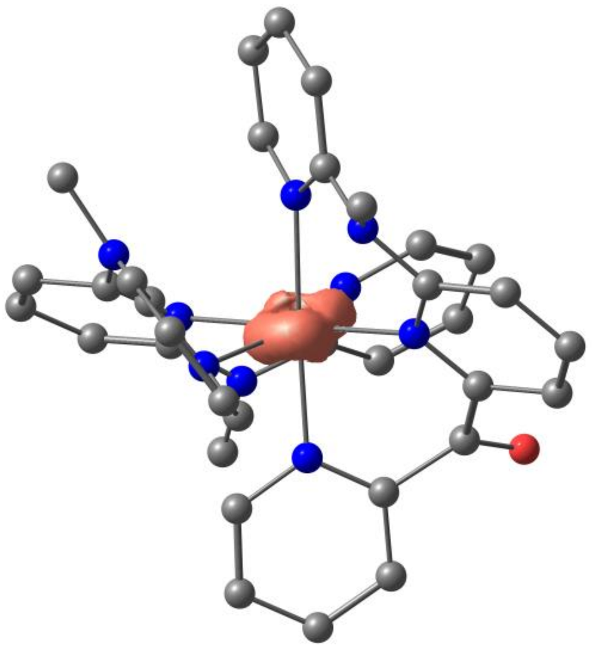

6	1.427243000	-2.642312000	-0.289440000
6	1.586899000	-4.024572000	-0.311301000

6	0.499151000	-4.834717000	-0.041099000
6	-0.726092000	-4.258606000	0.240474000
6	-0.839045000	-2.871095000	0.244292000
7	0.225437000	-2.070305000	-0.019066000
1	2.544646000	-4.454311000	-0.559586000
1	-1.580854000	-4.869417000	0.485527000
6	1.484947000	0.768527000	-2.685281000
6	2.636064000	1.214668000	-3.298189000
6	3.841956000	0.621495000	-2.925204000
6	3.850015000	-0.401882000	-1.993500000
6	2.634425000	-0.806230000	-1.423795000
7	1.487095000	-0.190826000	-1.742129000
1	4.773397000	0.947398000	-3.369335000
1	0.518938000	1.199915000	-2.915963000
1	2.593052000	2.003570000	-4.035683000
1	4.776474000	-0.888525000	-1.736001000
6	-2.362059000	-1.303535000	1.396411000
6	-3.650728000	-1.053387000	1.891318000
6	-3.821436000	-0.039001000	2.819275000
6	-2.721601000	0.699203000	3.257383000
6	-1.490199000	0.398102000	2.717319000
7	-1.316983000	-0.556830000	1.785350000
1	-4.810017000	0.172896000	3.205558000
1	-4.495719000	-1.646419000	1.582164000
1	-2.821020000	1.486376000	3.991563000
1	-0.600583000	0.946634000	2.999715000
6	0.831408000	2.904462000	0.161692000
6	0.723976000	4.280302000	0.178938000
6	-0.547557000	4.842546000	0.111030000
6	-1.650289000	4.023889000	-0.023104000
6	-1.476070000	2.636331000	-0.095405000
7	-0.246209000	2.094232000	0.041048000
1	1.611354000	4.888741000	0.265686000
1	-2.635100000	4.452535000	-0.118567000
6	1.760004000	-0.704309000	2.349155000
6	2.864617000	-0.690499000	3.182266000
6	3.824583000	0.300830000	3.020028000
6	3.618140000	1.270054000	2.049683000
6	2.467605000	1.209898000	1.269274000
7	1.568436000	0.214064000	1.386173000
1	4.707349000	0.326482000	3.644624000
1	1.001228000	-1.466405000	2.437249000
1	2.965873000	-1.455113000	3.940207000
1	4.319074000	2.077950000	1.897917000
6	-2.598308000	0.807981000	-1.279433000
6	-3.700586000	0.692066000	-2.132250000
6	-3.695479000	-0.277014000	-3.118849000
6	-2.579466000	-1.096869000	-3.261675000
6	-1.532099000	-0.930288000	-2.384642000
7	-1.549201000	-0.025986000	-1.382383000
1	-4.543225000	-0.374386000	-3.784515000
1	-4.536736000	1.366855000	-2.038147000
1	-2.521105000	-1.852134000	-4.032643000
1	-0.641845000	-1.535349000	-2.451687000
7	2.567338000	-1.838838000	-0.491317000
7	-2.110138000	-2.309428000	0.468425000
6	3.837581000	-2.352890000	0.037504000
1	4.378556000	-2.962904000	-0.689179000
1	4.461303000	-1.510869000	0.330225000
1	3.633258000	-2.946257000	0.923118000
6	-3.264861000	-3.030129000	-0.083773000
1	-3.719945000	-3.707053000	0.642836000
1	-4.007529000	-2.304437000	-0.410451000
1	-2.941838000	-3.596478000	-0.950938000
1	0.606028000	-5.911539000	-0.048868000
1	-0.672648000	5.916933000	0.138713000
44	0.004882000	0.052101000	0.010403000
7	-2.578462000	1.793512000	-0.273676000
6	2.214198000	2.319764000	0.298227000
8	3.143583000	2.871421000	-0.258892000

6	-3.882408000	2.307395000	0.171826000
1	-4.323799000	3.018405000	-0.529398000
1	-4.552960000	1.462579000	0.299946000
1	-3.752808000	2.785723000	1.138404000

Table S8. Cartesian coordinates of DFT optimized geometry of $\mathbf{2^{\bullet+}}$ (^{2}GS; spin density at 0.01 a.u.).

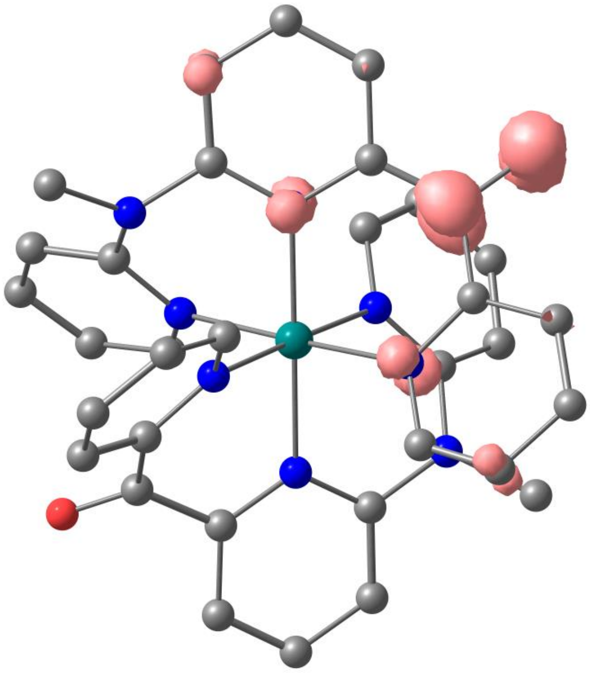

6	1.555283685	-2.597543257	-0.184758003
6	1.721079680	-3.983554254	-0.201013270
6	0.624599020	-4.807558373	-0.017739609
6	-0.627163413	-4.237347133	0.168925549
6	-0.727062552	-2.854867866	0.210328906
7	0.344446763	-2.042041028	0.050099327
6	1.518272499	1.038990844	-2.349169682
6	2.503424850	1.206281765	-3.297564323
6	3.615759661	0.371015694	-3.248940342
6	3.670805737	-0.619710480	-2.284989478
6	2.630270754	-0.737990591	-1.358753605
7	1.585572204	0.111369056	-1.371378716
6	-2.242793413	-1.176450455	1.493319146
6	-3.319353196	-1.244350247	2.375088255
6	-3.433596214	-0.297295483	3.380196409
6	-2.454654287	0.685350685	3.479769575
6	-1.427866800	0.710916825	2.554527497
7	-1.326930473	-0.188149500	1.557418386
6	0.956608530	2.857034317	0.319479651
6	0.810668324	4.246615673	0.146239720
6	-0.418428918	4.778082672	-0.165697039
6	-1.513511492	3.928138283	-0.331652845
6	-1.317955098	2.561396747	-0.204993741
7	-0.113545033	2.027519875	0.099077327
6	1.655991937	-0.819281742	2.532655014
6	2.673028804	-0.864443845	3.461350502
6	3.639657835	0.147660561	3.431852046
6	3.506079739	1.167435921	2.517839109
6	2.418137882	1.189242786	1.617095822
7	1.537910796	0.150292689	1.604253748
6	-2.392246298	0.696717941	-1.366225059
6	-3.448598918	0.541473741	-2.271331942
6	-3.361062418	-0.418418334	-3.261844859
6	-2.205000103	-1.189763497	-3.361521214
6	-1.214304621	-1.003076643	-2.424087422

7	-1.312452713	-0.111661162	-1.413399094
7	2.655586593	-1.753054686	-0.391208569
6	-2.085992373	-2.277720699	0.493576320
8	-3.074672220	-2.833106510	0.041966931
44	0.119181300	-0.010054897	0.101373956
7	-2.420288459	1.686544515	-0.386368714
6	2.262561888	2.373641484	0.769278196
8	3.269244873	3.140560608	0.621285841
6	-3.735289689	2.180575927	0.032332730
6	3.972082220	-2.248055023	0.025151795

Table S9. Cartesian coordinates of DFT optimized geometry of **2^{3+}** (^{2}GS; spin density at 0.01 a.u.).

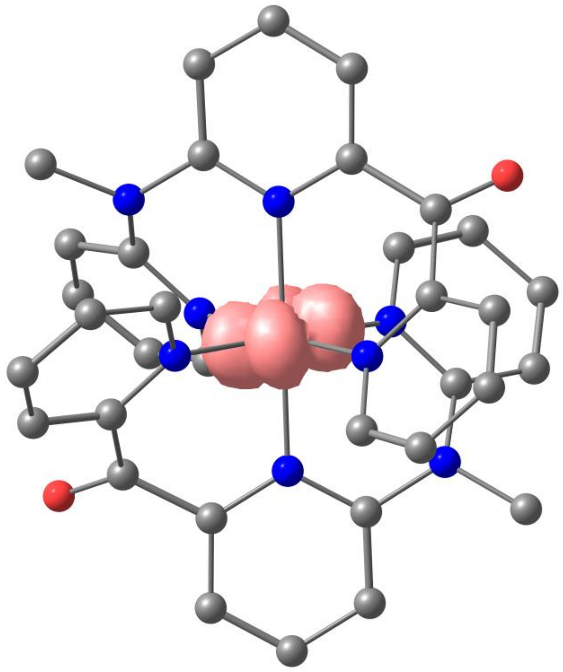

6	1.557544860	-2.620600039	-0.181142135
6	1.729238006	-4.007899053	-0.171583077
6	0.634777215	-4.826180121	0.015661001
6	-0.623176062	-4.257983458	0.193377771
6	-0.724905096	-2.881424097	0.213192813
7	0.347147155	-2.074058897	0.037228149
6	1.504495899	1.012692852	-2.373608146
6	2.509577065	1.200987875	-3.284511108
6	3.648202460	0.398220168	-3.189295187
6	3.707565034	-0.590250124	-2.228834247
6	2.637219380	-0.761293970	-1.346108915
7	1.571228325	0.075272818	-1.392088068
6	-2.242034044	-1.188855958	1.487294992
6	-3.309528491	-1.257161099	2.371132126
6	-3.431507022	-0.291241166	3.361574132
6	-2.478159283	0.714784052	3.439059440
6	-1.461244126	0.748337065	2.503199950
7	-1.345617309	-0.181017778	1.538491304
6	0.935862136	2.839447112	0.263463064
6	0.826452094	4.215761464	0.258155946
6	-0.431245061	4.779489225	0.064055848
6	-1.517802061	3.957503387	-0.151566948
6	-1.337890263	2.571641089	-0.172420765
7	-0.128018031	2.028540129	0.058504249
6	1.650966341	-0.821225939	2.517781958
6	2.648518373	-0.792560937	3.474457165
6	3.593295029	0.223466260	3.431636142
6	3.483748459	1.201496944	2.451808372
6	2.435576389	1.136551020	1.544946208

7	1.546291906	0.121319176	1.564545875
6	-2.389323123	0.724784973	-1.379234352
6	-3.443468016	0.565811132	-2.283068035
6	-3.360526361	-0.401703212	-3.262361336
6	-2.217465932	-1.199143163	-3.350164012
6	-1.230642968	-1.026174171	-2.416365138
7	-1.319147863	-0.106473108	-1.419963009
7	2.659174415	-1.779078036	-0.397489903
6	-2.085529343	-2.297133037	0.485623845
8	-3.068699396	-2.826253135	0.012993947
44	0.112776139	-0.022877920	0.059257798
7	-2.430984189	1.725989387	-0.414148932
6	2.293243055	2.257345258	0.555326014
8	3.283606215	2.796830351	0.110242967
6	-3.760578540	2.211678320	-0.001714005
6	3.979994478	-2.283450101	0.021922755

Table S10. Cartesian coordinates of DFT optimized geometry of **3**$^{\bullet+}$ (^{2}GS; spin density at 0.01 a.u.).

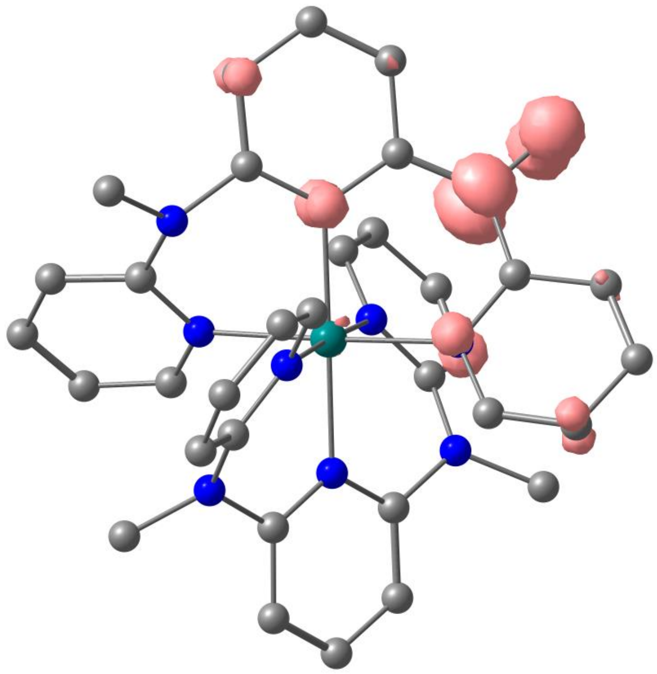

6	1.415226872	-2.562458277	-0.278060948
6	1.582390261	-3.943093361	-0.321765168
6	0.484462301	-4.758448608	-0.103660002
6	-0.751403118	-4.191107096	0.159786129
6	-0.859800315	-2.803813374	0.199562266
7	0.207923795	-2.002654365	-0.024745916
6	1.415851301	1.101464981	-2.416929241
6	2.404787269	1.267373161	-3.361939264
6	3.509393447	0.421419827	-3.317617495
6	3.551096318	-0.578001788	-2.361779976
6	2.506932237	-0.694728235	-1.438533955
7	1.470127953	0.165804991	-1.444954992
6	-2.269257038	-1.239014023	1.469146330
6	-3.292398495	-1.369957346	2.414371199
6	-3.443724137	-0.404928018	3.393947080
6	-2.549488142	0.661040282	3.438640332
6	-1.561841250	0.725264937	2.479712418
7	-1.436169062	-0.182029036	1.489167222
6	0.883106868	2.896160096	0.265505159
6	0.758385083	4.290382335	0.112089267
6	-0.461850028	4.845415185	-0.194792791
6	-1.567470108	4.013350108	-0.376854107
6	-1.392528872	2.641020989	-0.266767247

7	-0.198901853	2.084811250	0.036161856
6	1.529197364	-0.797890810	2.457649960
6	2.552652093	-0.867839046	3.378489283
6	3.542549047	0.121727172	3.341051051
6	3.422648066	1.146943003	2.431335034
6	2.325217003	1.196997350	1.541669013
7	1.423736042	0.174446984	1.531121981
6	-2.481354455	0.806027969	-1.459662944
6	-3.531958094	0.680478021	-2.376388443
6	-3.459478273	-0.279803000	-3.369343512
6	-2.323222005	-1.080081987	-3.456064022
6	-1.334278099	-0.914097076	-2.510372418
7	-1.417948960	-0.020867046	-1.503354993
7	2.520638457	-1.718027386	-0.480890883
7	-2.105387249	-2.204560357	0.461716146
6	3.833020260	-2.222035906	-0.063980175
6	-3.303144497	-2.948345969	0.059547787
44	0.005142015	0.042663857	0.022040232
7	-2.505333063	1.784393092	-0.464401191
6	2.184136232	2.387690082	0.703805212
8	3.203239007	3.142419079	0.554941831
6	-3.815925189	2.289874313	-0.051585257

Table S11. Cartesian coordinates of DFT optimized geometry of **3^{3+}** (^{2}GS; spin density at 0.01 a.u.).

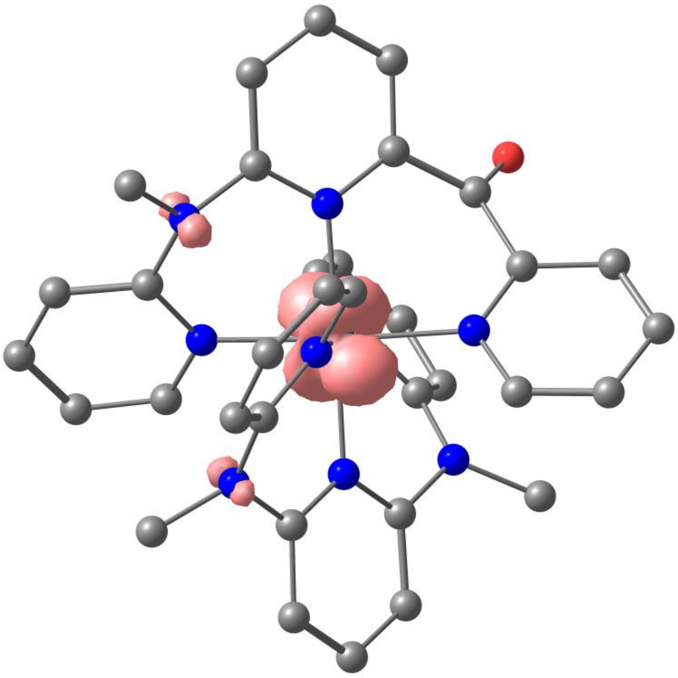

6	1.428239341	-2.570933050	-0.245554119
6	1.577469971	-3.948640197	-0.261169081
6	0.460231274	-4.747075531	-0.074483815
6	-0.781214848	-4.173256360	0.136376915
6	-0.881078003	-2.788728118	0.173589188
7	0.210543223	-2.000159294	-0.023835202
6	1.436163241	1.018506922	-2.471161972
6	2.450310271	1.185468165	-3.382509442
6	3.579003539	0.375965089	-3.270455102
6	3.623510520	-0.597793017	-2.291282983
6	2.549783952	-0.730439232	-1.407250054
7	1.498242082	0.108403018	-1.471124923
6	-2.316916329	-1.194033957	1.366238288
6	-3.428225065	-1.225504128	2.218068135
6	-3.558175122	-0.259433909	3.194696499
6	-2.565081408	0.710162218	3.344378517
6	-1.508966918	0.700097796	2.468947365

7	-1.409068307	-0.200340156	1.461129294
6	0.860034211	2.867977174	0.211421128
6	0.738862146	4.236036361	0.288295236
6	-0.535046883	4.799078308	0.187924071
6	-1.623869290	3.985915443	-0.027008148
6	-1.437885184	2.602231239	-0.149647093
7	-0.210427862	2.055060376	0.013701987
6	1.600965970	-0.727138224	2.451354338
6	2.639478967	-0.714704147	3.366120294
6	3.615676051	0.266385181	3.266425417
6	3.495156462	1.229604193	2.273871993
6	2.412560354	1.173189136	1.406094860
7	1.494583880	0.189383012	1.475032897
6	-2.488758174	0.785121234	-1.410487032
6	-3.552940501	0.644543181	-2.306866195
6	-3.473744363	-0.303242906	-3.306891072
6	-2.323510407	-1.083661871	-3.423344463
6	-1.321449255	-0.910789189	-2.501471128
7	-1.412649250	-0.021969852	-1.483781256
7	2.538408229	-1.736747031	-0.435528222
7	-2.126072258	-2.185567127	0.403484952
6	3.845338448	-2.258726940	-0.000665176
6	-3.318924033	-2.935353080	-0.030138232
44	0.022920783	0.024588221	-0.001209170
7	-2.525553455	1.780007271	-0.428353108
6	2.235221416	2.285172044	0.418289215
8	3.198036137	2.829064125	-0.079306207
6	-3.857108408	2.245341931	0.003446002
